# Supplementary material for: One-step synthesis of sequence-controlled multiblock polymers with up to 11 segments from monomer mixture
Source: Nat Commun. 2022 Jan 10;13:163. doi: 10.1038/s41467-021-27830-3 (PMC8748456; doi:10.1038/s41467-021-27830-3)
Supplement: Supplementary file 1 — Supplementary Information [file 41467_2021_27830_MOESM1_ESM.pdf]

Supplementary Information

One-step synthesis of sequence-controlled multiblock polymers with up to 11 segments from monomer mixture

Xiachao Xia,[*,1,2] Ryota Suzuki,[3] Tianle Gao,[3] Takuya Isono,[*,2] Toshifumi Satoh[*,2]

[1] College of Materials Science and Engineering, Chongqing University of Technology, Chongqing 400054, China

[2] Division of Applied Chemistry, Faculty of Engineering, Hokkaido University, Sapporo 060-8628, Japan

[3] Graduate School of Chemical Sciences and Engineering, Hokkaido University, Sapporo 060-8628, Japan

*Email: xiaxiaoc@cqut.edu.cn (X.X.).

*Email: isono.t@eng.hokudai.ac.jp (T.I.).

*Email: satoh@eng.hokudai.ac.jp (T.S.).

Supplementary methods

Chemicals

Unless otherwise stated, all chemicals are used without further purification. L-Lactide (L-LA; >98%, Tokyo Kasei Kogyo Co., Ltd. (TCI)) was purified by recrystallization from dry toluene. 1,2-Butylene oxide (BO; >99.0%, TCI), ethyl glycidyl ether (EGE; >98.0%, TCI), and 3-phenyl-1-propanol (PPA; >98%, TCI) were distilled over CaH_2 under reduced pressure and stored under nitrogen atmosphere. 5-norbornene-endo-2,3-dicarboxylic anhydride (NA; > 99%, Sigma−Aldrich), diglycolic anhydride (DGA; >98.0%, TCI), succinic anhydride (SA; >95.0%, TCI), Trimellitic Anhydride (TA; >97.0%, TCI) were recrystallized and then purified by the way of sublimation before use. Cesium pivalate (>97.0%, TCI) was dried by heating at 100°C under high vacuum for at least 72 h prior to use. Poly(ethylene glycol) monomethyl ether (HO-PEG-OH; typical M_n = 2000, Sigma−Aldrich) was dried by azeotropic distillation in benzene. *α*-Terpinene (>99.0%, TCI), Maleic Anhydride (MA, >99.0%, TCI), and 1,4-benzenedimethanol (BDM; >99.0%, TCI) was used as received. All manipulations were performed using a standard Schlenk technique or in a nitrogen-filled UNILAB Plus glovebox unless otherwise mentioned.

Instruments.

The polymerization was carried out in an MBRAUN stainless steel glovebox that is equipped with a gas purification system (molecular sieves and copper catalyst) in a dry argon atmosphere (H_2O, O_2 <0.1 ppm). An MB-MO-SE 1 and MB-OX-SE 1 were used to monitor the moisture and oxygen contents in the glovebox, respectively. ^1H spectra were recorded at 25 °C on a JEOL JNMA400II instrument (400 MHz) using chloroform-d_1 as the solvent and chemical shifts were referenced to an internal standard. DOSY NMR analyses were carried out at 30 ºC on a JEOL JNMA400II instrument (400 MHz) with at least 15 gradient increments using the ledbpgp2s sequence. The size exclusion chromatography (SEC) was conducted in THF at 40 °C and a flow rate of 1.0 mL min^{-1} using a Shodex GPC-101 system equipped with a Shodex K-G guard column and a set of two Shodex K-805L columns (linear, 8 mm × 300 mm; bead size, 5 μm; exclusion limit, 4 × 10^6). The

polystyrene standard curve ranging from 1200 to 1320000 was used for calibration to achieve the molecular weight ($M_{n,SEC}$) and polydispersity index ($Đ$) of the polymers.

The polymerization from the mixture of diglycolic anhydride (DGA), 5-norbornene-endo-2,3-dicarboxylic anhydride (NA), and ethyl glycidyl ether (EGE) catalyzed by the cesium pivalate

A typical procedure is presented as follows: In an argon-filled glovebox, cesium pivalate (0.02 mmol, 1 equiv.), BDM (0.04 mmol, 2 equiv.), DGA (0.5 mmol, 25 equiv.), NA (0.5 mmol, 25 equiv.), and EGE (3 mmol, 150 equiv.) were placed in an oven-dried reaction vessel with a magnetic stir. The reaction mixture was stirred at 100 °C under an argon atmosphere in an oil bath. During polymerization, a crude aliquot was time-regularly obtained from the system by a syringe in an argon flow and monitored by ^{1}H NMR spectroscopy and SEC to determine monomer conversion and molar mass. After the defined time, the polymerization was terminated by diluting the reaction mixture with dichloromethane (CH_2Cl_2). The reaction mixture was purified by reprecipitation from a CH_2Cl_2 solution into cold methanol. The purified polymers were dried under vacuum at room temperature for the next analysis.

The polymerization from the mixture of DGA, succinic anhydride (SA), NA, *rac-cis-endo*-1-isopropyl-4-methyl-bicyclo[2.2.2]oct-5-ene-2,3-dicarboxylic anhydride (DPMA) and 1,2-Butylene oxide (BO) catalyzed by the cesium pivalate

A typical procedure is presented as follows: In an argon-filled glovebox, cesium pivalate (0.02 mmol, 1 equiv.), BDM (0.04 mmol, 2 equiv.), DGA (0.5 mmol, 25 equiv.), SA (0.5 mmol, 25 equiv.), NA (0.5 mmol, 25 equiv.), DPMA (0.25 mmol, 12.5 equiv.), and BO (5 mmol, 250 equiv.) were placed in an oven-dried reaction vessel with a magnetic stir. The reaction mixture was stirred at 100 °C under an argon atmosphere in an oil bath. During polymerization, a crude aliquot was time-regularly obtained from the system by a syringe in an argon flow and monitored by ^{1}H NMR spectroscopy and SEC to determine monomer conversion and molar mass. After the defined time, the polymerization was terminated by

diluting the reaction mixture with dichloromethane (CH_2Cl_2). The reaction mixture was purified by reprecipitation from a CH_2Cl_2 solution into cold methanol. The purified polymers were dried under vacuum at room temperature for the next analysis.

The polymerization from the mixture of DGA, SA, NA, and EGE catalyzed by the cesium pivalate

A typical procedure is presented as follows: In an argon-filled glovebox, cesium pivalate (0.02 mmol, 1 equiv.), BDM (0.04 mmol, 2 equiv.), DGA (0.5 mmol, 25 equiv.), SA (0.5 mmol, 25 equiv.), NA (0.5 mmol, 25 equiv.), and EGE (5 mmol, 250 equiv.) were placed in an oven-dried reaction vessel with a magnetic stir. The reaction mixture was stirred at 100 °C under an argon atmosphere in an oil bath. During polymerization, a crude aliquot was time-regularly obtained from the system by a syringe in an argon flow and monitored by ^{1}H NMR spectroscopy and SEC to determine monomer conversion and molar mass. After the defined time, the polymerization was terminated by diluting the reaction mixture with dichloromethane (CH_2Cl_2). The reaction mixture was purified by reprecipitation from a CH_2Cl_2 solution into cold methanol. The purified polymers were dried under vacuum at room temperature for the next analysis.

The polymerization from the mixture of DPMA, L-Lactide (L-LA), and EGE catalyzed by the cesium pivalate

A typical procedure is presented as follows: In an argon-filled glovebox, cesium pivalate (0.02 mmol, 1 equiv.), BDM (0.04 mmol, 2 equiv.), DPMA (0.5 mmol, 25 equiv.), L-LA (1.0 mmol, 50 equiv.), and EGE (5 mmol, 250 equiv.) were placed in an oven-dried reaction vessel with a magnetic stir. The reaction mixture was stirred at 100 °C under an argon atmosphere in an oil bath. During polymerization, a crude aliquot was time-regularly obtained from the system by a syringe in an argon flow and monitored by ^{1}H NMR spectroscopy and SEC to determine monomer conversion and molar mass. After the defined time, the polymerization was terminated by diluting the reaction mixture with dichloromethane (CH_2Cl_2). The reaction mixture was purified by reprecipitation from a

CH_2Cl_2 solution into cold methanol. The purified polymers were dried under vacuum at room temperature for the next analysis.

The polymerization from the mixture of DPMA and BO catalyzed by the cesium pivalate

A typical procedure is presented as follows: In an argon-filled glovebox, cesium pivalate (0.02 mmol, 1 equiv.), BDM (0.04 mmol, 2 equiv.), DPMA (0.5 mmol, 25 equiv.), and BO (5 mmol, 250 equiv.) were placed in an oven-dried reaction vessel with a magnetic stir. The reaction mixture was stirred at 100 °C under an argon atmosphere in an oil bath. During polymerization, a crude aliquot was time-regularly obtained from the system by a syringe in an argon flow and monitored by ^1H NMR spectroscopy and SEC to determine monomer conversion and molar mass. After the defined time, the polymerization was terminated by diluting the reaction mixture with dichloromethane (CH_2Cl_2). The reaction mixture was purified by reprecipitation from a CH_2Cl_2 solution into cold methanol. The purified polymers were dried under vacuum at room temperature for the next analysis.

The polymerization from the mixture of DPMA, L-Lactide (L-LA), and BO catalyzed by the cesium pivalate

A typical procedure is presented as follows: In an argon-filled glovebox, cesium pivalate (0.02 mmol, 1 equiv.), BDM (0.04 mmol, 2 equiv.), DPMA (0.5 mmol, 25 equiv.), L-LA (1.0 mmol, 50 equiv.), and BO (5 mmol, 250 equiv.) were placed in an oven-dried reaction vessel with a magnetic stir. The reaction mixture was stirred at 100 °C under an argon atmosphere in an oil bath. During polymerization, a crude aliquot was time-regularly obtained from the system by a syringe in an argon flow and monitored by ^1H NMR spectroscopy and SEC to determine monomer conversion and molar mass. After the defined time, the polymerization was terminated by diluting the reaction mixture with dichloromethane (CH_2Cl_2). The reaction mixture was purified by reprecipitation from a CH_2Cl_2 solution into cold methanol. The purified polymers were dried under vacuum at room temperature for the next analysis.

The polymerization from the mixture of DGA, SA, NA, L-LA, DPMA and BO catalyzed by the cesium pivalate

A typical procedure is presented as follows: In an argon-filled glovebox, cesium pivalate (0.02 mmol, 1 equiv.), BDM (0.04 mmol, 2 equiv.), DGA (0.5 mmol, 25 equiv.), SA (0.5 mmol, 25 equiv.), NA (0.5 mmol, 25 equiv.), L-LA (1.5 mmol, 75 equiv.), DPMA (0.25 mmol, 12.5 equiv.), and BO (7 mmol, 350 equiv.) were placed in an oven-dried reaction vessel with a magnetic stir. The reaction mixture was stirred at 80 or 100 °C under an argon atmosphere in an oil bath. During polymerization, a crude aliquot was time-regularly obtained from the system by a syringe in an argon flow and monitored by ^{1}H NMR spectroscopy and SEC to determine monomer conversion and molar mass. After the defined time, the polymerization was terminated by diluting the reaction mixture with dichloromethane (CH_2Cl_2). The reaction mixture was purified by reprecipitation from a CH_2Cl_2 solution into cold methanol. The purified polymers were dried under vacuum at room temperature for the next analysis.

The polymerization from the mixture of DGA, SA, L-LA, DPMA and BO catalyzed by the cesium pivalate

A typical procedure is presented as follows: In an argon-filled glovebox, cesium pivalate (0.02 mmol, 1 equiv.), BDM (0.04 mmol, 2 equiv.), DGA (0.5 mmol, 25 equiv.), SA (0.5 mmol, 25 equiv.), L-LA (1.5 mmol, 75 equiv.), DPMA (0.25 mmol, 12.5 equiv.), and BO (7 mmol, 350 equiv.) were placed in an oven-dried reaction vessel with a magnetic stir. The reaction mixture was stirred at 100 °C under an argon atmosphere in an oil bath. During polymerization, a crude aliquot was time-regularly obtained from the system by a syringe in an argon flow and monitored by ^{1}H NMR spectroscopy and SEC to determine monomer conversion and molar mass. After the defined time, the polymerization was terminated by diluting the reaction mixture with dichloromethane (CH_2Cl_2). The reaction mixture was purified by reprecipitation from a CH_2Cl_2 solution into cold methanol. The purified polymers were dried under vacuum at room temperature for the next analysis.

The polymerization from the mixture of DGA, SA, NA, L-LA, DPMA and BO initiated

by the monomethyl ether (PEG2000)

A typical procedure is presented as follows: In an argon-filled glovebox, cesium pivalate (0.02 mmol, 1 equiv.), PEG2000 (0.04 mmol, 2 equiv.), DGA (0.5 mmol, 25 equiv.), SA (0.5 mmol, 25 equiv.), NA (0.5 mmol, 25 equiv.), L-LA (1.5 mmol, 75 equiv.), DPMA (0.25 mmol, 12.5 equiv.), and BO (7 mmol, 350 equiv.) were placed in an oven-dried reaction vessel with a magnetic stir. The reaction mixture was stirred at 80 or 100 °C under an argon atmosphere in an oil bath. During polymerization, a crude aliquot was time-regularly obtained from the system by a syringe in an argon flow and monitored by ^{1}H NMR spectroscopy and SEC to determine monomer conversion and molar mass. After the defined time, the polymerization was terminated by diluting the reaction mixture with dichloromethane (CH_2Cl_2). The reaction mixture was purified by reprecipitation from a CH_2Cl_2 solution into cold methanol. The purified polymers were dried under vacuum at room temperature for the next analysis.

The polymerization from the mixture of TA, NA, L-LA, DPMA and BO catalyzed by the cesium pivalate

A typical procedure is presented as follows: In an argon-filled glovebox, cesium pivalate (0.02 mmol, 1 equiv.), BDM (0.04 mmol, 2 equiv.), TA (0.5 mmol, 25 equiv.), NA (0.5 mmol, 25 equiv.), L-LA (1.0 mmol, 50 equiv.), DPMA (0.25 mmol, 12.5 equiv.), and BO (7 mmol, 350 equiv.) were placed in an oven-dried reaction vessel with a magnetic stir. The reaction mixture was stirred at 80 °C under an argon atmosphere in an oil bath. During polymerization, a crude aliquot was time-regularly obtained from the system by a syringe in an argon flow and monitored by ^{1}H NMR spectroscopy and SEC to determine monomer conversion and molar mass. After the defined time, the polymerization was terminated by diluting the reaction mixture with dichloromethane (CH_2Cl_2). The reaction mixture was purified by reprecipitation from a CH_2Cl_2 solution into cold methanol. The purified polymers were dried under vacuum at room temperature for the next analysis.

The polymerization from the mixture of TA, NA, and BO catalyzed by the cesium

pivalate

A typical procedure is presented as follows: In an argon-filled glovebox, cesium pivalate (0.02 mmol, 1 equiv.), BDM (0.04 mmol, 2 equiv.), TA (0.5 mmol, 25 equiv.), NA (0.5 mmol, 25 equiv.), and BO (3 mmol, 150 equiv.) were placed in an oven-dried reaction vessel with a magnetic stir. The reaction mixture was stirred at 80 °C under an argon atmosphere in an oil bath. During polymerization, a crude aliquot was time-regularly obtained from the system by a syringe in an argon flow and monitored by ^{1}H NMR spectroscopy and SEC to determine monomer conversion and molar mass. After the defined time, the polymerization was terminated by diluting the reaction mixture with dichloromethane (CH_2Cl_2). The reaction mixture was purified by reprecipitation from a CH_2Cl_2 solution into cold methanol. The purified polymers were dried under vacuum at room temperature for the next analysis.

The polymerization from the mixture of TA, L-LA, DPMA and BO catalyzed by the cesium pivalate

A typical procedure is presented as follows: In an argon-filled glovebox, cesium pivalate (0.02 mmol, 1 equiv.), BDM (0.04 mmol, 2 equiv.), TA (0.5 mmol, 25 equiv.), L-LA (1.0 mmol, 50 equiv.), DPMA (0.25 mmol, 12.5 equiv.), and BO (7 mmol, 350 equiv.) were placed in an oven-dried reaction vessel with a magnetic stir. The reaction mixture was stirred at 80 °C under an argon atmosphere in an oil bath. During polymerization, a crude aliquot was time-regularly obtained from the system by a syringe in an argon flow and monitored by ^{1}H NMR spectroscopy and SEC to determine monomer conversion and molar mass. After the defined time, the polymerization was terminated by diluting the reaction mixture with dichloromethane (CH_2Cl_2). The reaction mixture was purified by reprecipitation from a CH_2Cl_2 solution into cold methanol. The purified polymers were dried under vacuum at room temperature for the next analysis.

Supplementary discussion

The terpolymerization of DGA/NA/EGE

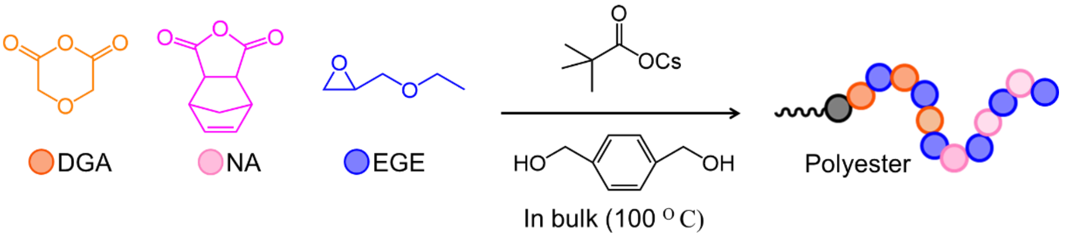

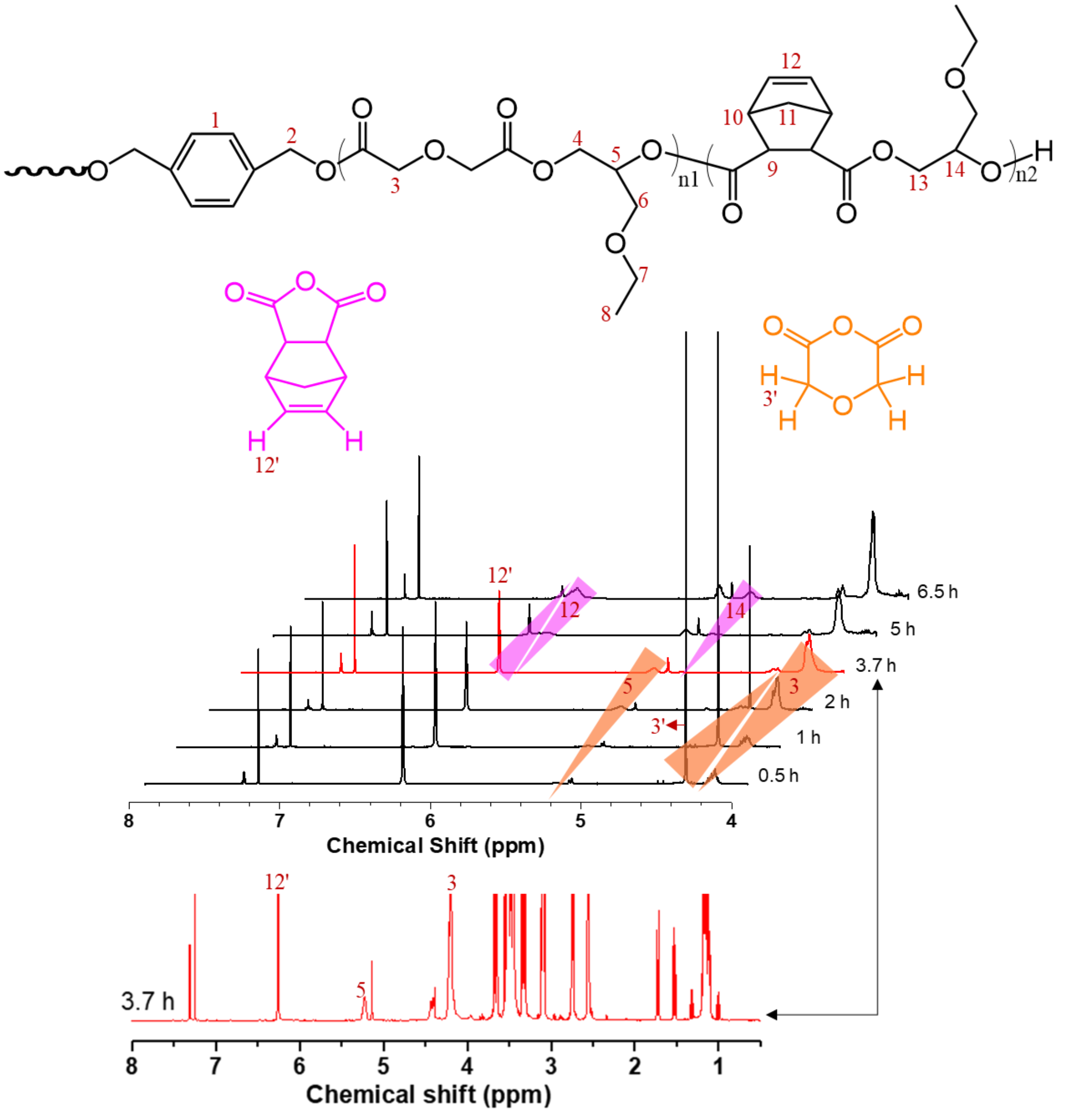

Supplementary Figure 1 | The ^{1}H NMR ($CDCl_3$) spectra of crude aliquots withdrawn from the reaction system for monitoring the conversion of DGD and NA and the formation of resultant triblock polymers (entry 1 in Table 1).

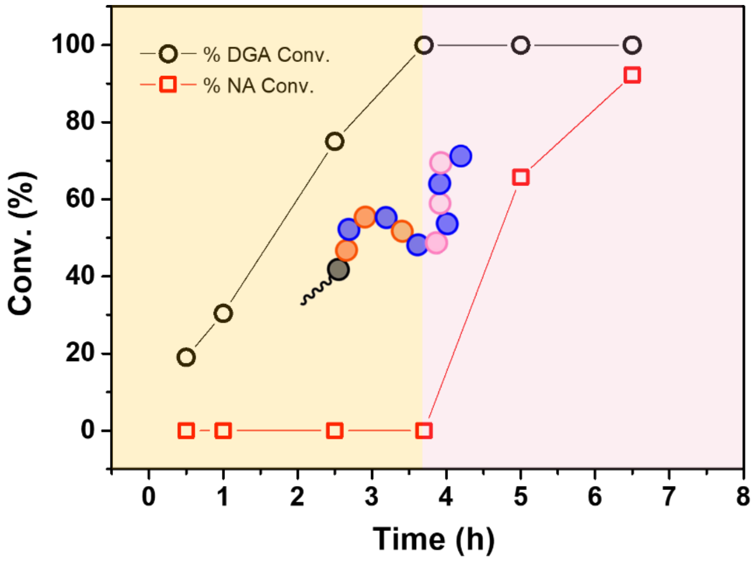

Supplementary Figure 2 | The plots of monomer conversion versus time. The polymerization of DGA, NA with EGE was performed at 100 ℃ (entry 1 in Table 1).

Calculation method for degree of polymerization (DP) of each block:

$DP_{P(DGA\text{-}alt\text{-}EGE)}$ = 12.5 × conv. of DGA = 12.5 per block

$DP_{P(NA\text{-}alt\text{-}EGE)}$ = (12.5 × conv. of NA)/2= 5.8 per block

P(NA-*alt*-EGE)$_{5.8}$-*b*-P(DGA-*alt*-EGE)$_{12.5}$-*b*-P(NA-*alt*-EGE)$_{5.8}$

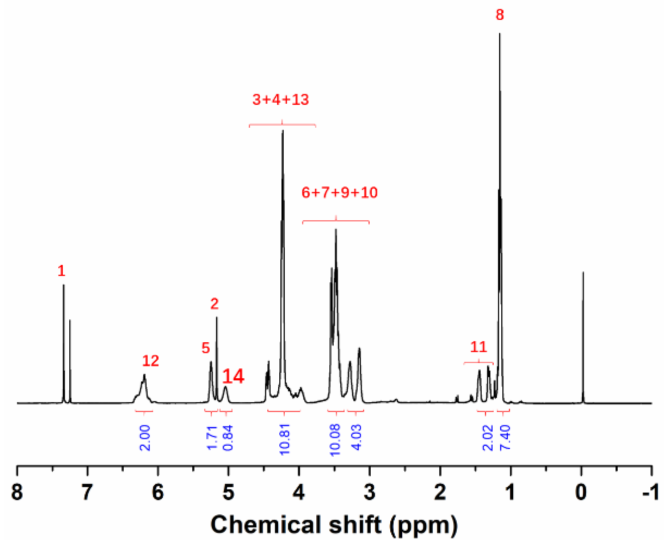

Supplementary Figure 3 | The ^{1}H NMR ($CDCl_3$) spectrum of the resultant triblock polymer isolated from the mixture by precipitation (entry 1 in Table 1).

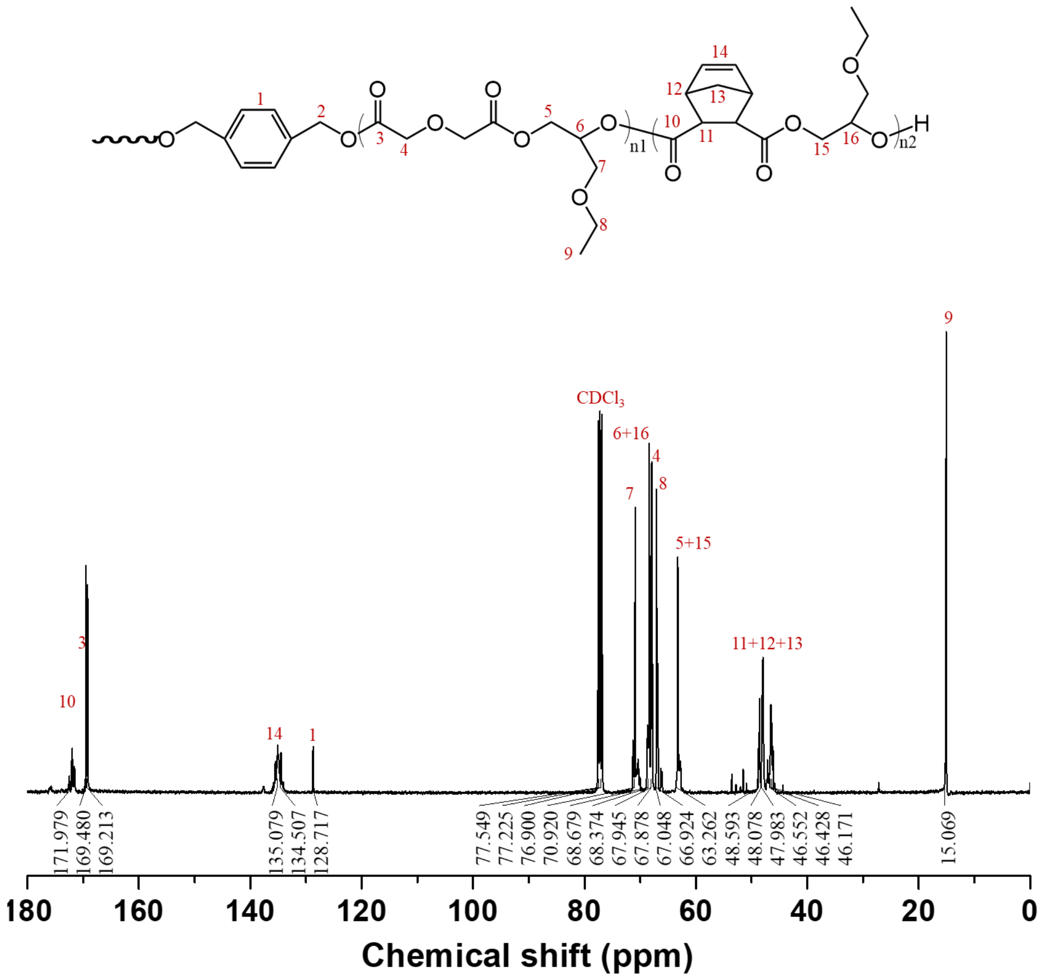

Supplementary Figure 4 | The ^{13}C NMR ($CDCl_3$) spectrum of the resultant triblock polymer (entry 1 in Table 1).

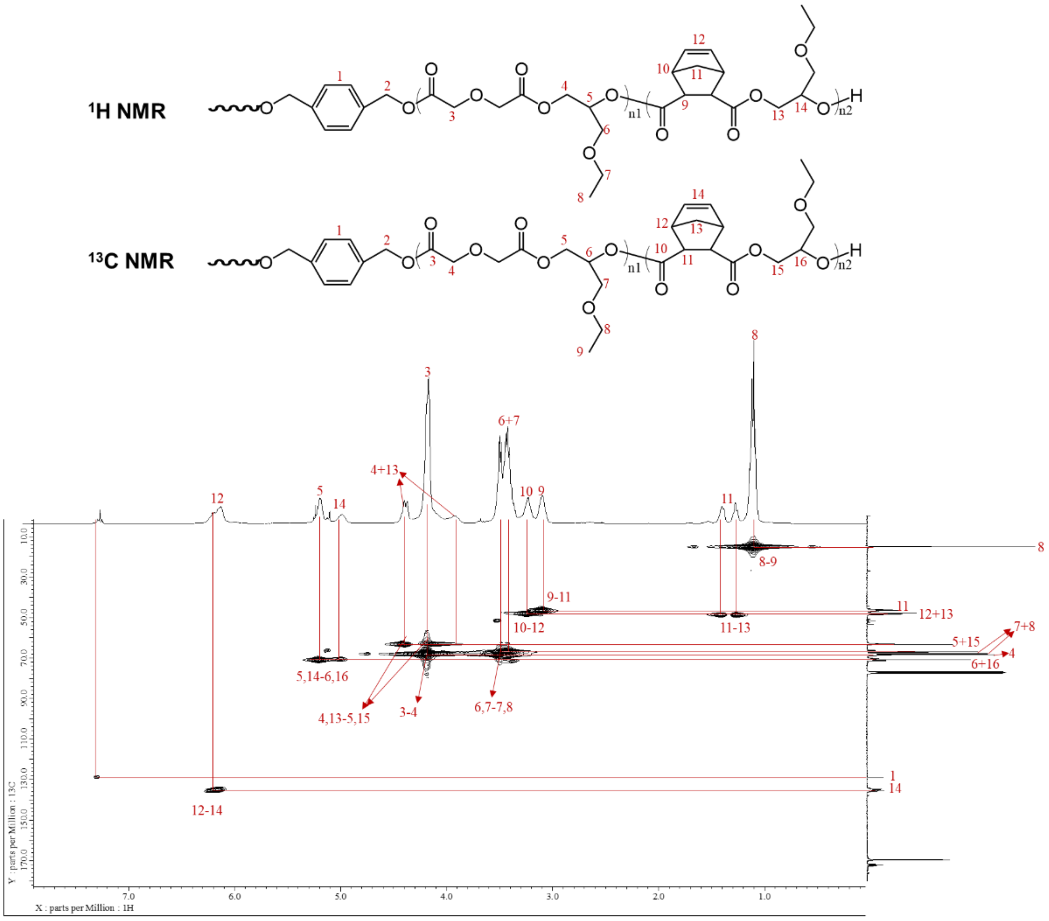

Supplementary Figure 5 | The HMQC NMR spectrum of the resultant the resultant triblock polymer isolated from the
mixture by precipitation (entry 1 in Table 1).

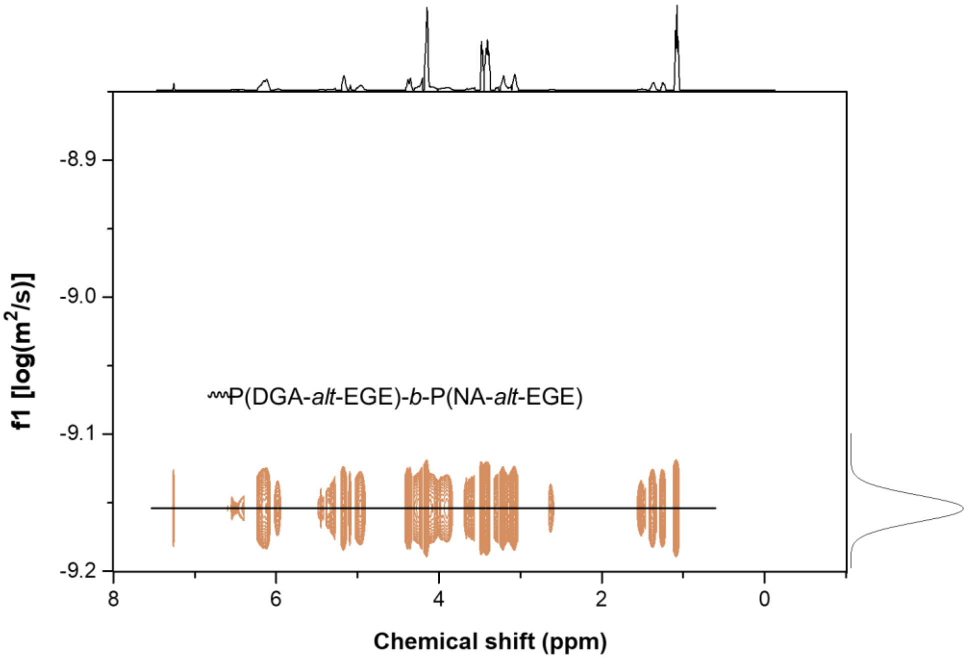

Supplementary Figure 6 | The DOSY NMR ($CDCl_3$) spectrum of the resultant triblock polymer (entry 1

in Table 1).

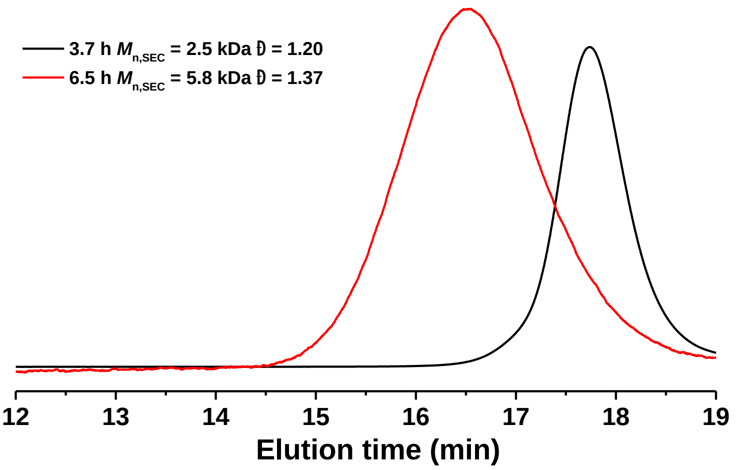

Supplementary Figure 7 | The SEC (THF) trace of the resultant triblock polymer (entry 1 in Table 1).

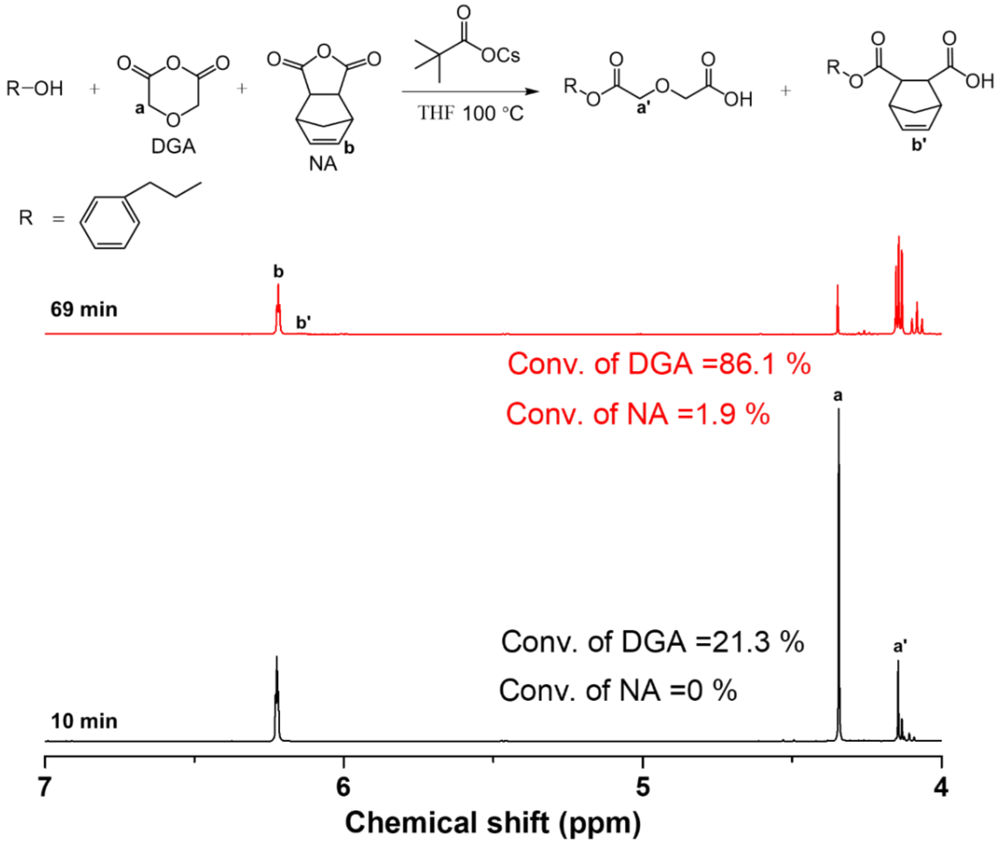

Supplementary Figure 8 | ^{1}H NMR ($CDCl_3$) spectrum of the product obtained from a mixture of DGA and NA with cesium pivalate and 3-phenyl-1-propanol (PPA) heated in THF at 100 °C for 10 min and 69 min, respectively, with the feed ratio of [*t*-$BuCO_2Cs$]/[initiator]/[DGA]$_0$/[NA]$_0$ being 0.02/1/1/1. From the result, DGA is far more reactive than NA.

Reactivity ratio of DGA and NA

Since the nonterminal copolymerization kinetics are common in coordination−insertion, ionic, and pseudo ionic type polymerization mechanisms, we employed the simple model for compositional drift reported by Beckingham et al. (BSL) to determine the reactivity ration.[1,2] Here, equations 1 and 2 enable a simple and accurate determination of reactivity ratios at all conversions for copolymerizations that can be described by the nonterminal model of copolymerization kinetics. Here, p_A and p_B are the respective conversions of A and B monomer with p_A = 1 −($A(t)/A_0$).

$$p_{AB}(p_A) = 1 - n_A(1 - p_A) - (1 - n_A)(1 - p_A)^{r_B} \quad (1)$$

$$p_{AB}(p_B) = 1 - (1 - n_A)(1 - p_B) - n_A(1 - p_B)^{r_A} \quad (2)$$

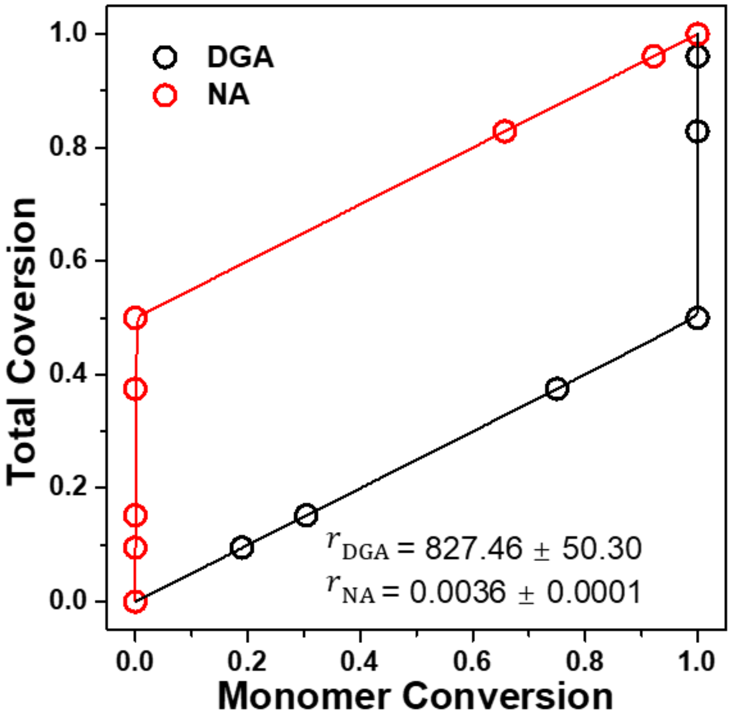

Supplementary Figure 9 | Total polymerization conversion plotted against monomer conversion and the data were obtained from time-resolved ^{1}H NMR spectra of a cesium pivalate-catalyzed copolymerization of DGA/NA/EGE with a molar ratio of 25/25/150 at 100 °C. Solid black and red lines represent fits to the experimental data using the nonterminal model, eqs 1 and 2.

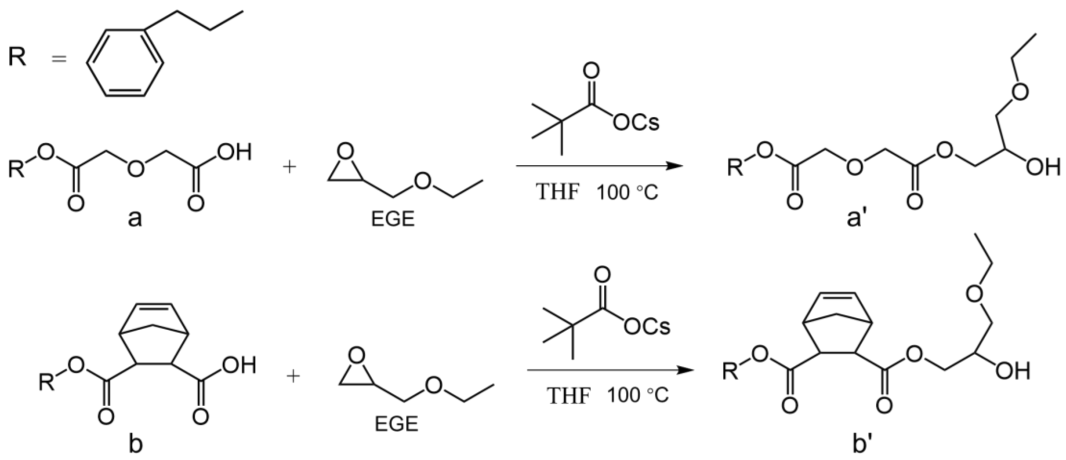

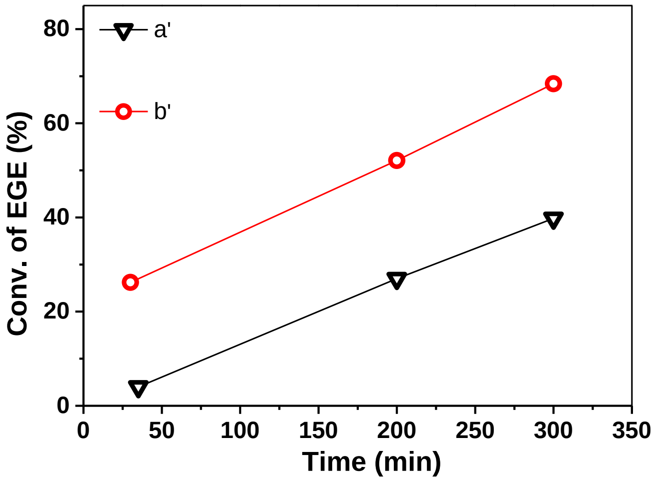

Supplementary Figure 10 | ^{1}H NMR ($CDCl_3$) spectrum of the products obtained from a mixture of mono ester, cesium pivalate and EGE heated in THF at 100 °C with the feed ratio of [*t*-$BuCO_2Cs$]/[mono ester (a or b)]$_0$/[EGE]$_0$ being 0.5/1/1/1.

The reactivity difference between the different anhydrides

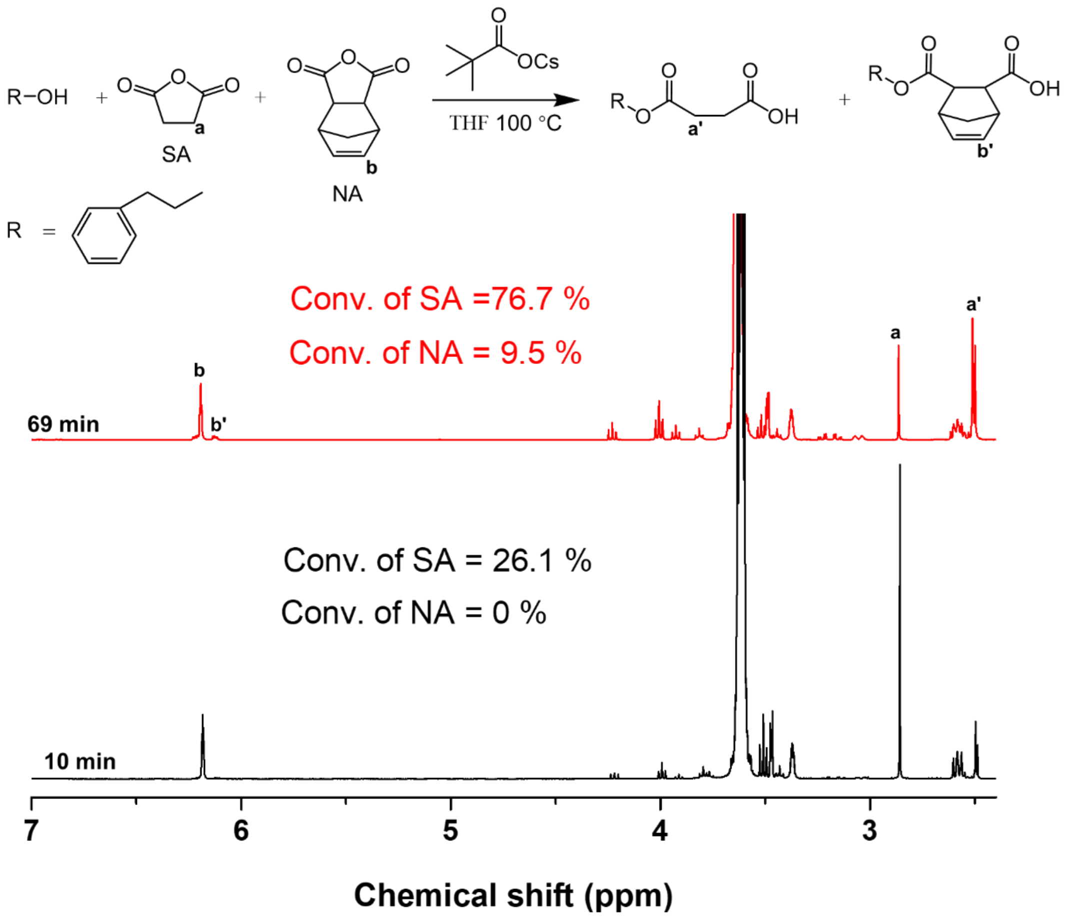

Supplementary Figure 11 | ^{1}H NMR ($CDCl_3$) spectrum of the product obtained from a mixture of SA and NA with cesium pivalate and PPA heated in THF at 100 °C for 10 min and 69 min, respectively, with the feed ratio of [*t*-$BuCO_2Cs$]/[initiator]/$[SA]_0$/$[NA]_0$ being 0.02/1/1/1. From the result, SA is far more reactive than NA.

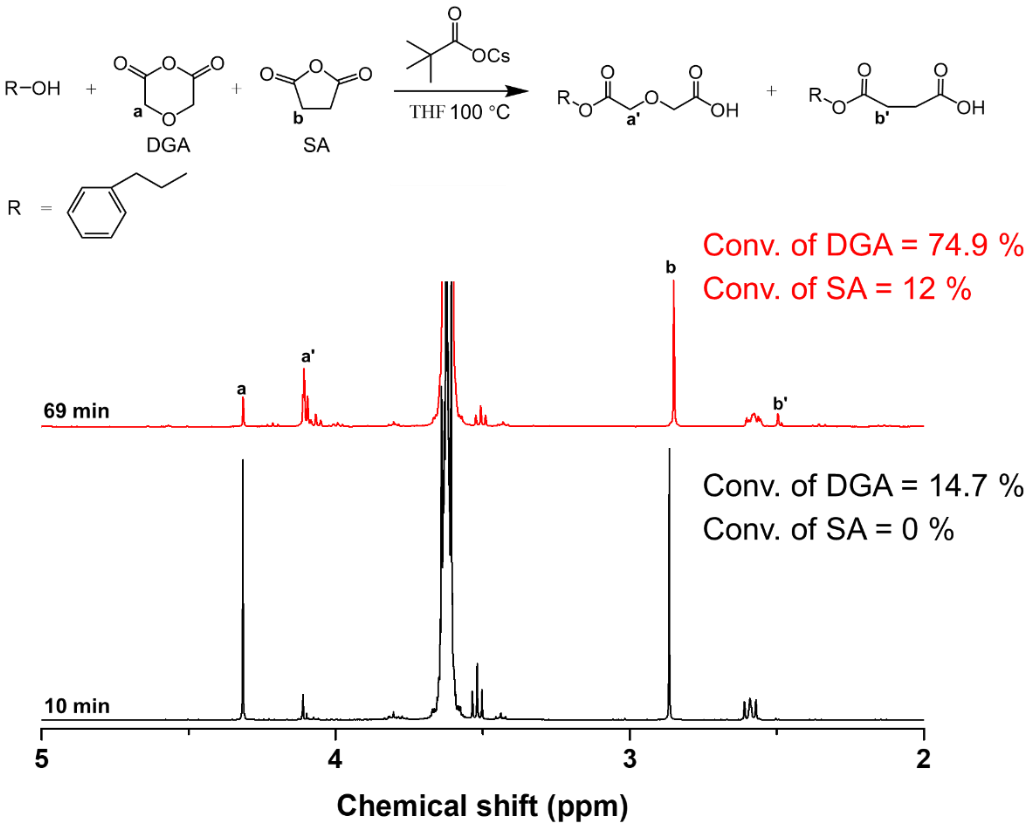

Supplementary Figure 12 | ^{1}H NMR ($CDCl_3$) spectrum of the product obtained from a mixture of DGA and SA with cesium pivalate and PPA heated in THF at 100 °C for 10 min and 69 min, respectively, with the feed ratio of [*t*-$BuCO_2Cs$]/[initiator]/[DGA]$_0$/[SA]$_0$ being 0.02/1/1/1. From the result, DGA is far more reactive than SA.

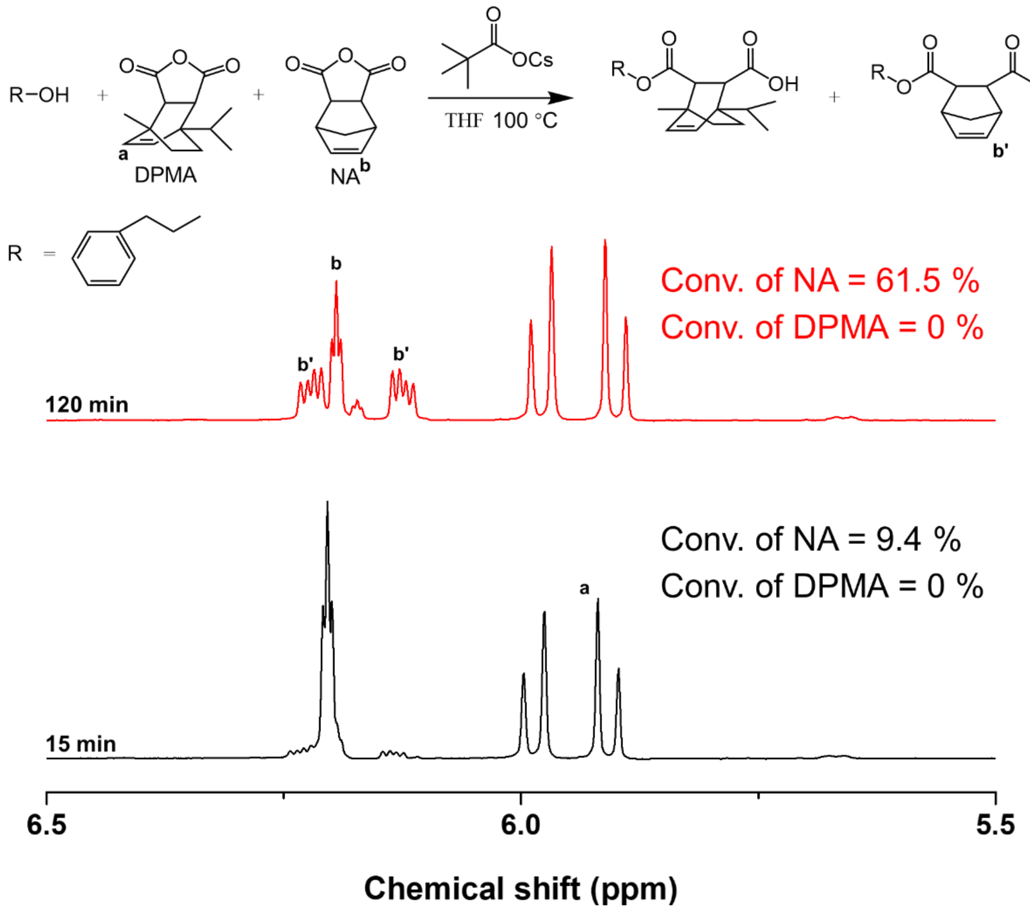

Supplementary Figure 13 | ^{1}H NMR ($CDCl_3$) spectrum of the product obtained from a mixture of DPMA and NA with cesium pivalate and PPA heated in THF at 100 °C for 15 min and 120 min, respectively, with the feed ratio of [*t*-$BuCO_2Cs$]/[initiator]/$[DPMA]_0$/$[NA]_0$ being 0.02/1/1/1. From the result, NA is far more reactive than DPMA. According to comprehensive analysis of these results in Figure S5–S8, a final reactivity trend of these anhydrides was achieved in the following order DGA >> succinic anhydride (SA) >> NA >> DPMA.

Reactivity ratio of different anhydrides

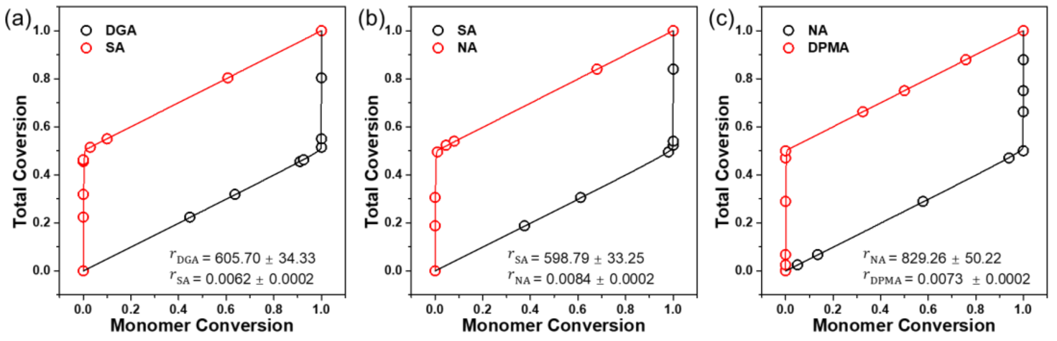

Supplementary Figure 14 | Total polymerization conversion plotted against monomer conversion and the data were obtained from time-resolved ^{1}H NMR spectra of a cesium pivalate-catalyzed copolymerization of DGA/SA/BO, SA/NA/BO, and NA/DPMA/BO with a molar ratio of 25/25/150 at 80 °C, respectively. Solid black and red lines represent fits to the experimental data using the nonterminal model, eqs 1 and 2.

Synthesis of the DPMA

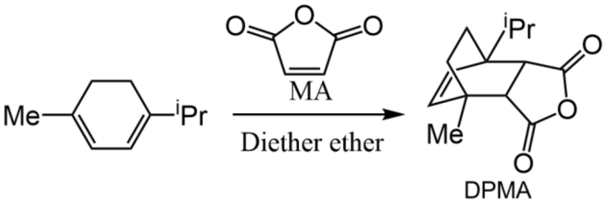

Group	a	b	c
^{1}H (δ ppm)	7.02	5.57	6.00, 6.08

rac-cis-endo-1-isopropyl-4-methyl-bicyclo[2.2.2]oct-5-ene-2,3-dicarboxylic anhydride: Synthesized according to literature procedure.[3]

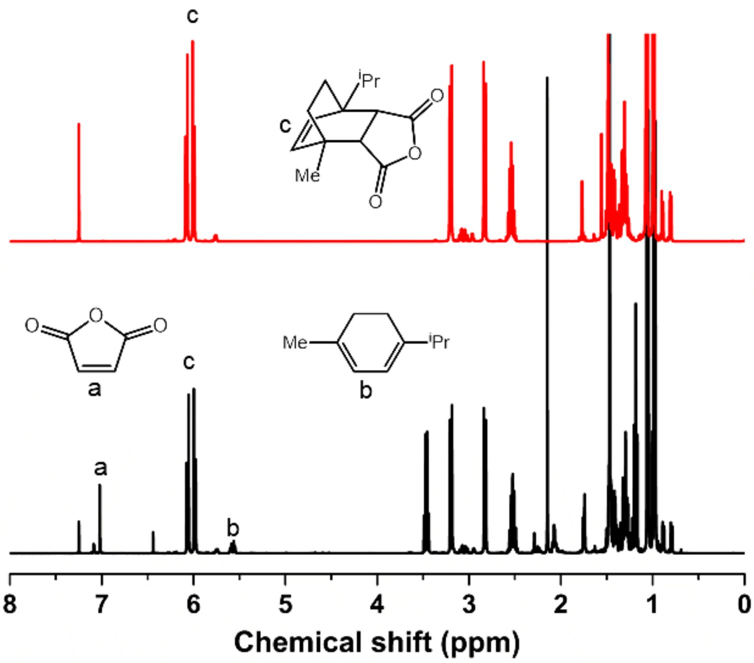

Supplementary Figure 15 | Black line: the ^{1}H NMR ($CDCl_3$) spectra in situ of crude aliquots withdrawn from the reaction system for monitoring the conversion of SA and the formation of DPMA; Red line: the ^{1}H NMR ($CDCl_3$) spectra of purified monomer by running column.

The polymerization of DGA/SA/NA/DPMA/BO

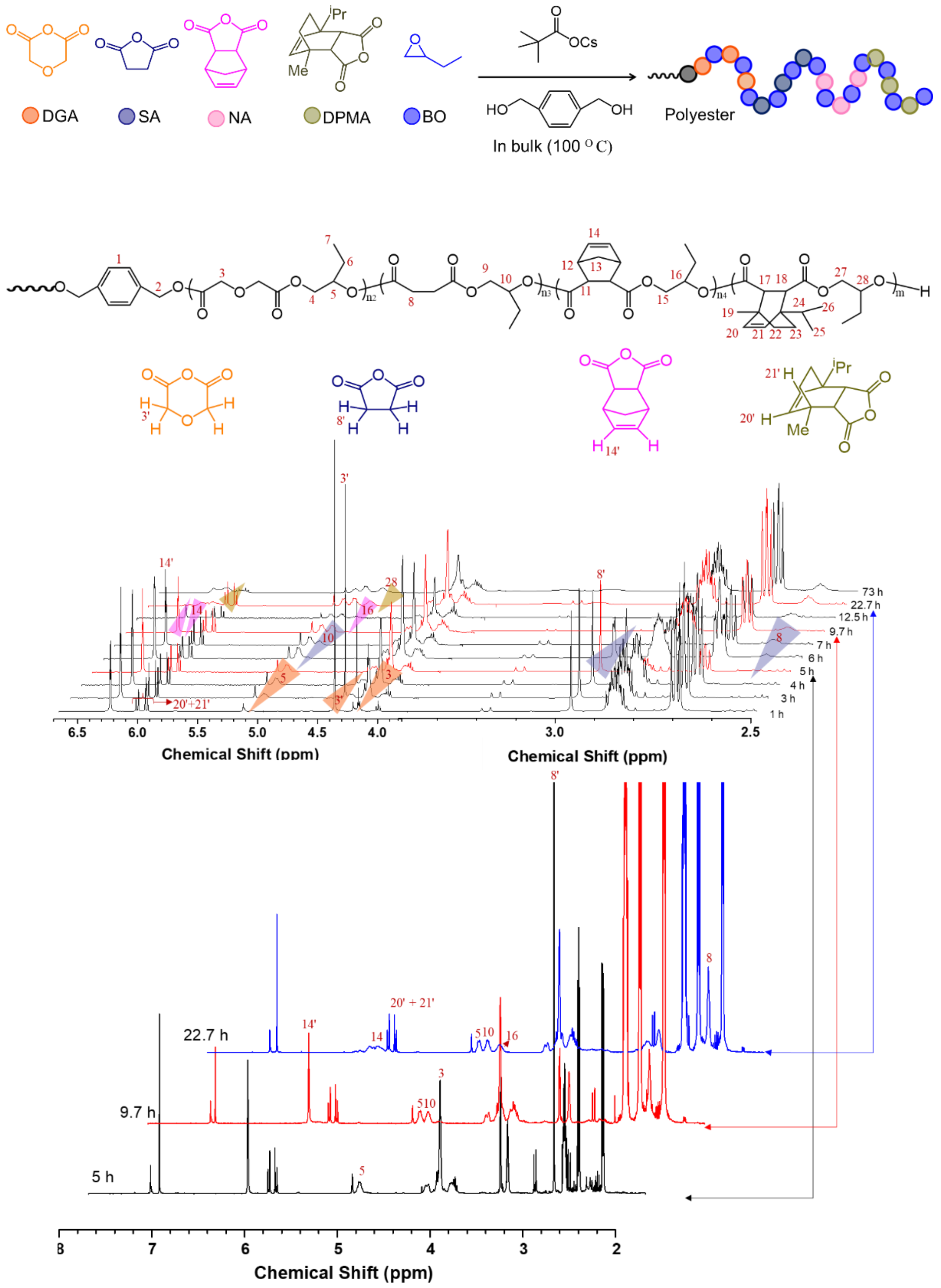

Supplementary Figure 16 | The ^{1}H NMR ($CDCl_3$) spectra of crude aliquots withdrawn from the reaction system for monitoring the conversion of DGA, SA, NA, DPMA, and the formation of resultant heptablock polymers (entry 2 in Table 1).

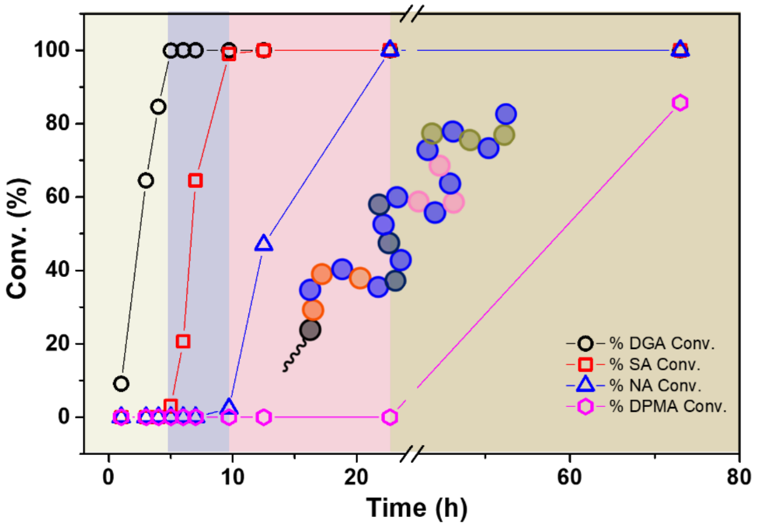

Supplementary Figure 17 | The plots of monomer conversion versus time. The polymerization of DGA, SA, NA, DPMA with BO was performed at 100 ℃ (entry 2 in Table 1).

Calculation method for degree of polymerization (DP) of each block:

$DP_{P(DGA\text{-}alt\text{-}BO)}$ = 12.5 × conv. of DGA = 12.5 per block

$DP_{P(SA\text{-}alt\text{-}BO)}$ = (12.5 × conv. of SA)/2 = 6.3 per block

$DP_{P(NA\text{-}alt\text{-}BO)}$ = (12.5 × conv. of NA)/2 = 6.3 per block

$DP_{P(DPMA\text{-}alt\text{-}BO)}$ = (6.25 × conv. of DPMA)/2 = 2.7 per block

P(DPMA-*alt*-BO)$_{2.7}$-*b*-P(NA-*alt*-BO)$_{6.3}$-*b*-P(SA-*alt*-BO)$_{6.3}$-*b*-P(DGA-*alt*-BO)$_{12.5}$-*b*-P(SA-*alt*-BO)$_{6.3}$-*b*-P(NA-*alt*-BO)$_{6.3}$-*b*-P(DPMA-*alt*-BO)$_{2.7}$

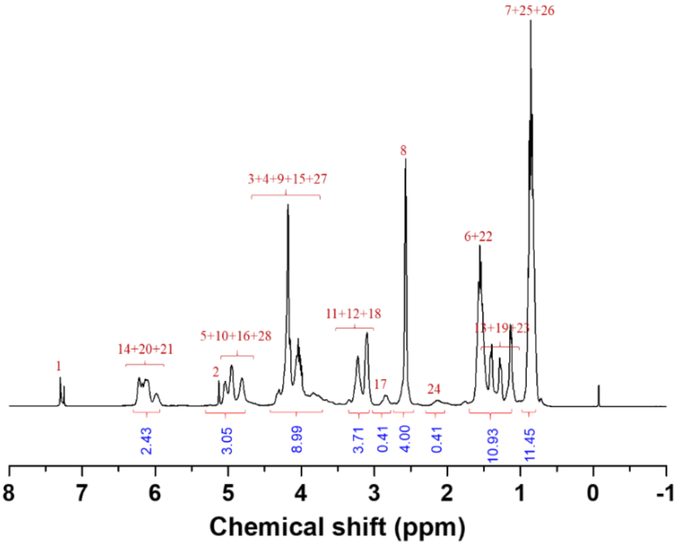

Supplementary Figure 18 | The ^{1}H NMR ($CDCl_3$) spectrum of the resultant heptablock polymer isolated from the mixture by precipitation (entry 2 in Table 1).

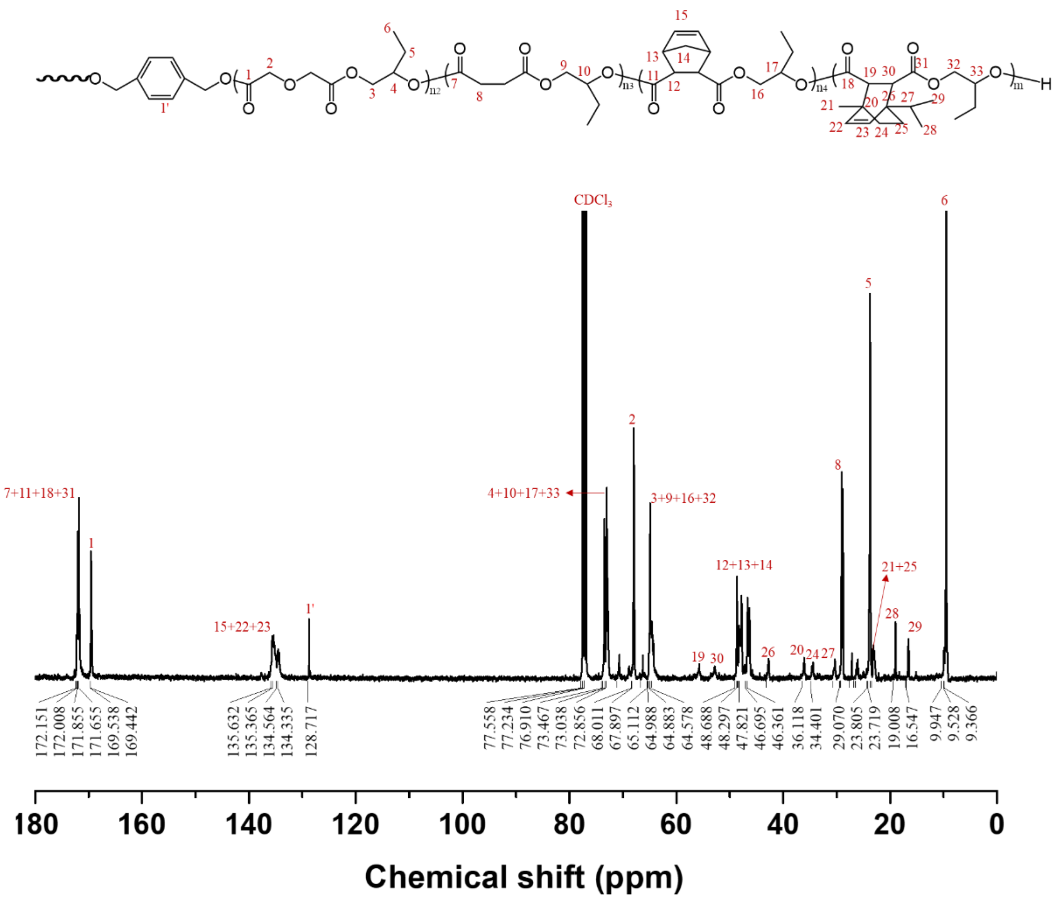

Supplementary Figure 19 | The ^{13}C NMR ($CDCl_3$) spectrum of the resultant heptablock polymer (entry 2 in Table 1). Chemical shifts from the carbonyl groups of P(SA-*alt*-epoxide), P(NA-*alt*-epoxide), and P(DPMA-*alt*-epoxide) locate at 172.5 ppm ~ 171.5 ppm.[4-6]

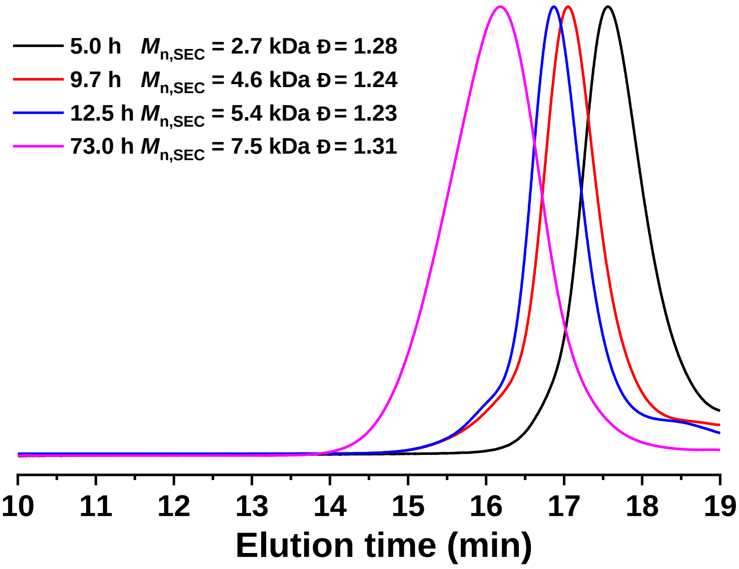

Supplementary Figure 20 | The SEC (THF) trace of the resultant heptablock polymers (entry 2 in Table 1).

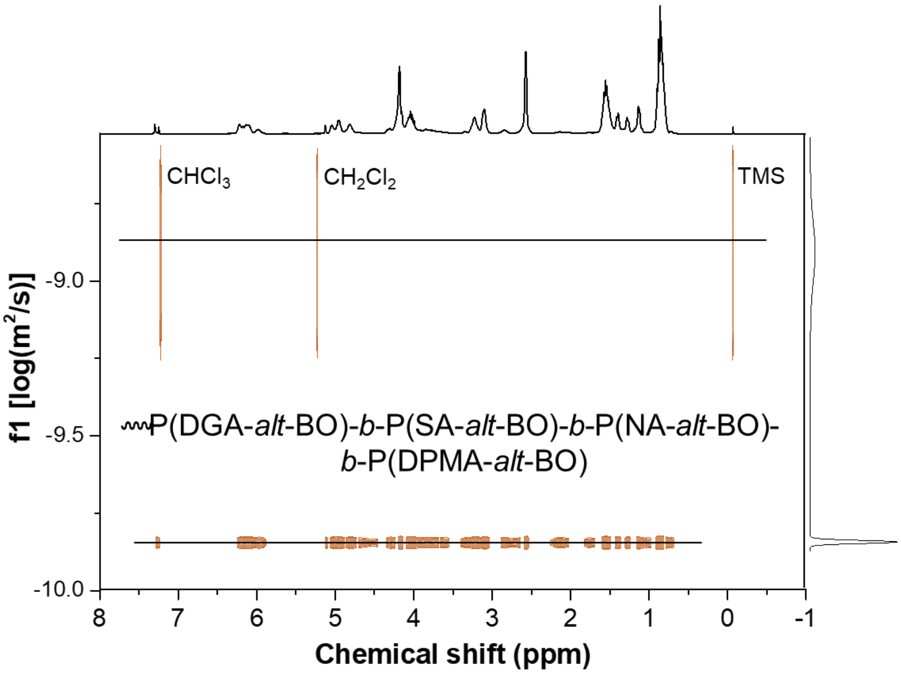

Supplementary Figure 21 | The DOSY NMR ($CDCl_3$) spectrum of the resultant heptablock polymer (entry 2 in Table 1).

The polymerization of DGA/SA/NA/EGE

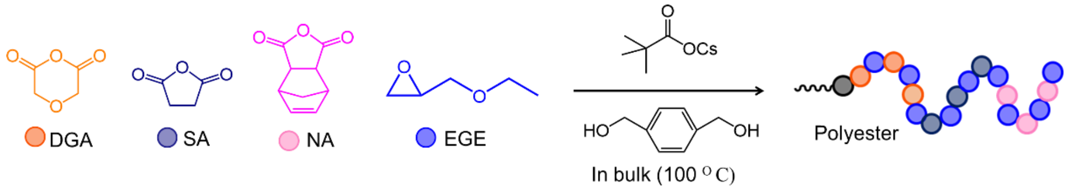

Supplementary Table 1 | ^{1}H NMR Peak Assignments for the resultant pentablock polymer (entry 3 in Table 1).

Group	1	2	3	4	5	6,7	8	9
^{1}H (δ ppm)	7.34	5.16	4.25–4.15	4.27–4.22	5.29–5.20	3.60–3.34	1.20–1.09	2.73–2.53
Group	10	11	12	13	14	15	16	17
^{1}H (δ ppm)	4.49–3.87	5.21–5.12	3.19–3.04	3.32–3.20	1.47–1.25	6.37–6.13	4.49–3.87	5.10–4.97

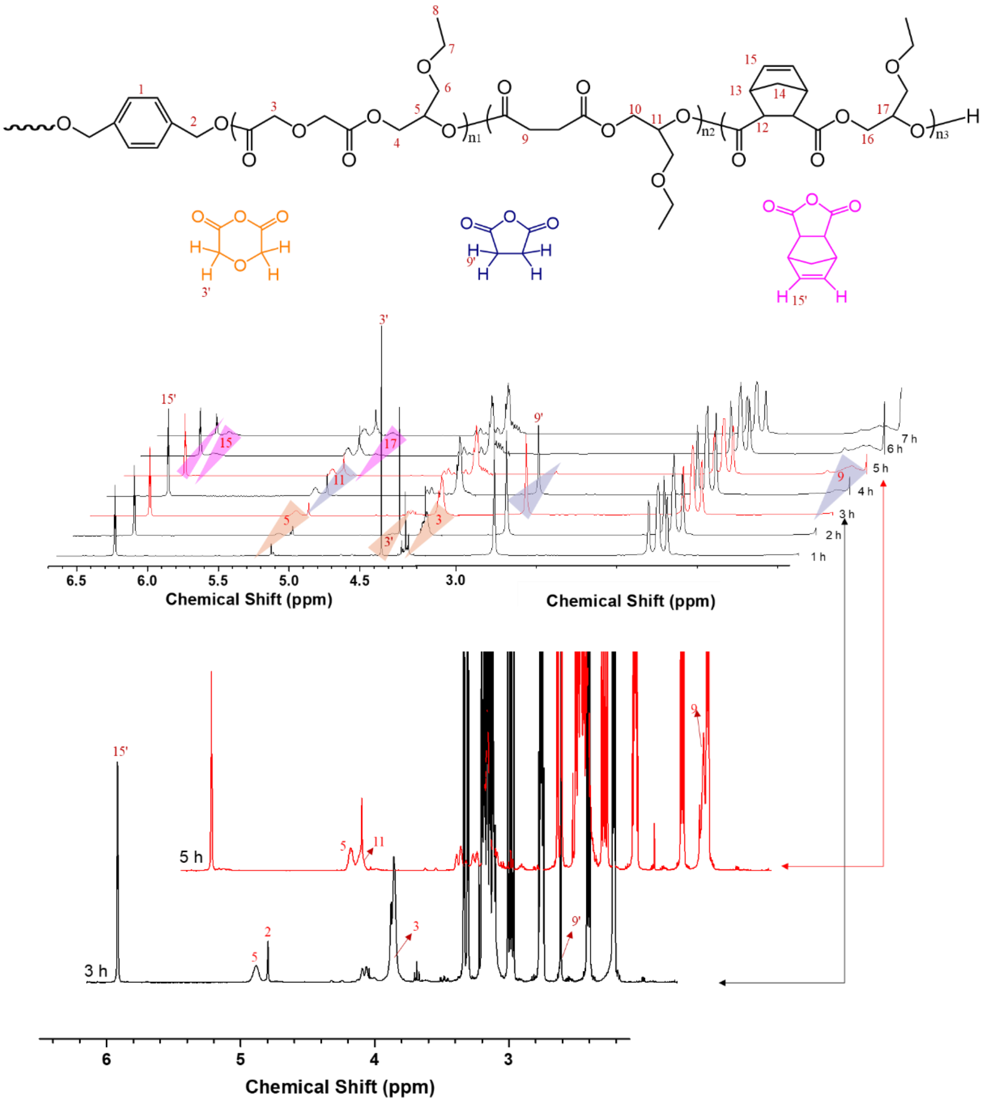

Supplementary Figure 22 | The ^{1}H NMR ($CDCl_3$) spectra of crude aliquots withdrawn from the reaction system for monitoring the conversion of DGA, SA, NA, and the formation of resultant polymers (entry 3 in Table 1).

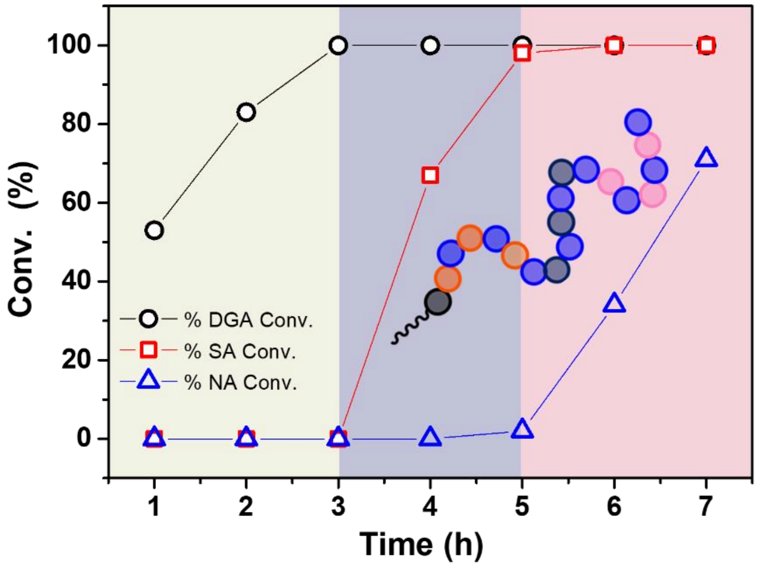

Supplementary Figure 23 | The plots of monomer conversion versus time. The polymerization of DGA, SA, NA, with EGE was performed at 100 ℃ (entry 3 in Table 1).

Calculation method for degree of polymerization (DP) of each block:

$DP_{P(DGA\text{-}alt\text{-}EGE)}$ = 12.5 × conv. of DGA = 12.5 per block

$DP_{P(SA\text{-}alt\text{-}EGE)}$ = (12.5 × conv. of SA)/2 = 6.3 per block

$DP_{P(NA\text{-}alt\text{-}EGE)}$ = (12.5 × conv. of NA)/2 = 4.5 per block

P(NA-*alt*-EGE)$_{4.5}$-*b*-P(SA-*alt*-EGE)$_{6.3}$-*b*-P(DGA-*alt*-EGE)$_{12.5}$-*b*-P(SA-*alt*-EGE)$_{6.3}$-*b*-P(NA-*alt*-EGE)$_{4.5}$

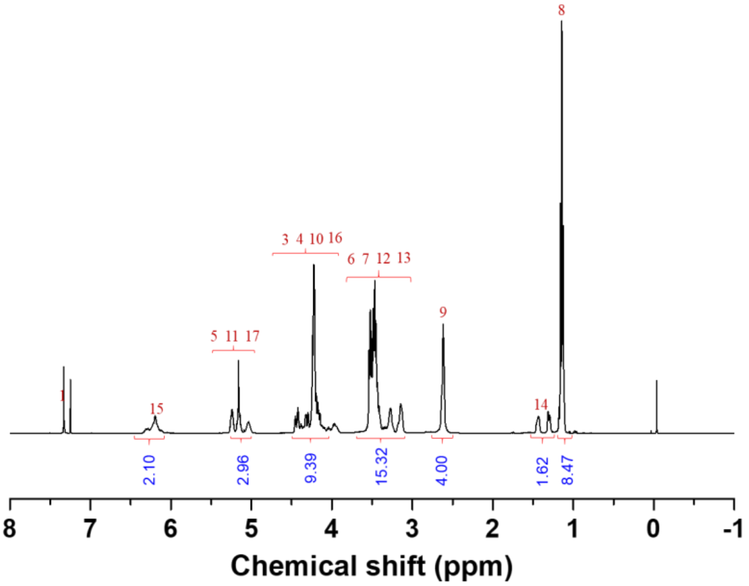

Supplementary Figure 24 | The ^{1}H NMR ($CDCl_3$) spectrum of the resultant pentablock polymer isolated from the mixture by precipitation (entry 3 in Table 1).

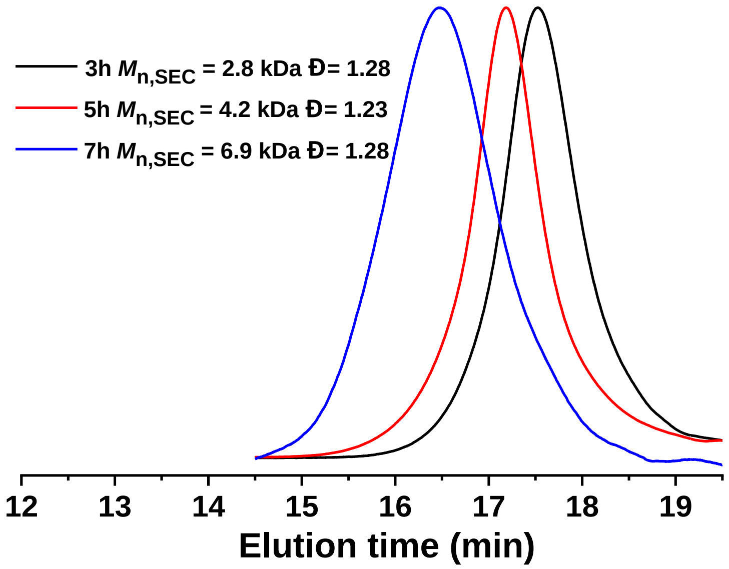

Supplementary Figure 25 | The SEC (THF) trace of the resultant pentablock polymers (entry 3 in Table 1).

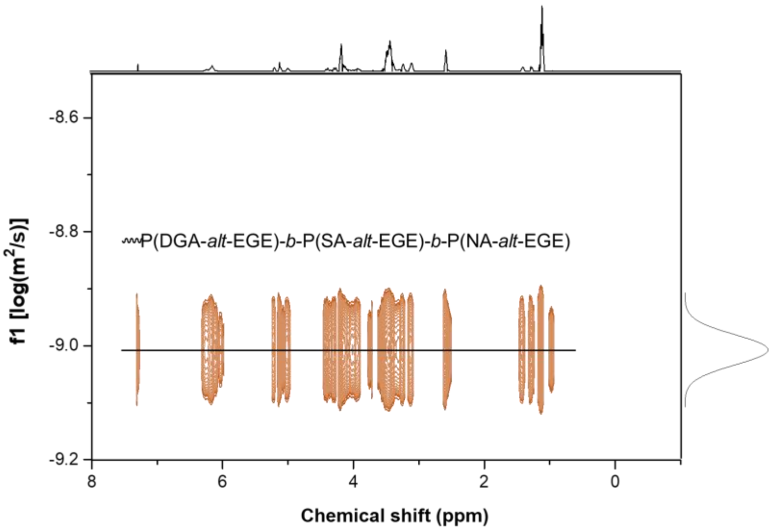

Supplementary Figure 26 | The DOSY NMR ($CDCl_3$) spectrum of the resultant pentablock polymer (entry 3 in Table 1).

Supplementary Table 2 | ^{13}C NMR Peak Assignments for the resultant pentablock polymer (entry 3 in Table 1).

Group	1	2	3	4	5, 12, 19	6, 13, 20	7	8
^{13}C (δ ppm)	128.1	n.d.	169.5	67.9	63.3–62.9	68.5–68.0	70.9	67.1
Group	9	10	11	14	15	16	17	18
^{13}C (δ ppm)	15.0	171.6	28.8	171.9	46.6	47.6	46.6	135.3

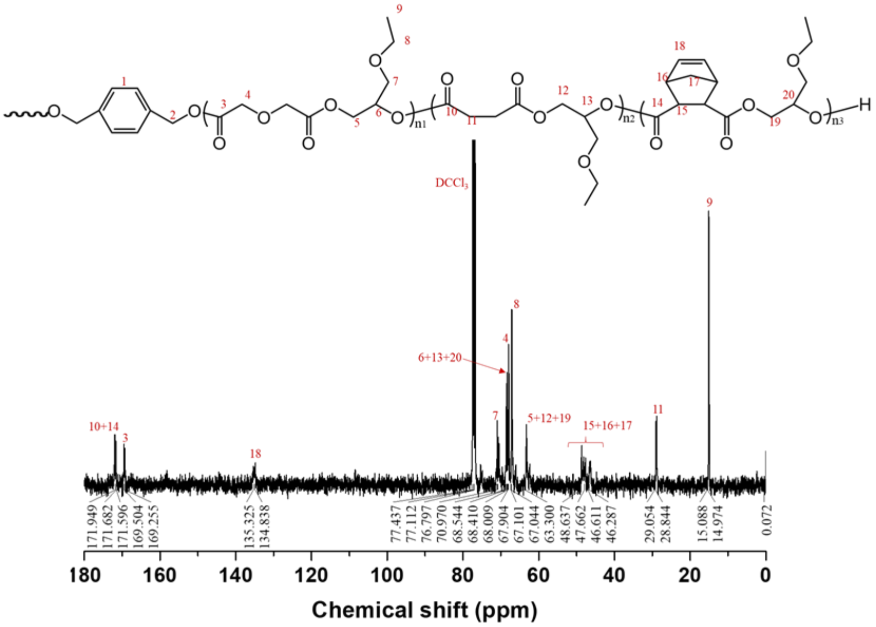

Supplementary Figure 27 | The ^{13}C NMR ($CDCl_3$) spectrum of the resultant pentablock polymer (entry 3 in Table 1). Chemical shifts from the carbonyl groups of P(SA-*alt*-epoxide) and P(NA-*alt*-epoxide) locate at 172.5 ppm ~ 171.5 ppm.[4-6]

The terpolymerization of L-LA/DPMA/EGE

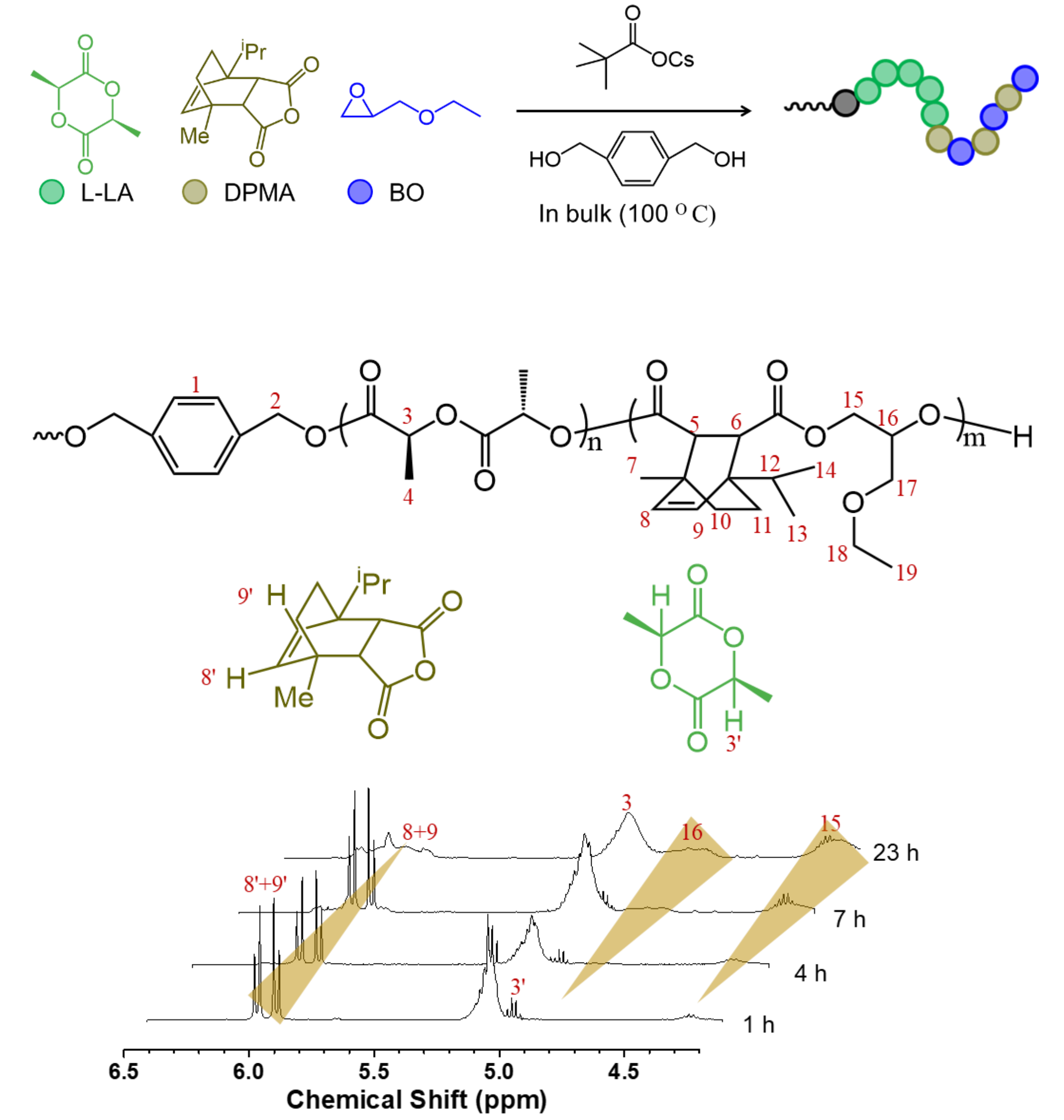

Supplementary Figure 28 | The ^{1}H NMR ($CDCl_3$) spectra of crude aliquots withdrawn from the reaction system for monitoring the conversion of DPMA, L-LA, and the formation of resultant polymers (entry 4 in Table 1).

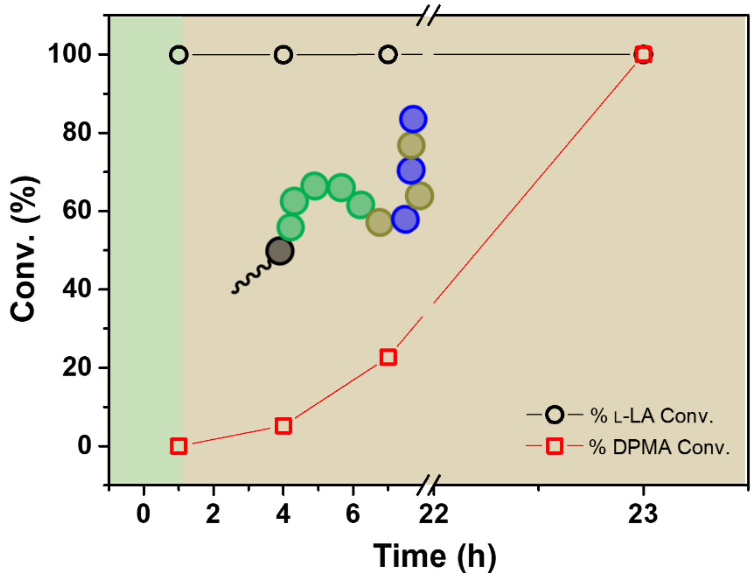

Supplementary Figure 29 | The plots of monomer conversion versus time. The polymerization of DPMA, L-LA with EGE was performed at 100 ℃ (entry 4 in Table 1).

Calculation method for degree of polymerization (DP) of each block:

DP_{PLLA} = 25 × conv. of L-LA = 25 per block

$DP_{P(DPMA\text{-}alt\text{-}EGE)}$ = (12.5 × conv. of DPMA)/2 = 5 per block

P(DPMA-*alt*-EGE)$_5$-*b*-PLLA$_{25}$-*b*-P(DPMA-*alt*-EGE)$_5$

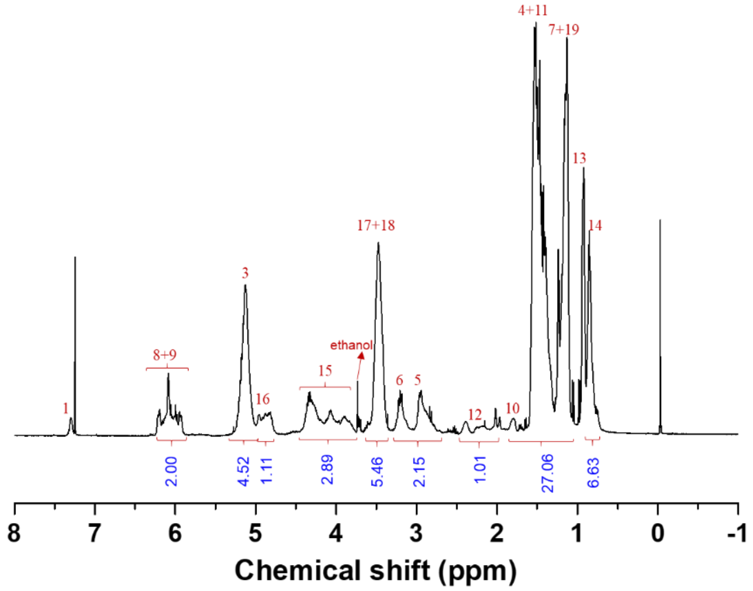

Supplementary Figure 30 | The ^1H NMR ($CDCl_3$) spectrum of the resultant polymer isolated from the mixture by precipitation (entry 4 in Table 1).

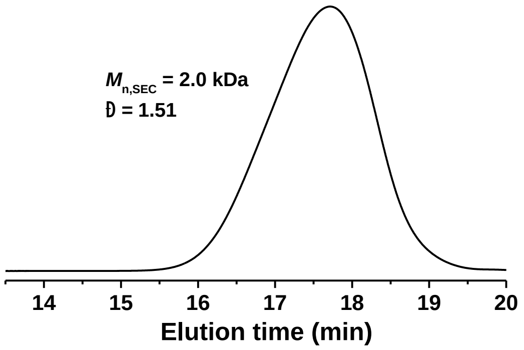

Supplementary Figure 31 | The SEC (THF) trace of the resultant triblock polymers (entry 4 in Table 1).

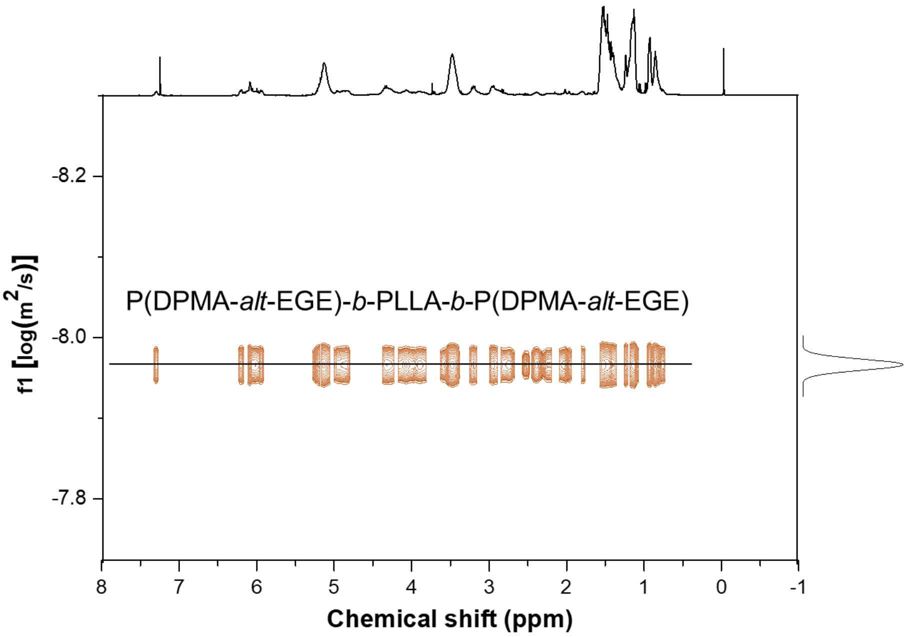

Supplementary Figure 32 | The DOSY NMR ($CDCl_3$) spectrum of the resultant triblock polymer (entry 4 in Table 1).

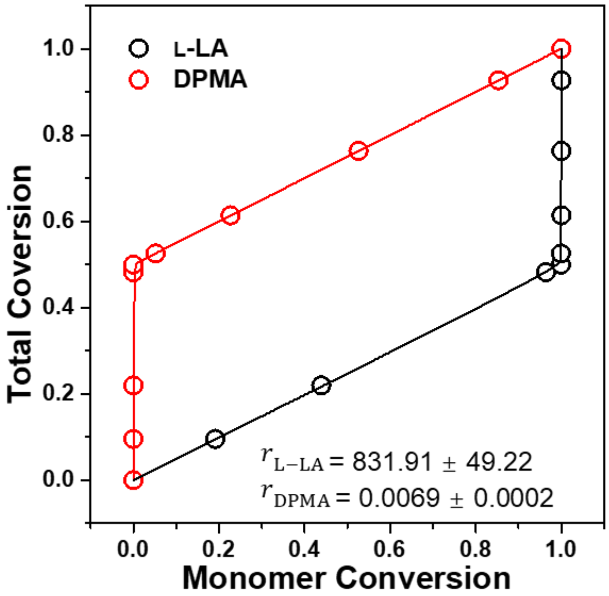

Supplementary Figure 33 | Total polymerization conversion plotted against monomer conversion the data were obtained from time-resolved ^{1}H NMR spectra of a cesium pivalate-catalyzed copolymerization of DPMA/L-LA/EGE with a molar ratio of 25/50/250 at 100 °C. Solid black and red lines represent fits to the experimental data using the nonterminal model, eqs 1 and 2.

ROCOP of DPMA/BO

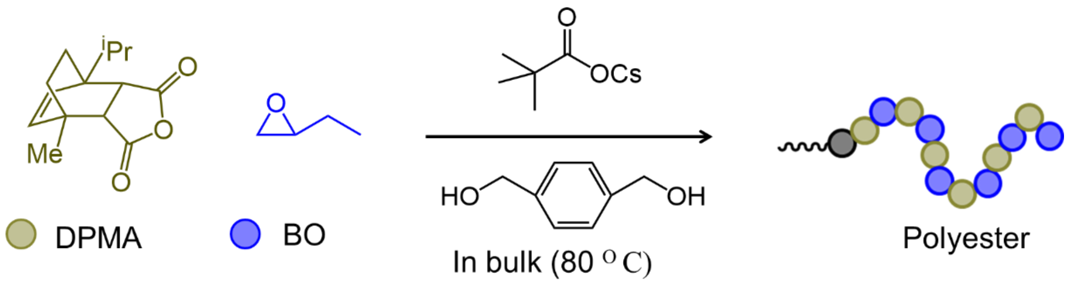

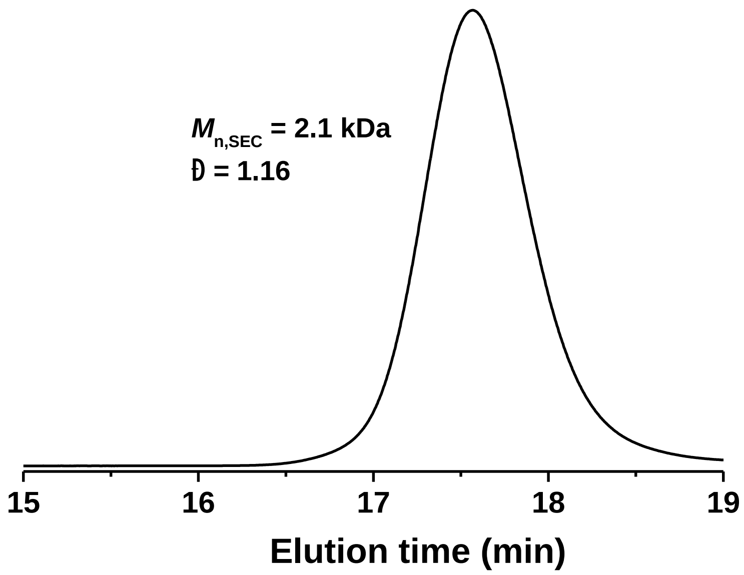

Supplementary Figure 34 | The SEC (THF) trace of the resultant polymers (entry 5 in Table 1).

Calculation method for degree of polymerization (DP) of each block:

$DP_{P(DPMA\text{-}alt\text{-}BO)}$ = 12.5 × conv. of DPMA = 12.5 per block

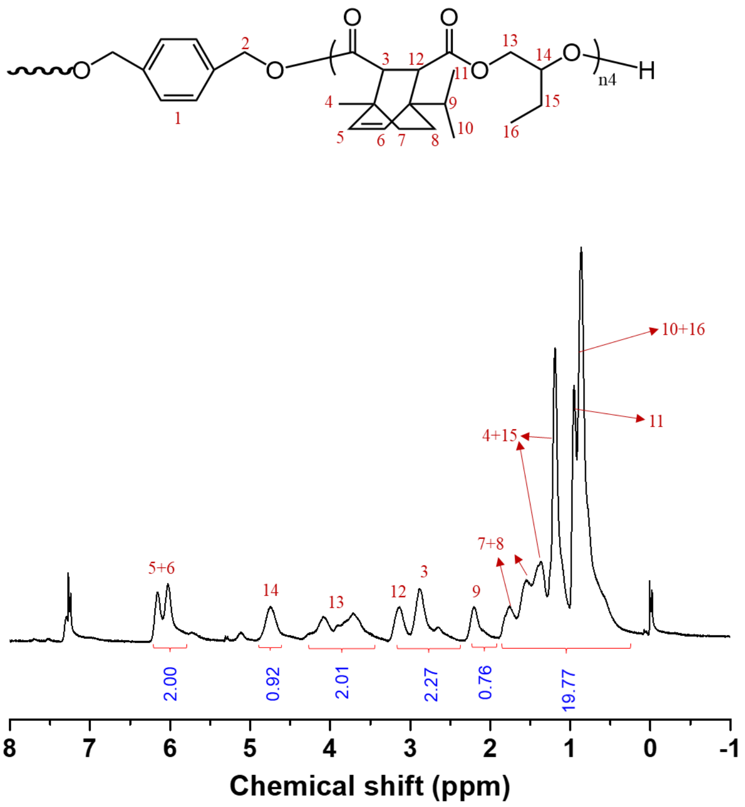

Supplementary Figure 35 | The ^{1}H NMR ($CDCl_3$) spectrum of the resultant polymer isolated from the mixture by precipitation (entry 5 in Table 1).

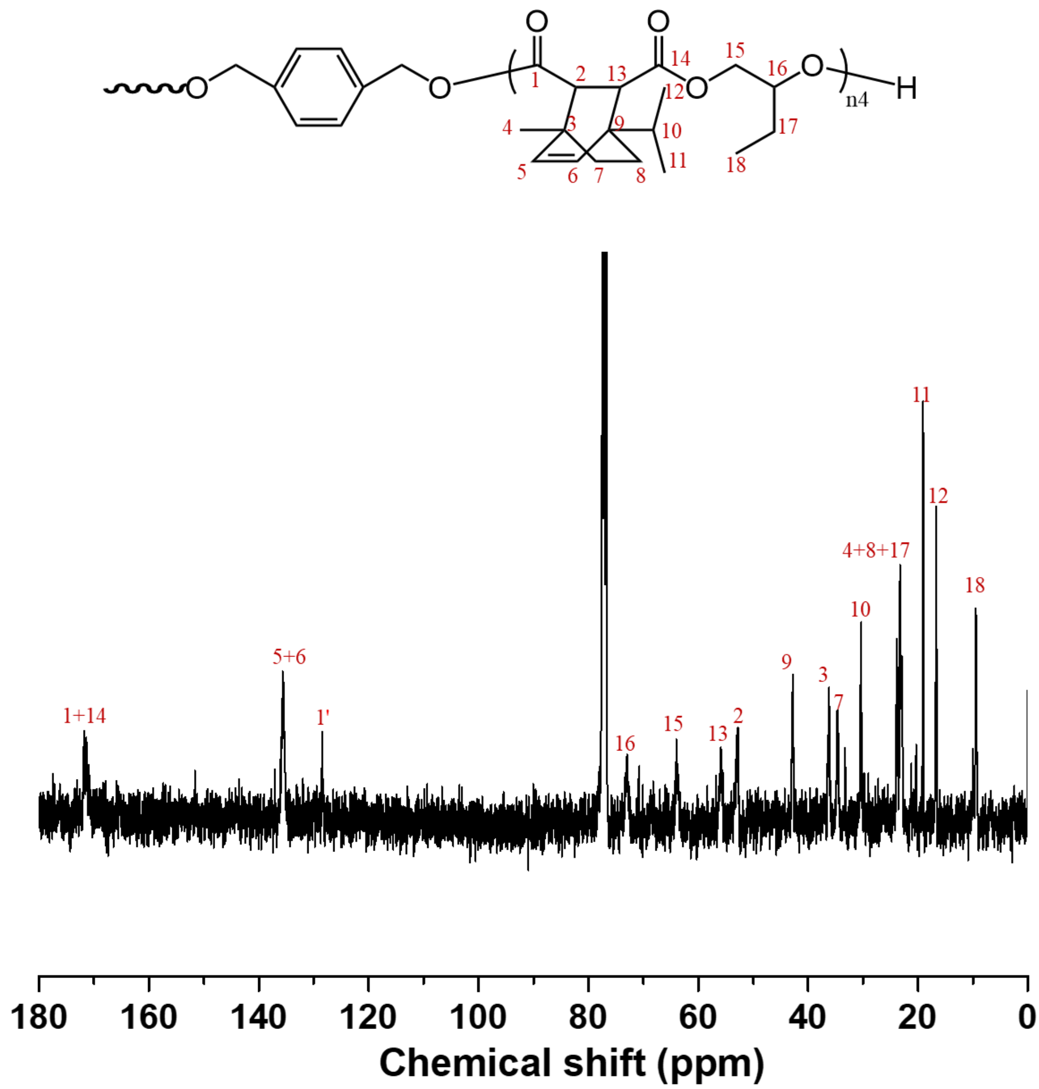

Supplementary Figure 36 | The ^{13}C NMR ($CDCl_3$) spectrum of the resultant polymer isolated from the mixture by precipitation (entry 5 in Table 1).

The terpolymerization of L-LA/DPMA/BO

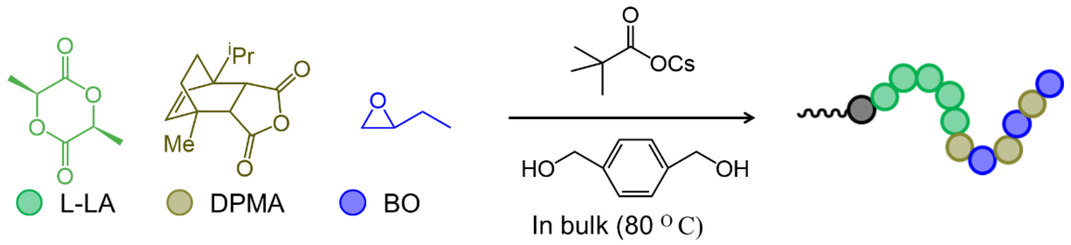

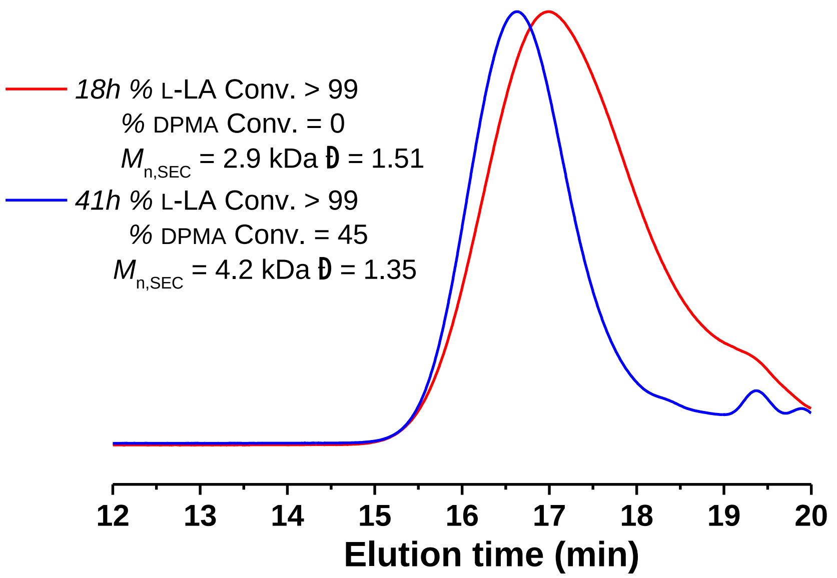

Supplementary Figure 37 | The SEC (THF) trace of the resultant triblock polymers (entry 6 in Table 1).

Calculation method for degree of polymerization (DP) of each block:

DP_{PLLA} = 25 × conv. of L-LA = 25 per block

$DP_{P(DPMA\text{-}alt\text{-}BO)}$ = (12.5 × conv. of DPMA)/2 = 2.8 per block

P(DPMA-*alt*-BO)$_{2.8}$-*b*-PLLA$_{25}$-*b*-P(DPMA-*alt*-BO)$_{2.8}$

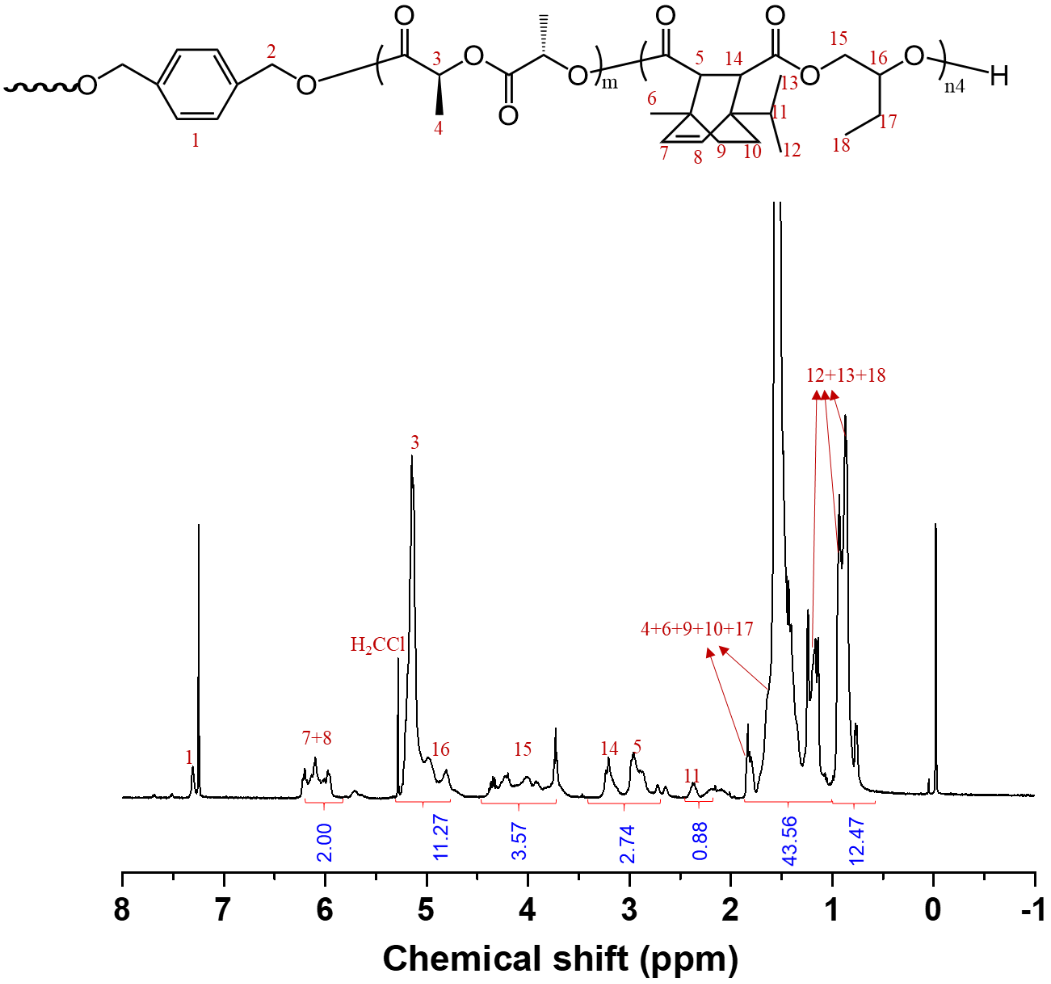

Supplementary Figure 38 | The ^{1}H NMR ($CDCl_3$) spectrum of the resultant polymer isolated from the mixture by precipitation (entry 6 in Table 1).

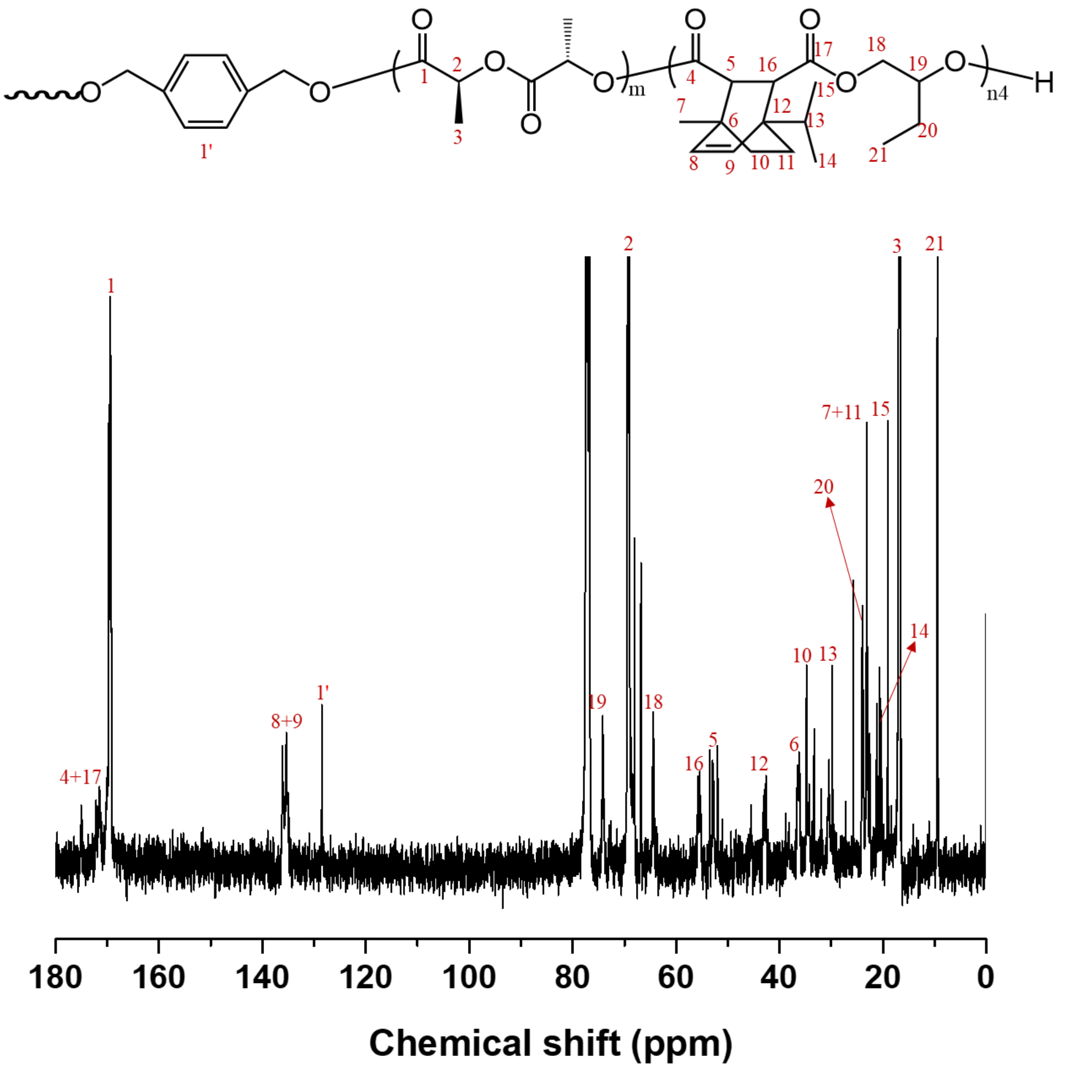

Supplementary Figure 39 | The ^{13}C NMR ($CDCl_3$) spectrum of the resultant polymer isolated from the mixture by precipitation (entry 6 in Table 1).

The polymerization of DGA/SA/NA/L-LA/DPMA/BO

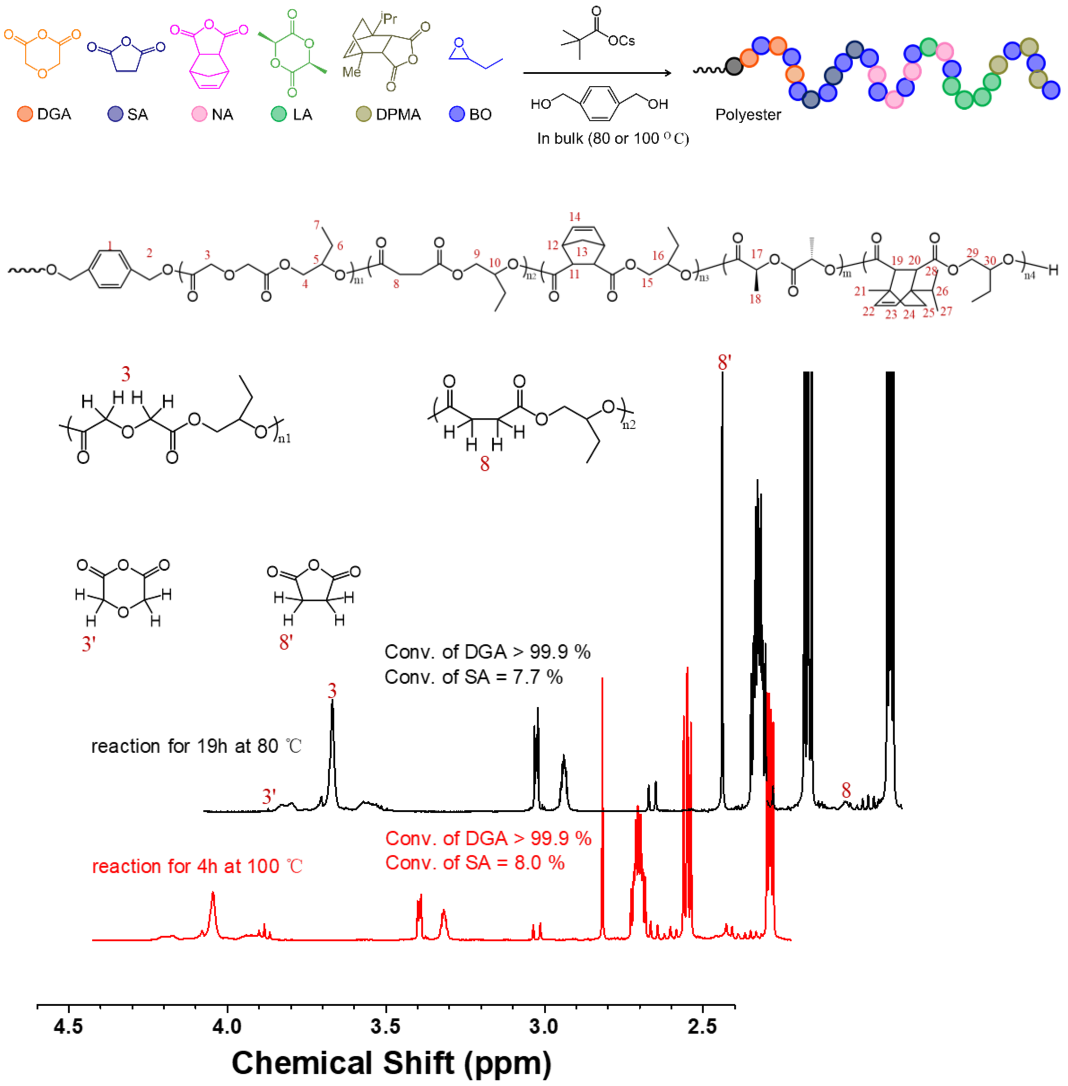

Supplementary Figure 40 | The ^{1}H NMR ($CDCl_3$) spectra of crude aliquots withdrawn from the reaction system for monitoring the conversion of DGA, SA, NA, DPMA, L-LA, and the formation of resultant polymers. Red line (entry7 in Table 1) and black line (entry 8 in Table 1).

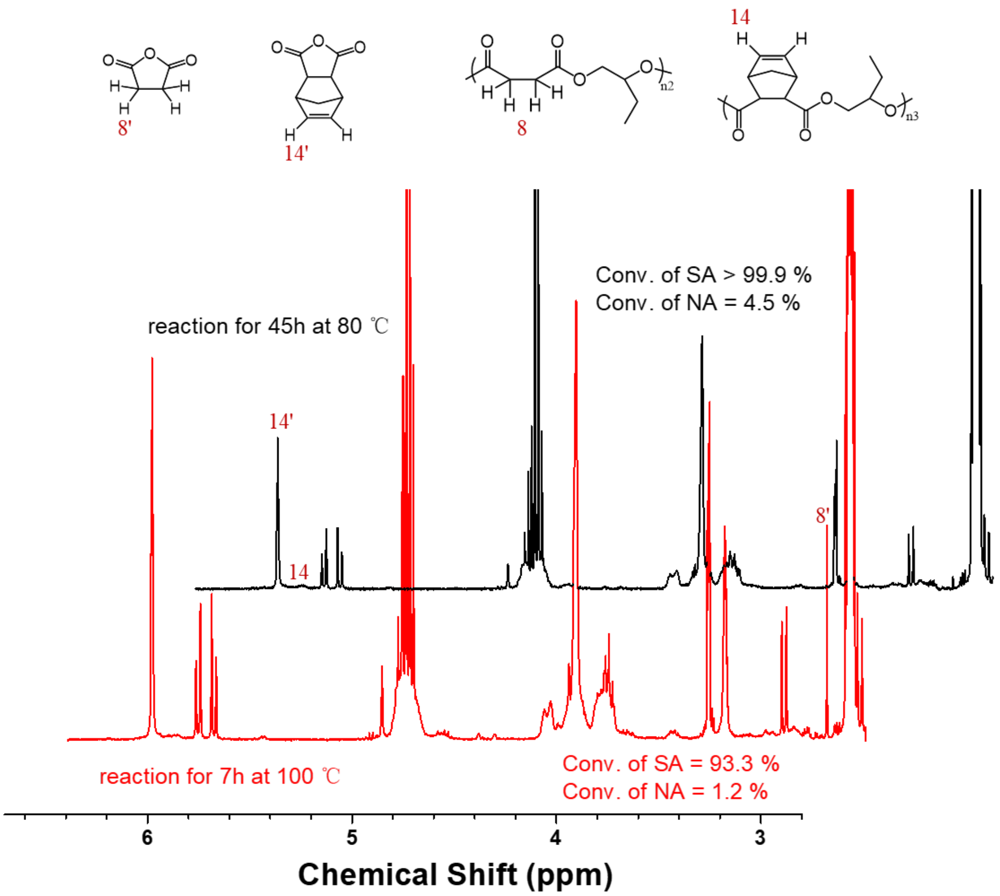

Supplementary Figure 41 | The ^{1}H NMR ($CDCl_3$) spectra of crude aliquots withdrawn from the reaction system for monitoring the conversion of DGA, SA, NA, DPMA, L-LA, and the formation of resultant polymers. Red line (entry7 in Table 1) and black line (entry 8 in Table 1).

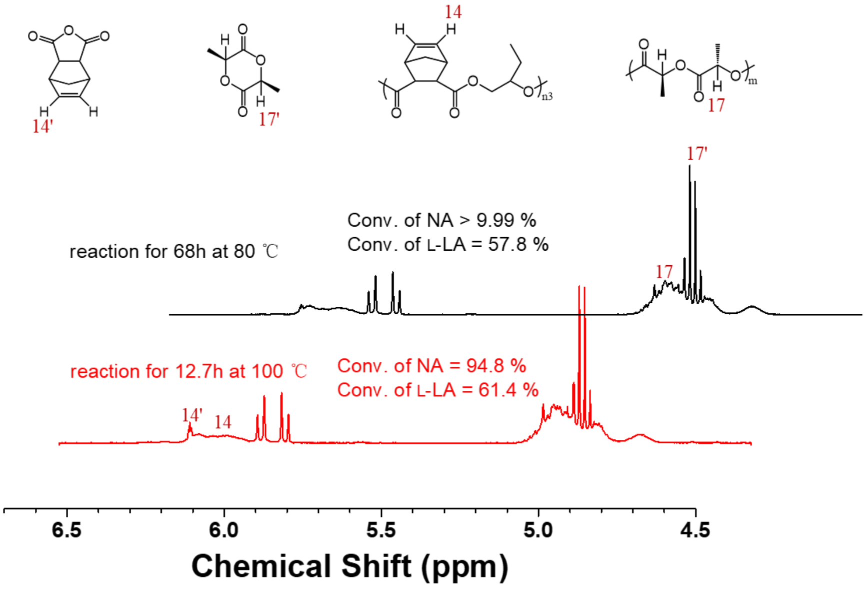

Supplementary Figure 42 | The ^{1}H NMR ($CDCl_3$) spectra of crude aliquots withdrawn from the reaction system for monitoring the conversion of DGA, SA, NA, DPMA, L-LA, and the formation of resultant polymers. Red line (entry 7 in Table 1) and black line (entry 8 in Table 1).

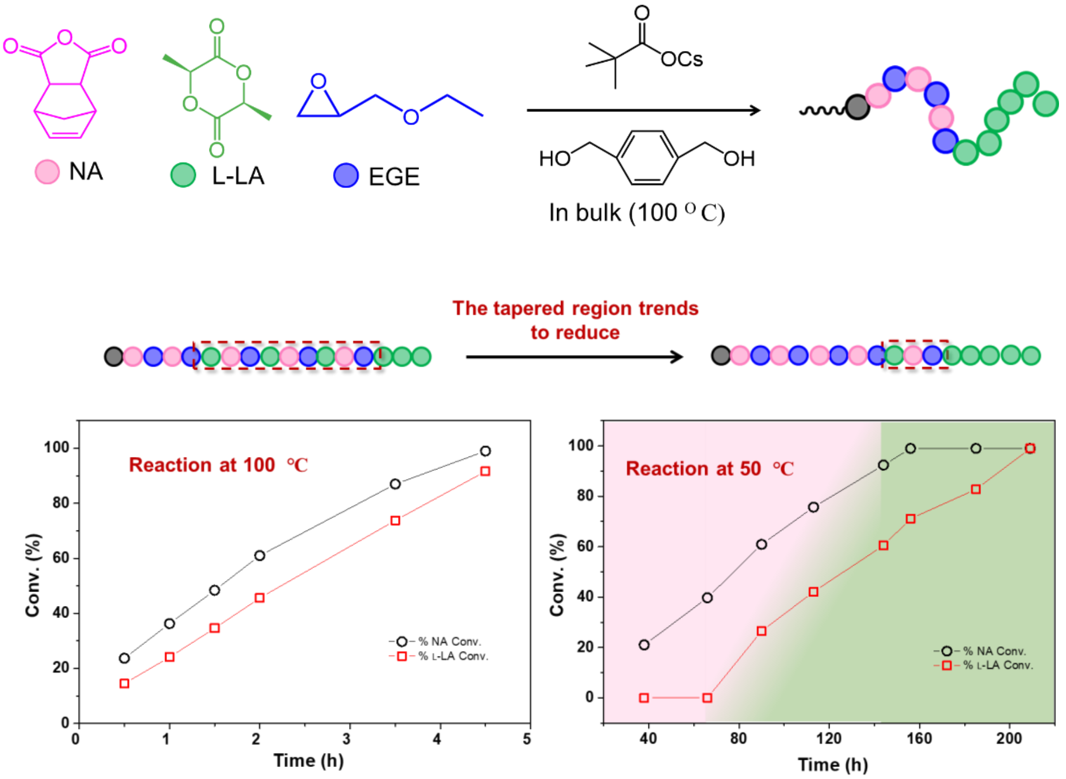

Supplementary Figure 43 | The plots of monomer conversion versus time. The polymerization of NA, L-LA with EGE was performed at 50 and 100 ℃, respectively.

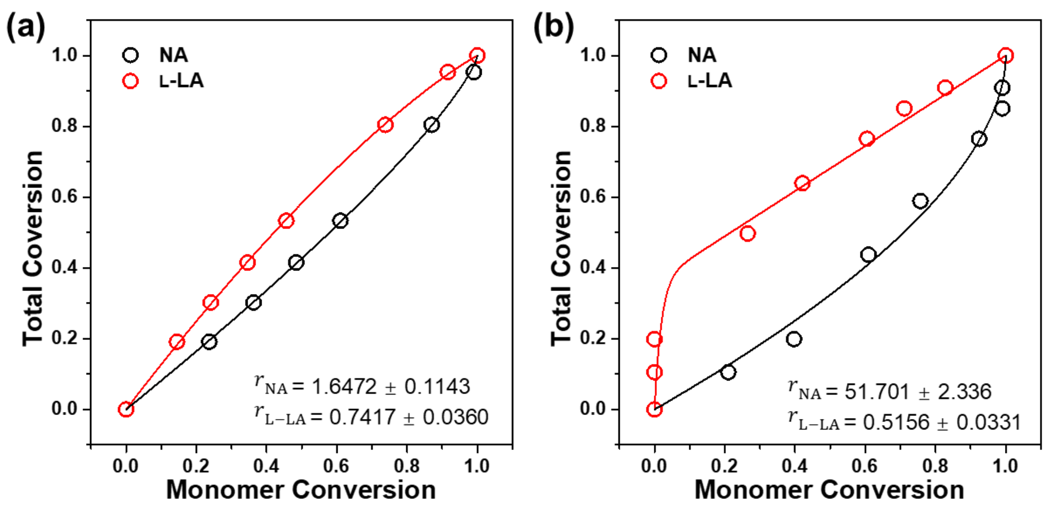

Supplementary Figure 44 | Total polymerization conversion plotted against monomer conversion. Solid black and red lines represent fits to the experimental data using the nonterminal model, eqs 1 and 2. (a) copolymerization of NA/EGE/L-LA with a molar ratio of 25/50/250 at 100 ℃; (b) copolymerization of NA/EGE/L-LA with a molar ratio of 25/50/250 at 50 ℃.

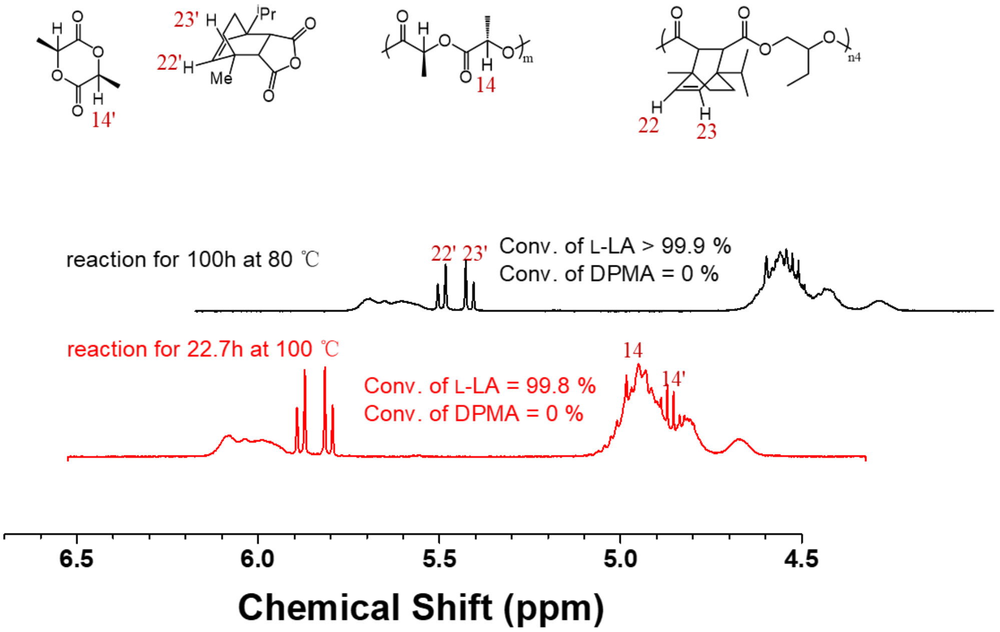

Supplementary Figure 45 | The ^{1}H NMR ($CDCl_3$) spectra of crude aliquots withdrawn from the reaction system for monitoring the conversion of DGA, SA, NA, DPMA, L-LA, and the formation of resultant polymers. Red line (entry 7 in Table 1) and black line (entry 8 in Table 1)

Calculation method for degree of polymerization (DP) of each block (reaction at 100 ℃)

$DP_{P(DGA\text{-}alt\text{-}BO)}$ = 12.5 × conv. of DGA = 12.5 per block

$DP_{P(SA\text{-}alt\text{-}BO)}$ = (12.5 × conv. of SA)/2 = 6.3 per block

$DP_{P(NA\text{-}alt\text{-}BO)}$ = (12.5 × conv. of NA)/2 × 0.287 = 1.8 per block

DP_{PLLA} = (37.5 × conv. of L-LA)/2 × 0.344 = 6.5 per block

$DP_{P(NA\text{-}alt\text{-}BO\text{-}grad\text{-}L\text{-}LA)}$ = (12.5 × conv. of NA)/2 × 0.713 + (37.5 × conv. of L-LA)/2 × 0.656 = 16.8 per block

$DP_{P(DPMA\text{-}alt\text{-}BO)}$ = (6.25 × conv. of DPMA)/2 = 2.7 per block

P(DPMA-*alt*-BO)$_{2.7}$-*b*-PLLA$_{6.5}$-*b*-P(NA-*alt*-BO-*grad*-L-LA)$_{16.8}$-*b*-P(NA-*alt*-BO)$_{1.8}$-*b*-P(SA-*alt*-BO)$_{6.3}$-*b*-P(DGA-*alt*-BO)$_{12.5}$-*b*-P(SA-*alt*-BO)$_{6.3}$-*b*-P(NA-*alt*-BO)$_{1.8}$-*b*-P(NA-*alt*-BO-*grad*-L-LA)$_{16.8}$-*b*-PLLA$_{6.5}$-*b*-P(DPMA-*alt*-BO)$_{2.7}$

Calculation method for degree of polymerization (DP) of each block (reaction at 80 ℃)

$DP_{P(DGA\text{-}alt\text{-}BO)}$ = 12.5 × conv. of DGA = 12.5 per block

$DP_{P(SA\text{-}alt\text{-}BO)}$ = (12.5 × conv. of SA)/2 = 6.3 per block

$DP_{P(NA\text{-}alt\text{-}BO)}$ = (12.5 × conv. of NA)/2 × 0.439 = 2.7 per block

DP_{PLLA} = (37.5 × conv. of L-LA)/2 × 0.422 = 7.9 per block

$DP_{P(NA\text{-}alt\text{-}BO\text{-}grad\text{-}L\text{-}LA)}$ = (12.5 × conv. of NA)/2 × 0. 561 + (37.5 × conv. of L-LA)/2 × 0.578 = 14.3 per block

$DP_{P(DPMA\text{-}alt\text{-}BO)}$ = (6.25 × conv. of DPMA)/2 = 2.4 per block

P(DPMA-*alt*-BO)$_{2.4}$-*b*-PLLA$_{7.9}$-*b*-P(NA-*alt*-BO-*grad*-L-LA)$_{14.3}$-*b*-P(NA-*alt*-BO)$_{2.7}$-*b*-P(SA-*alt*-BO)$_{6.3}$-*b*-P(DGA-*alt*-BO)$_{12.5}$-*b*-P(SA-*alt*-BO)$_{6.3}$-*b*-P(NA-*alt*-BO)$_{2.7}$-*b*-P(NA-*alt*-BO-*grad*-L-LA)$_{14.3}$-*b*-PLLA$_{7.9}$-*b*-P(DPMA-*alt*-BO)$_{2.4}$

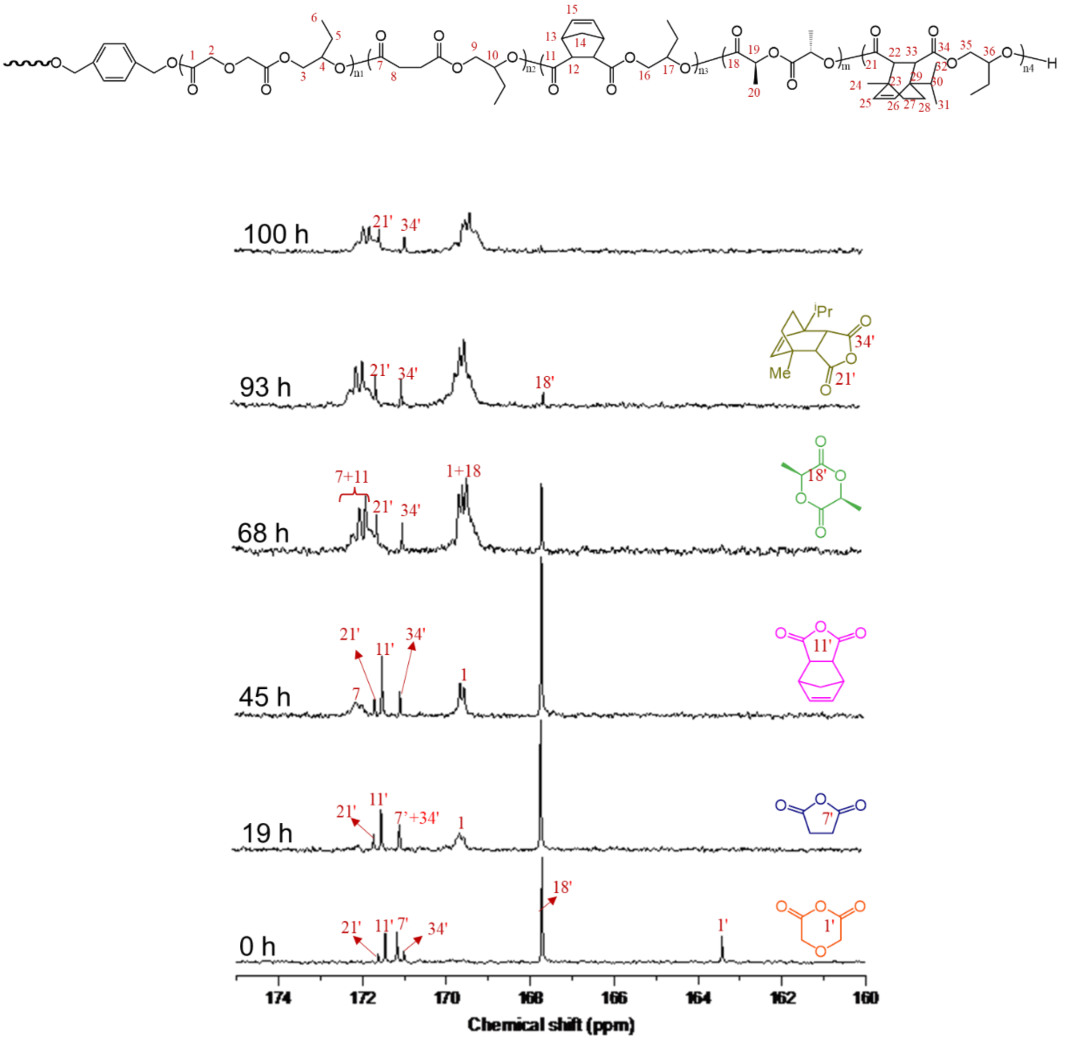

Supplementary Figure 46 | The ^{13}C NMR ($CDCl_3$) spectra of crude aliquots withdrawn from the reaction system for monitoring the conversion of DGA, SA, NA, DPMA, L-LA, and the formation of resultant polymers (entry 8 in Table 1).

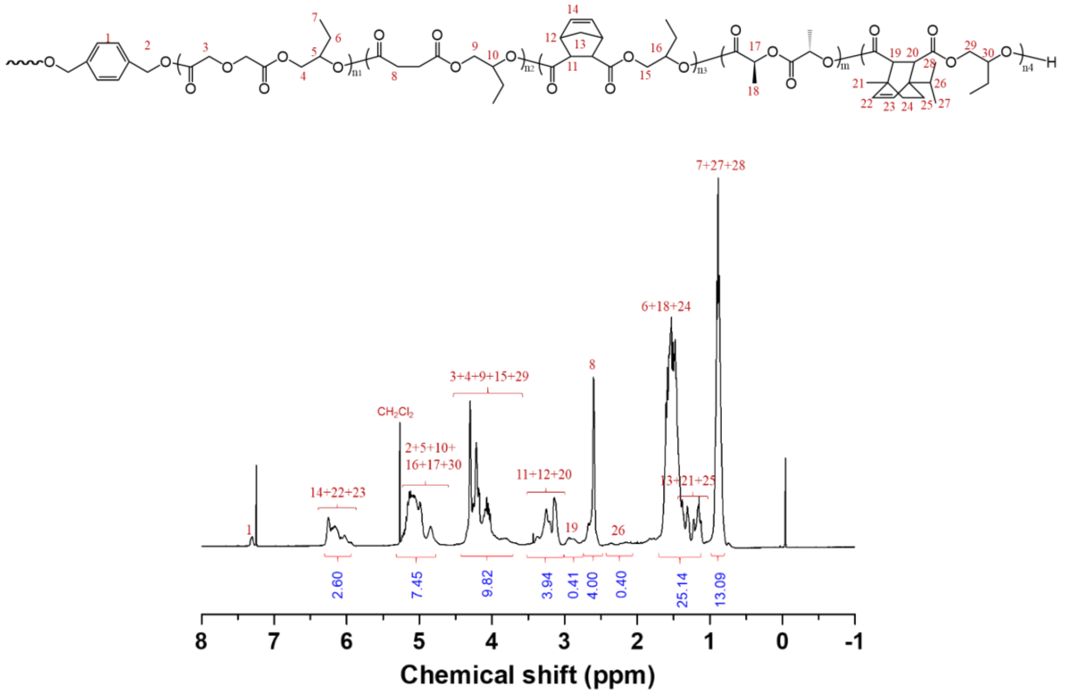

Supplementary Figure 47 | The ¹H NMR ($CDCl_3$) spectrum of the resultant enneablock polymer isolated from the mixture by precipitation (entry 8 in Table 1).

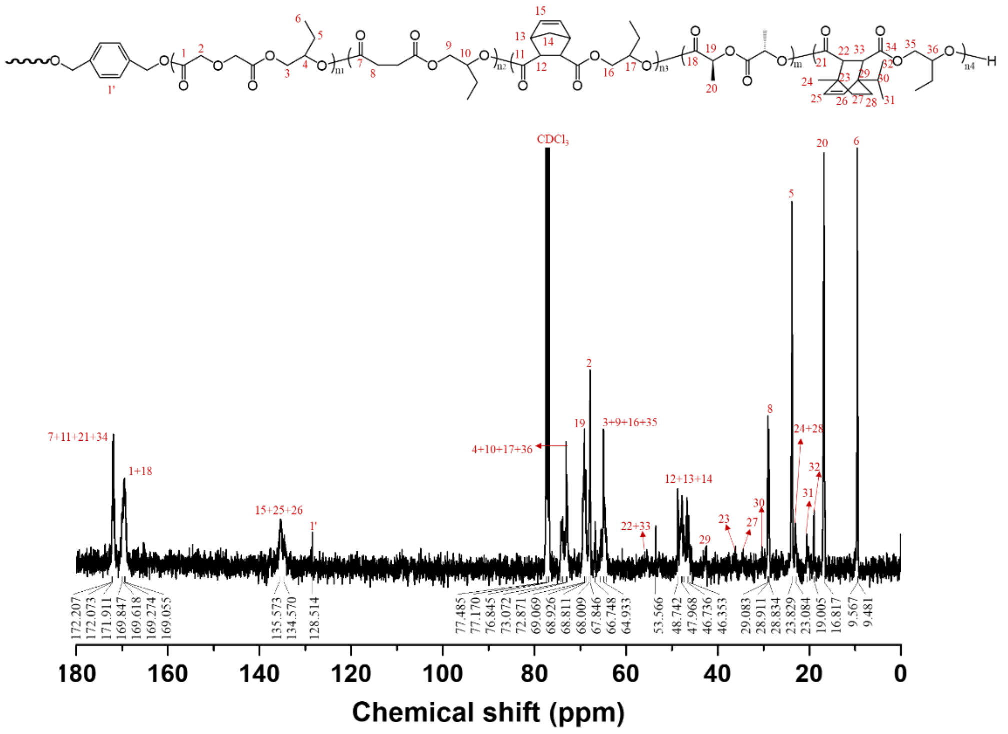

Supplementary Figure 48 | The ¹³C NMR ($CDCl_3$) spectrum of the resultant polymer isolated from the mixture by precipitation (entry 8 in Table 1). Chemical shifts from the carbonyl groups of P(SA-*alt*-epoxide), P(NA-*alt*-epoxide), and P(DPMA-*alt*-epoxide) locate at 172.5 ppm ~ 171.5 ppm, and Chemical shifts from the carbonyl groups of P(DGA-*alt*-epoxide) and PLLA locate at 169.1 ppm ~ 170.0 ppm.[4-6]

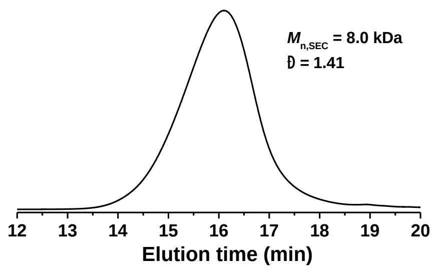

Supplementary Figure 49 | The SEC (THF) trace of the resultant enneablock polymers (entry 8 in Table 1).

The polymerization of DGA/SA/L-LA/DPMA/BO

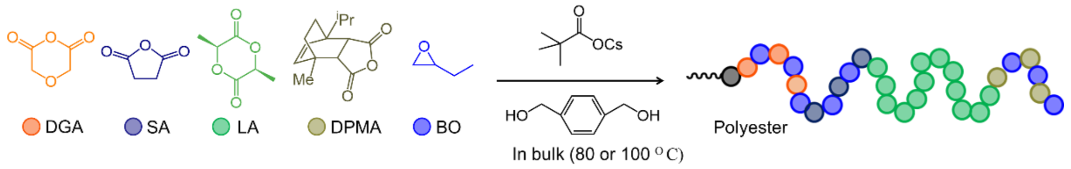

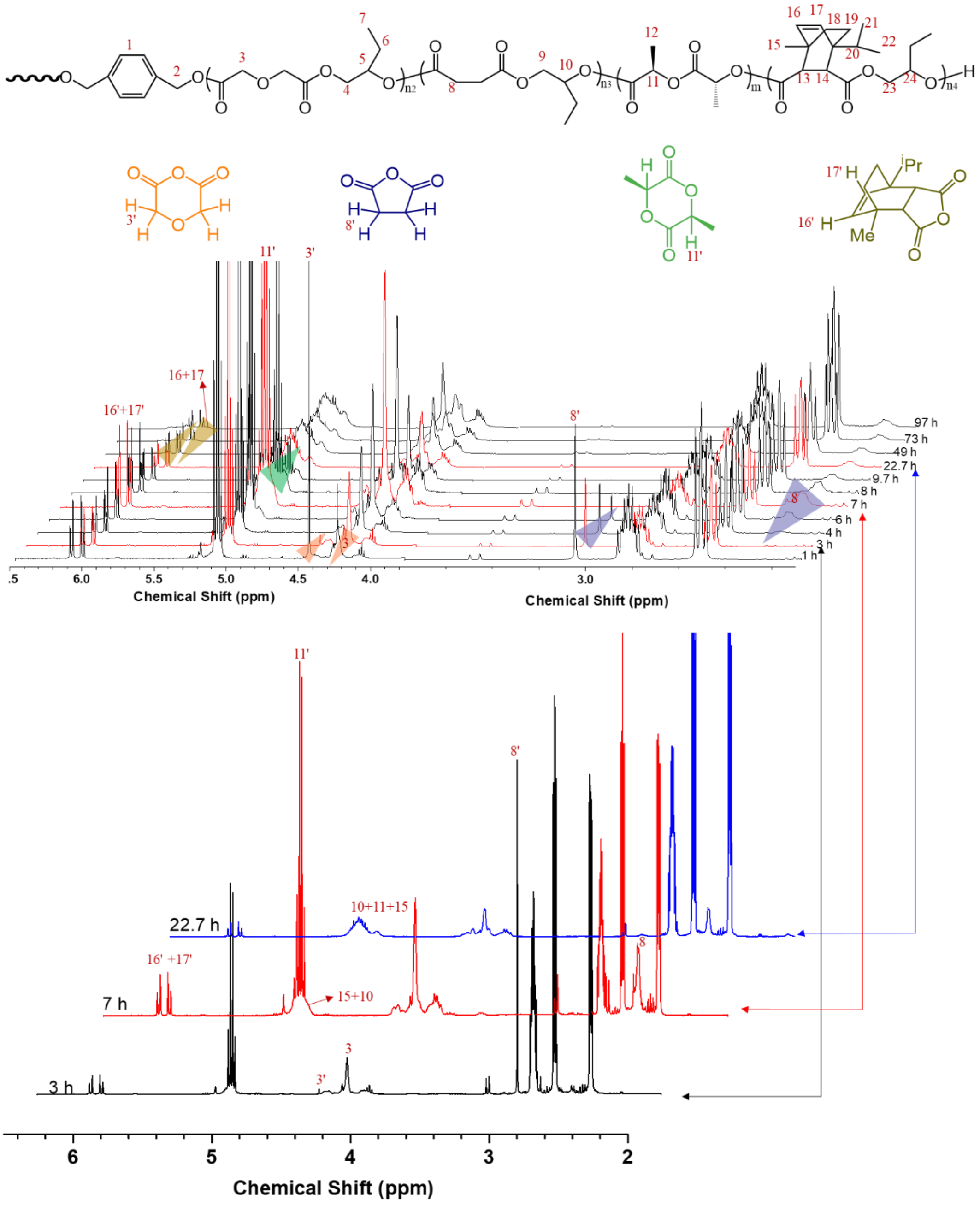

Supplementary Figure 50 | The ^{1}H NMR ($CDCl_3$) spectra of crude aliquots withdrawn from the reaction system for monitoring the conversion of DGA, SA, DPMA, L-LA, and the formation of resultant polymers (entry 9 in Table 1).

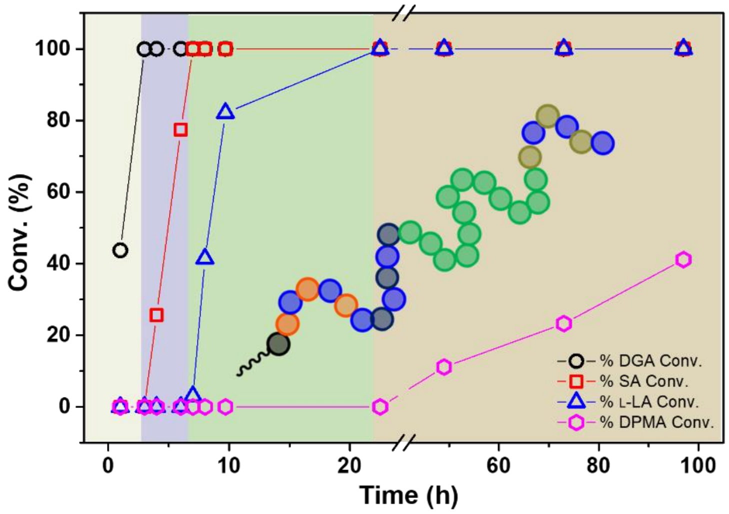

Supplementary Figure 51 | The plots of monomer conversion versus time. The polymerization of DGA, SA, DPMA, L-LA with BO was performed at 100 ℃ (entry 9 in Table 1).

Calculation method for degree of polymerization (DP) of each block

$DP_{P(DGA\text{-}alt\text{-}BO)}$ = 12.5 × conv. of DGA = 12.5 per block

$DP_{P(SA\text{-}alt\text{-}BO)}$ = (12.5 × conv. of SA)/2 = 6.3 per block

DP_{PLLA} = (37.5 × conv. of L-LA)/2 = 18.8 per block

$DP_{P(DPMA\text{-}alt\text{-}BO)}$ = (6.25 × conv. of DPMA)/2 = 1.3 per block

P(DPMA-*alt*-BO)$_{1.3}$-*b*-PLLA$_{18.8}$-*b*-P(SA-*alt*-BO)$_{6.3}$-*b*-P(DGA-*alt*-BO)$_{12.5}$-*b*-P(SA-*alt*-BO)$_{6.3}$-*b*-PLLA$_{18.8}$-*b*-P(DPMA-*alt*-BO)$_{1.3}$

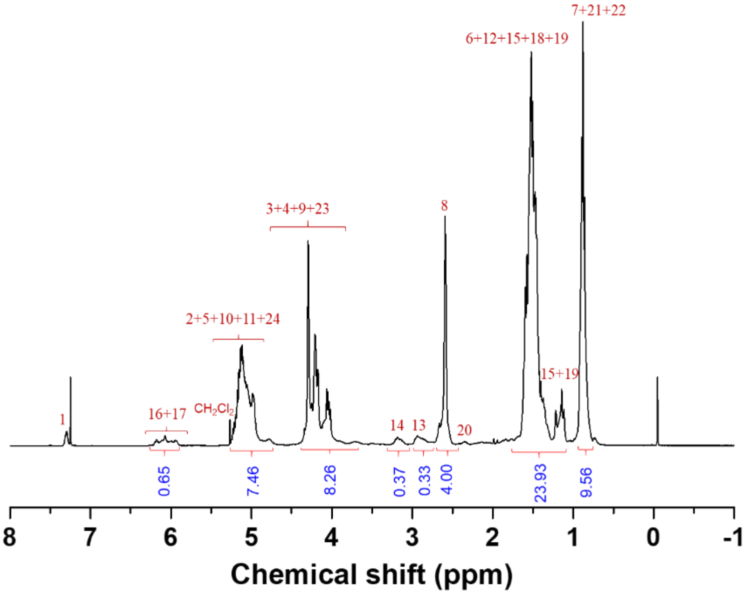

Supplementary Figure 52 | The ^{1}H NMR (CDCl$_3$) spectrum of the resultant heptablock polymer isolated from the mixture by precipitation (entry 9 in Table 1).

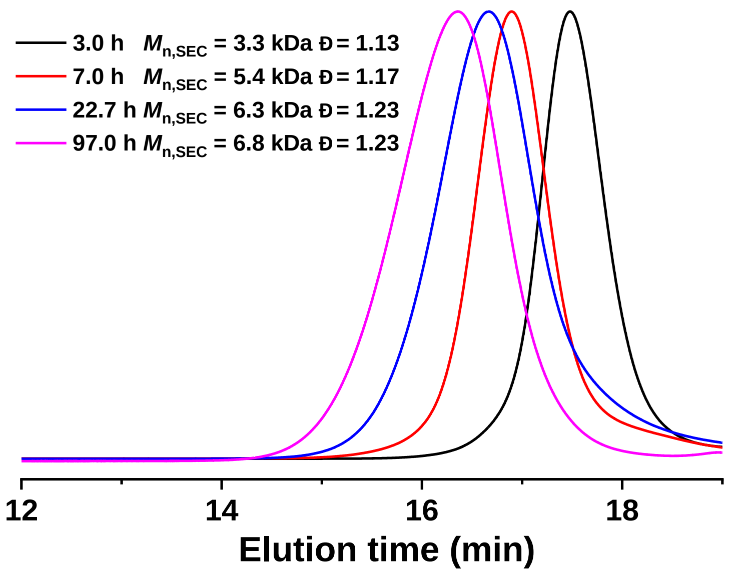

Supplementary Figure 53 | The SEC (THF) trace of the resultant heptablock polymers (entry 9 in Table 1).

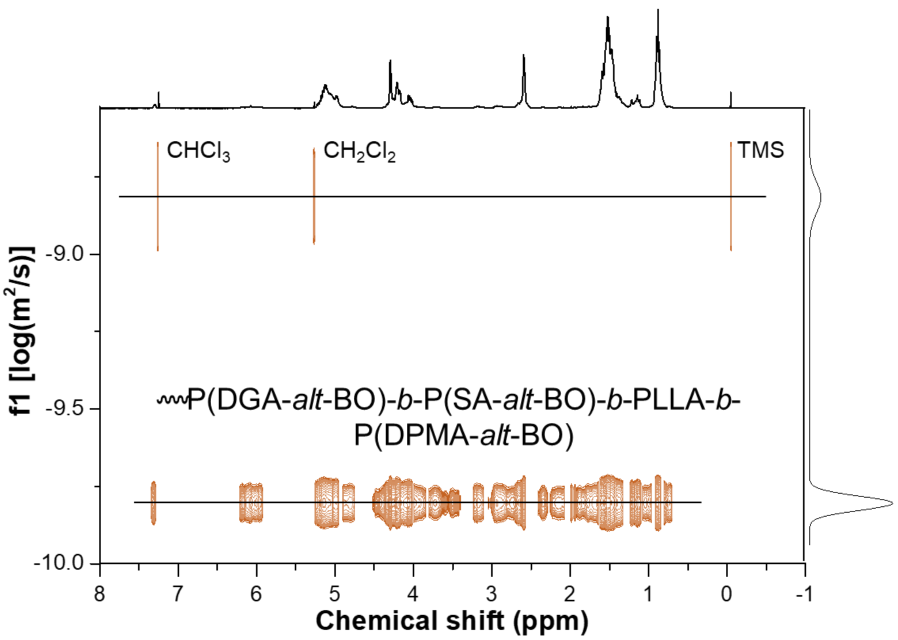

Supplementary Figure 54 | The DOSY NMR ($CDCl_3$) spectrum of the resultant heptablock polymer (entry 9 in Table 1).

The polymerization of DGA/SA/NA/L-LA/DPMA/BO initiated by PEG2000

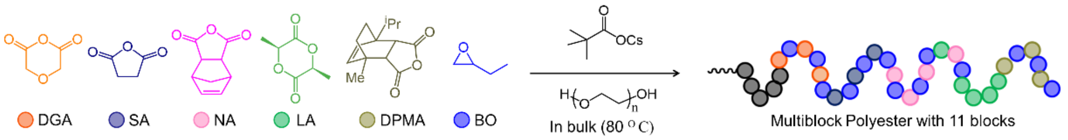

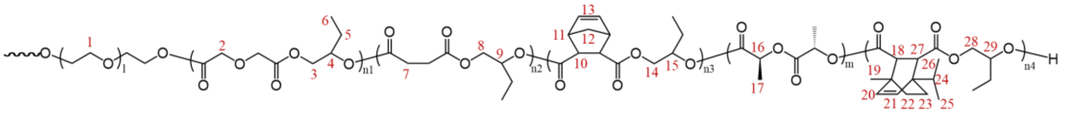

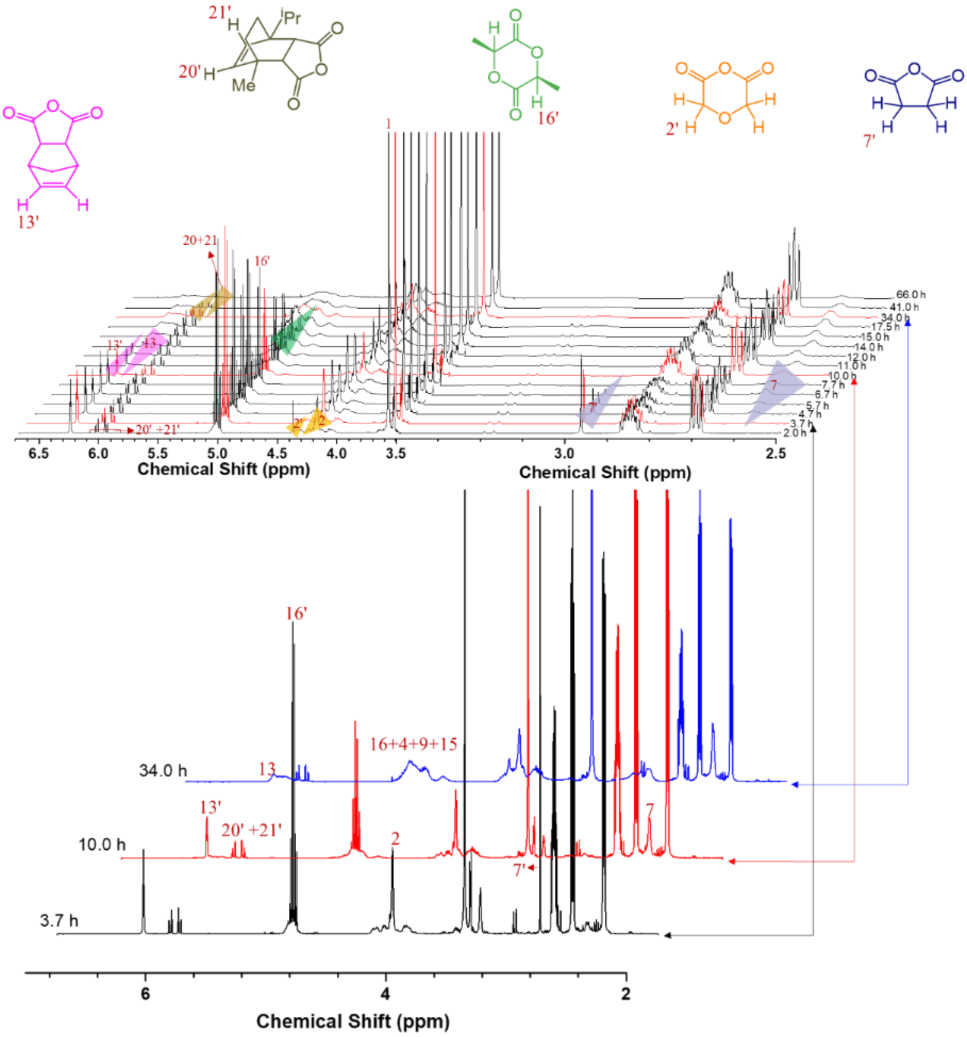

Supplementary Figure 55 | The ^{1}H NMR ($CDCl_3$) spectra of crude aliquots withdrawn from the reaction system for monitoring the conversion of DGA, SA, NA, DPMA, L-LA, and the formation of resultant polymers (entry 10 in Table 1).

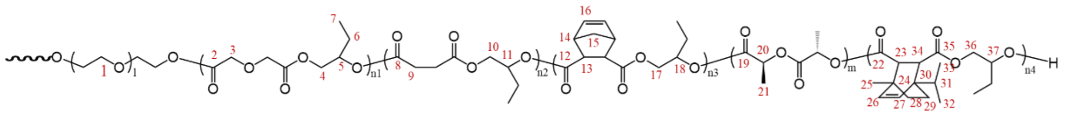

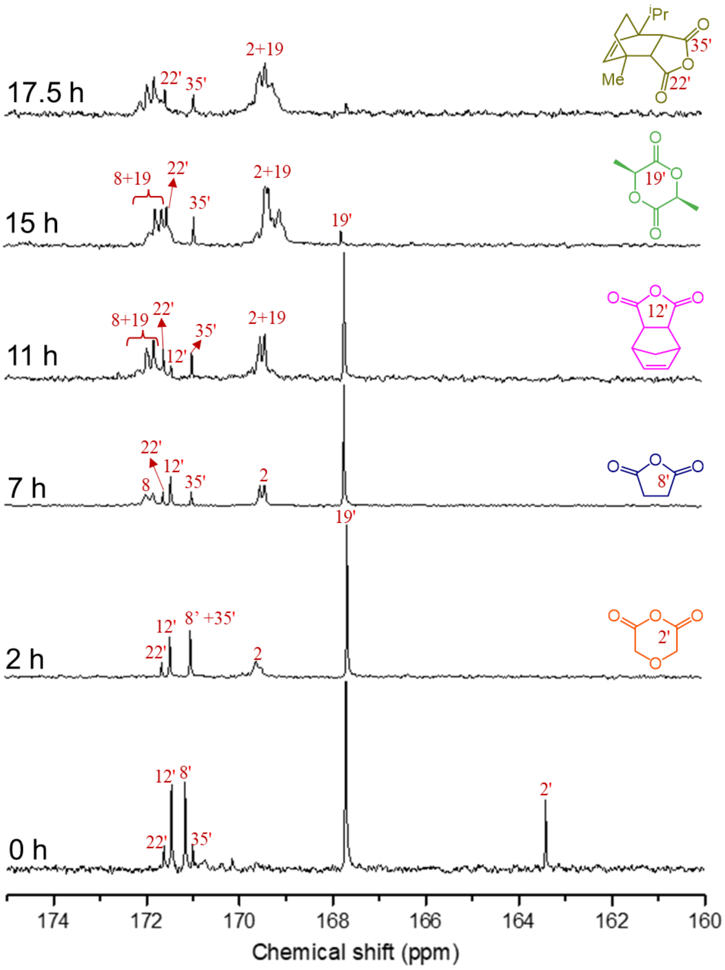

Supplementary Figure 56 | The ^{13}C NMR ($CDCl_3$) spectra of crude aliquots withdrawn from the reaction system for monitoring the conversion of DGA, SA, NA, DPMA, L-LA, and the formation of resultant polymers (entry 10 in Table 1).

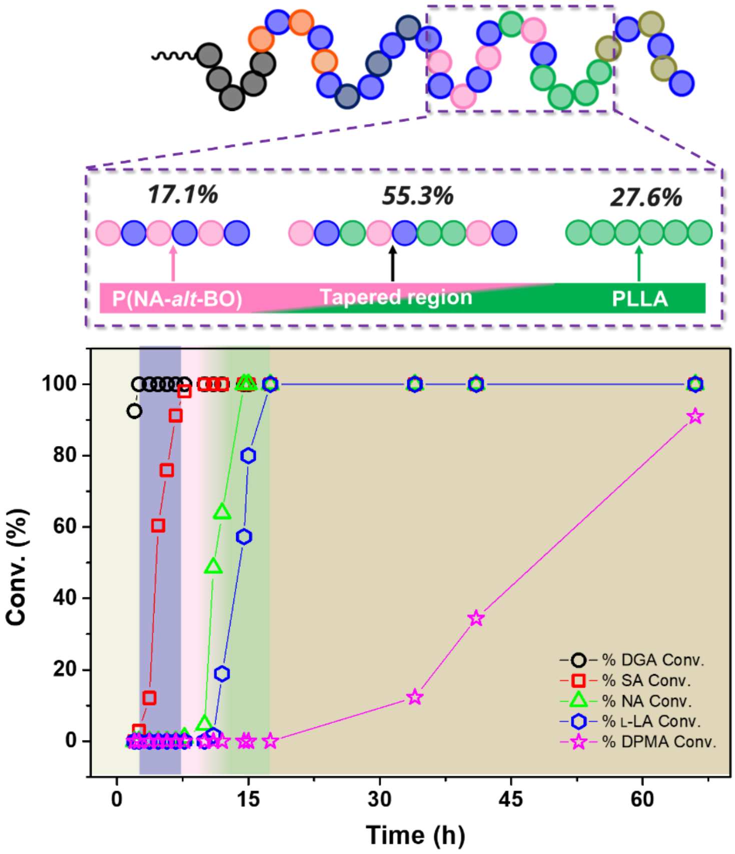

Supplementary Figure 57 | The plots of monomer conversion versus time. The polymerization of DGA, SA, NA, DPMA, L-LA with BO was performed at 80 ℃ (entry 10 in Table 1). The tapered region includes 51.5% of NA and 57.3% of L-LA, and the molar mass fraction of P(NA-*b*-BO) segment, tapered region, and PLLA segment are 17.1%, 55.3%, and 27.6%, respectively.

Calculation method for degree of polymerization (DP) of each block

$DP_{P(DGA\text{-}alt\text{-}BO)}$ = 12.5 × conv. of DGA = 6.3 per block

$DP_{P(SA\text{-}alt\text{-}BO)}$ = (12.5 × conv. of SA)/2 = 6.3 per block

$DP_{P(NA\text{-}alt\text{-}BO)}$ = (12.5 × conv. of NA)/2 × 0.485 = 3.0 per block

DP_{PLLA} = (37.5 × conv. of L-LA)/2 × 0.427 = 8.0 per block

$DP_{P(NA\text{-}alt\text{-}BO\text{-}grad\text{-}L\text{-}LA)}$ = (12.5 × conv. of NA)/2 × 0. 515 + (37.5 × conv. of L-LA)/2 × 0.573 = 14.0 per block

$DP_{P(DPMA\text{-}alt\text{-}BO)}$ = (6.25 × conv. of DPMA)/2 = 2.8 per block

P(DPMA-*alt*-BO)$_{2.8}$-*b*-PLLA$_{8.0}$-*b*-P(NA-*alt*-BO-*grad*-L-LA)$_{14.0}$-*b*-P(NA-*alt*-BO)$_{3.0}$-*b*-P(SA-*alt*-BO)$_{6.3}$-*b*-P(DGA-*alt*-BO)$_{6.3}$-*b*-PEG-*b*-P(DGA-*alt*-BO)$_{6.3}$-*b*-P(SA-*alt*-BO)$_{6.3}$-*b*-P(NA-*alt*-BO)$_{3.0}$-*b*-P(NA-*alt*-BO-*grad*-L-LA)$_{14.0}$-*b*-PLLA$_{8.0}$-*b*-P(DPMA-*alt*-BO)$_{2.8}$

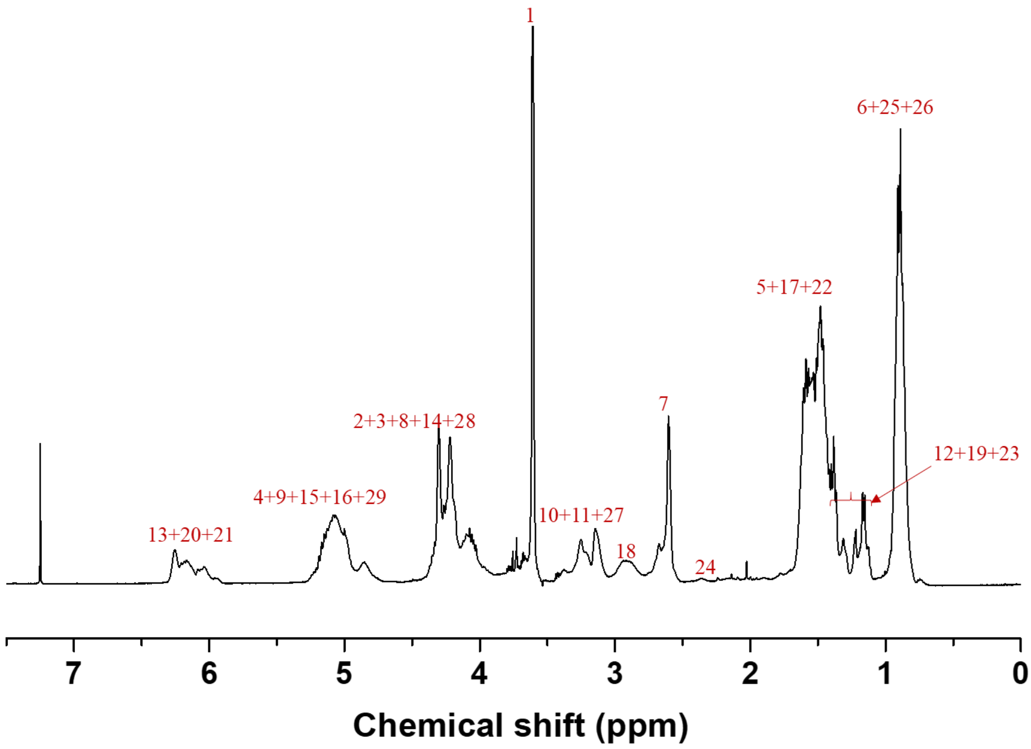

Supplementary Figure 58 | The ^{1}H NMR ($CDCl_3$) spectrum of the resultant deadlock polymer isolated from the mixture by precipitation (entry 10 in Table 1).

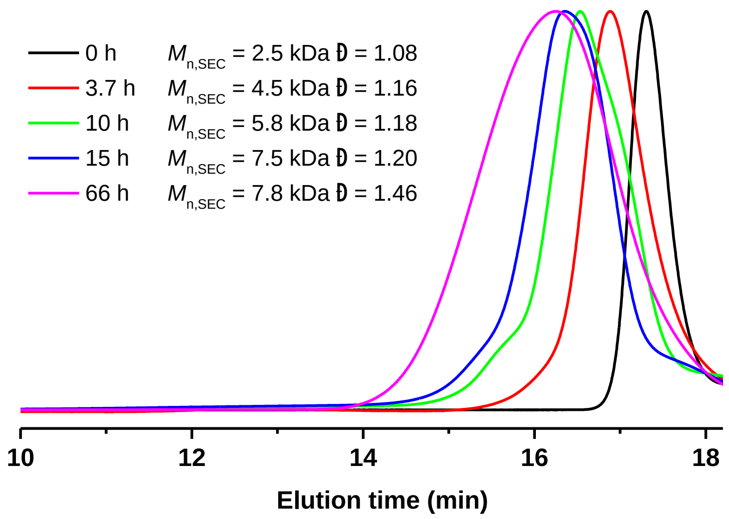

Supplementary Figure 59 | Evolution of SEC (THF) traces (entry 10 in Table 1).

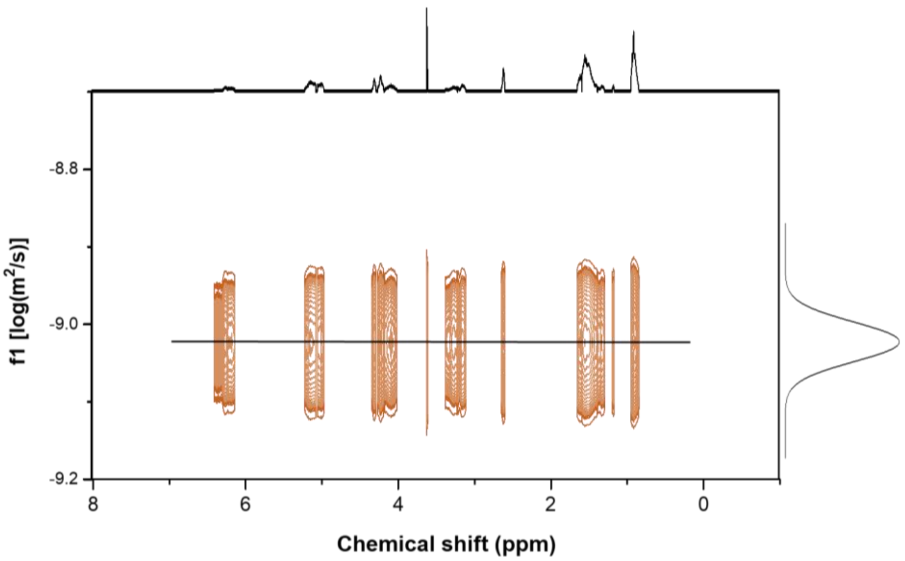

Supplementary Figure 60 | The DOSY NMR ($CDCl_3$) spectrum of the resultant deadlock polymer (entry 10 in Table 1).

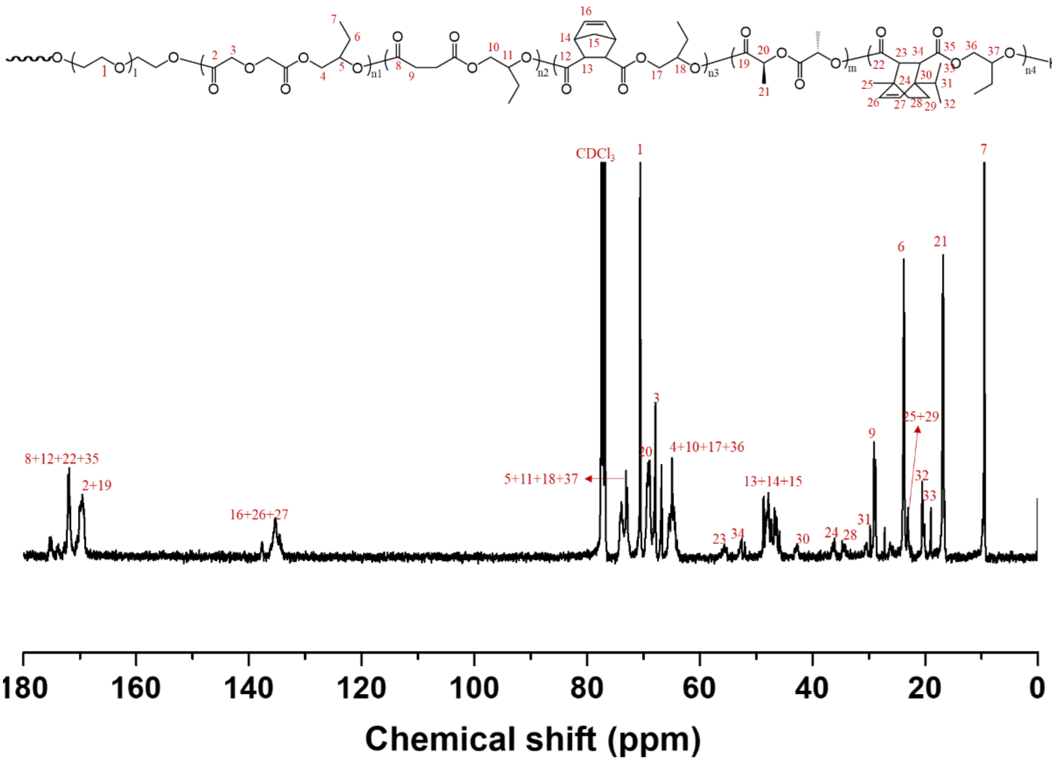

Supplementary Figure 61 | The ^{13}C NMR ($CDCl_3$) spectrum of the resultant deadlock polymer (entry 10 in Table 1). Chemical shifts from the carbonyl groups of P(SA-*alt*-epoxide), P(NA-*alt*-epoxide), and P(DPMA-*alt*-epoxide) locate at 172.5 ppm ~ 171.5 ppm, and Chemical shifts from the carbonyl groups of P(DGA-*alt*-epoxide) and PLLA locate at 169.1 ppm ~ 170.0 ppm.[4-6]

Supplementary Table 3 | ^{1}H NMR Peak Assignments for the resultant deadlock polymer in Supplementary Figure 58 (entry 10 in Table 1).

Group	1	2	3,8,14,28	4	5	6	7	9	10	11	12	13
^{1}H (δ ppm)	3.66 −3.54	4.27 −4.22	4.42 −3.83	5.11 −5.02	1.50 −1.32	0.97 −0.75	2.69 −2.58	5.02 −4.91	3.20 −3.06	3.33 −3.20	1.47 −1.25	6.37 −6.13
Group	15	16	17	18	19	20, 21	22,23	24	25, 26	27	29	
^{1}H (δ ppm)	4.91 −4.76	5.20 −5.00	1.67 −1.50	2.92 −2.76	1.44 −1.29	6.12– 5.96	1.86 −1.46	2.29 −2.08	1.01 −0.89	3.24 −3.08	4.88 −4.61	

Supplementary Table 4 | ^{13}C NMR Peak Assignments for the resultant deadlock polymer in Supplementary Figure 61 (entry 10 in Table 1).

Group	1	2	3	4,10,17,36	5,11,18,37	6	7	8	9	12
^{13}C (δ ppm)	70.5	169.5	67.9	65.7−64.1	74.6−72.6	23.7	9.5	171.5	28.9	172.0
Group	13	14	15	16	19	20	21	22	23	24
^{13}C (δ ppm)	47.2	48.0	46.4	135.5	169.9	69.0	16.7	172.3	55.6	52.4
Group	25	26,27	28	29	30	31	32	33	34	35
^{13}C (δ ppm)	23.0	134.6	34.4	22.8	42.7	30.5	20.4	18.8	52.7	172.5

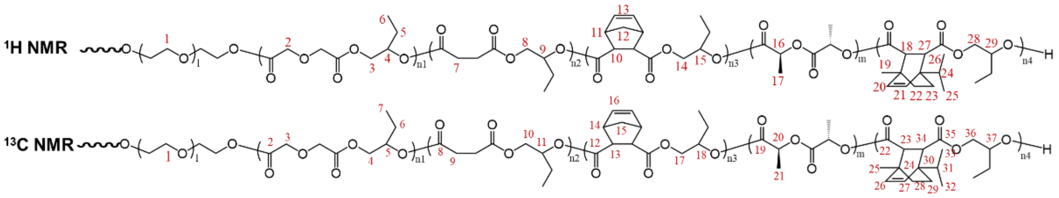

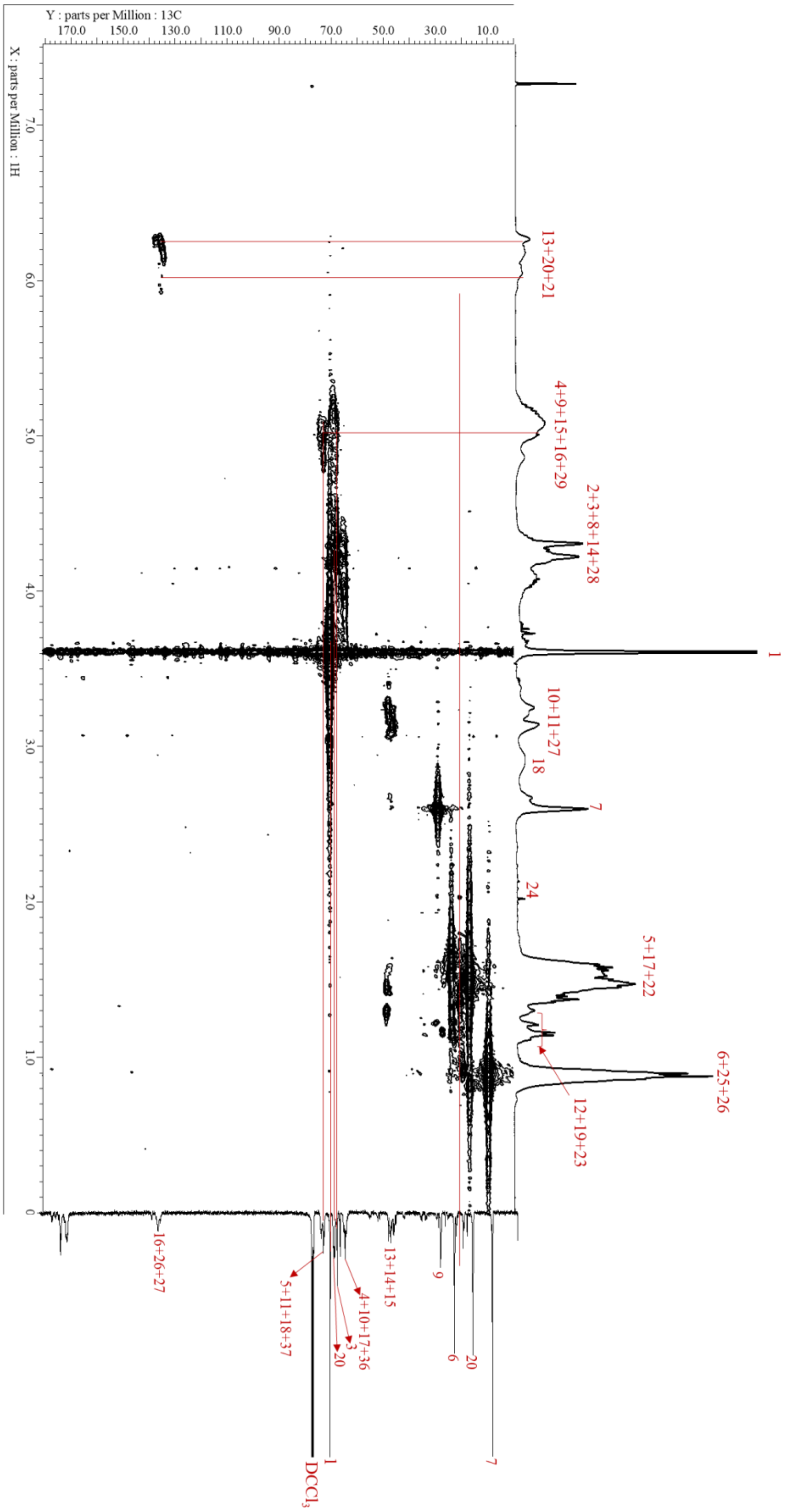

Supplementary Figure 62 | The HMQC NMR ($CDCl_3$) spectrum of the resultant deadlock polymer (entry 10 in Table 1).

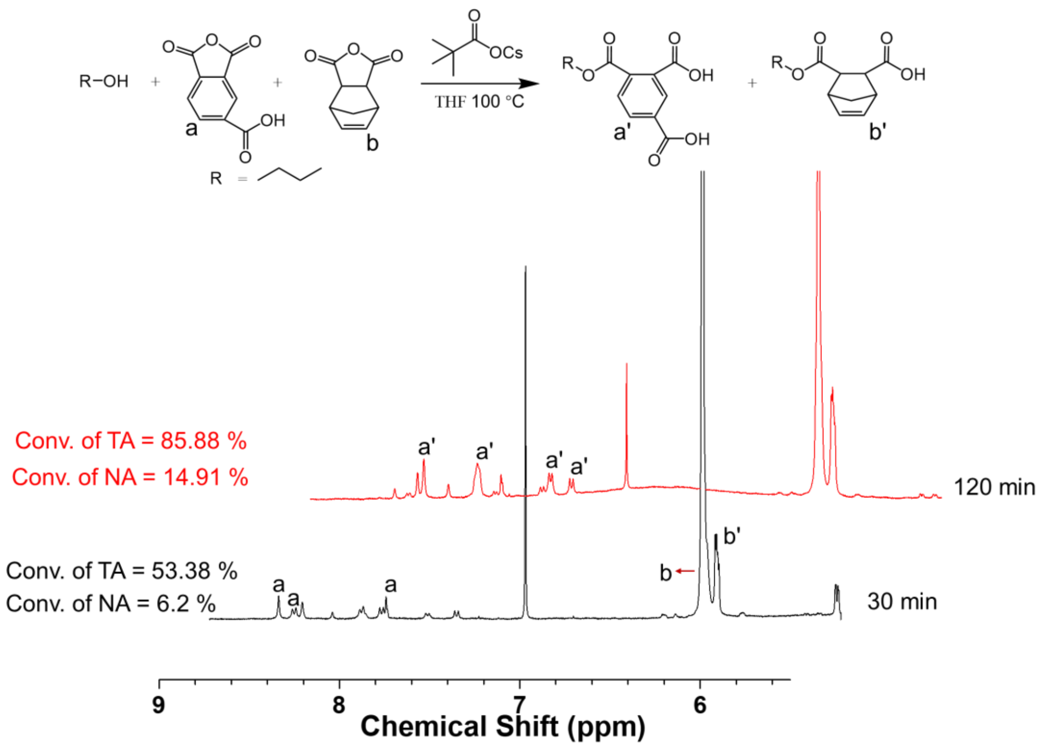

Supplementary Figure 63 | ^{1}H NMR ($CDCl_3$) spectrum of the product obtained from a mixture of TA and NA with cesium pivalate and *n*-butanol heated in THF at 100 °C for 30 min and 120 min, respectively, with the feed ratio of [*t*-$BuCO_2Cs$]/[*n*-butanol]/$[TA]_0$/$[NA]_0$ being 0.02/1/1/1. From the result, TA is far more reactive than NA.

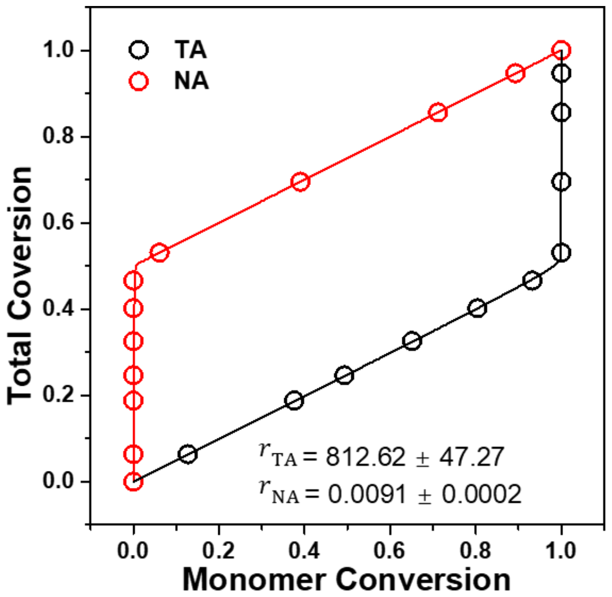

Supplementary Figure 64 | Total polymerization conversion plotted against monomer conversion the data were obtained from time-resolved ^{1}H NMR spectra of a cesium pivalate-catalyzed copolymerization of TA/NA/BO with a molar ratio of 25/25/150 at 80 °C. Solid black and red lines represent fits to the experimental data using the nonterminal model, eqs 1 and 2.

The polymerization of TA/NA/L-LA/DPMA/BO

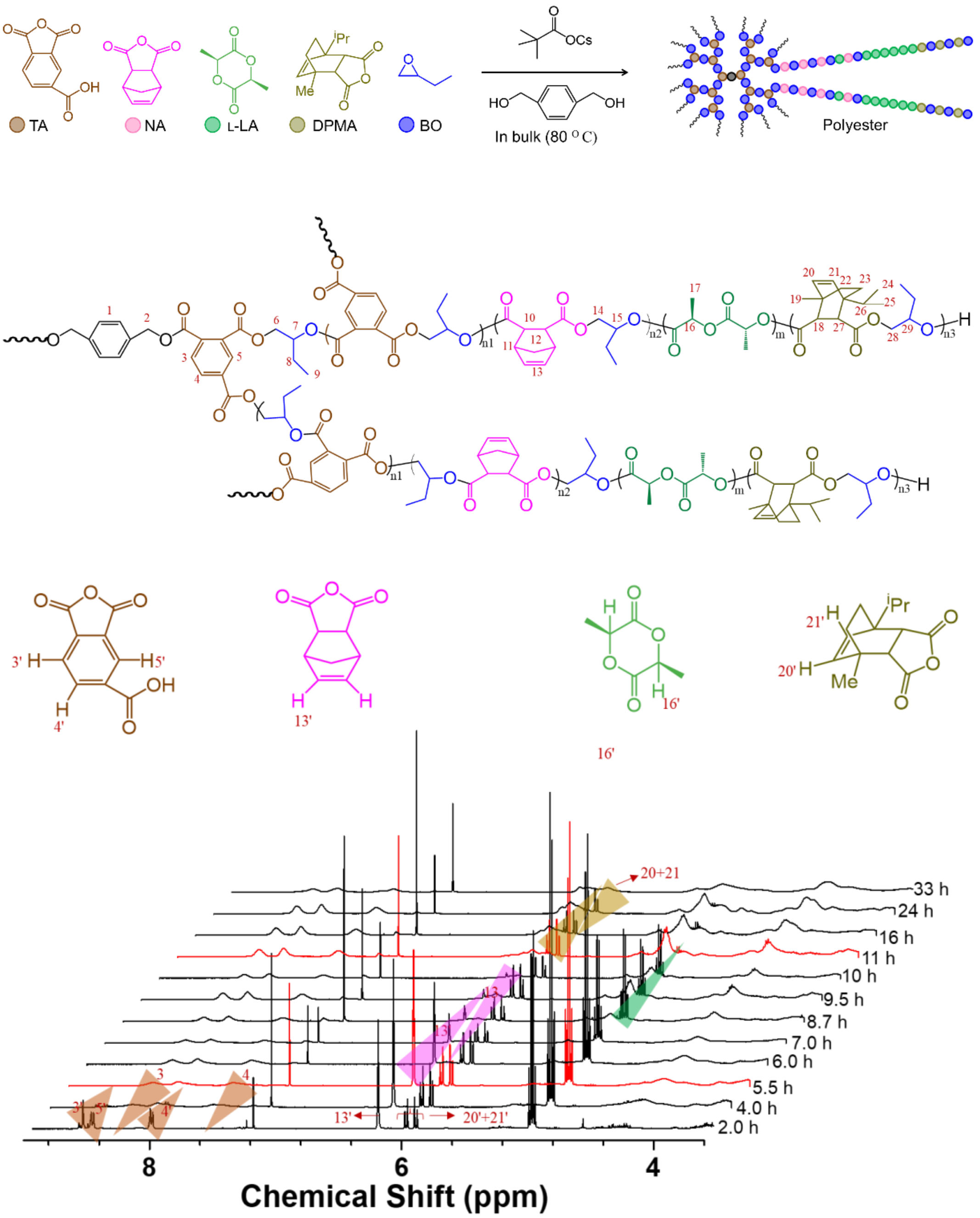

Supplementary Figure 65 | The ^{1}H NMR ($CDCl_3$) spectra of crude aliquots withdrawn from the reaction system for monitoring the conversion of TA, NA, DPMA, L-LA, and the formation of resultant polymers (entry 11 in Table 1).

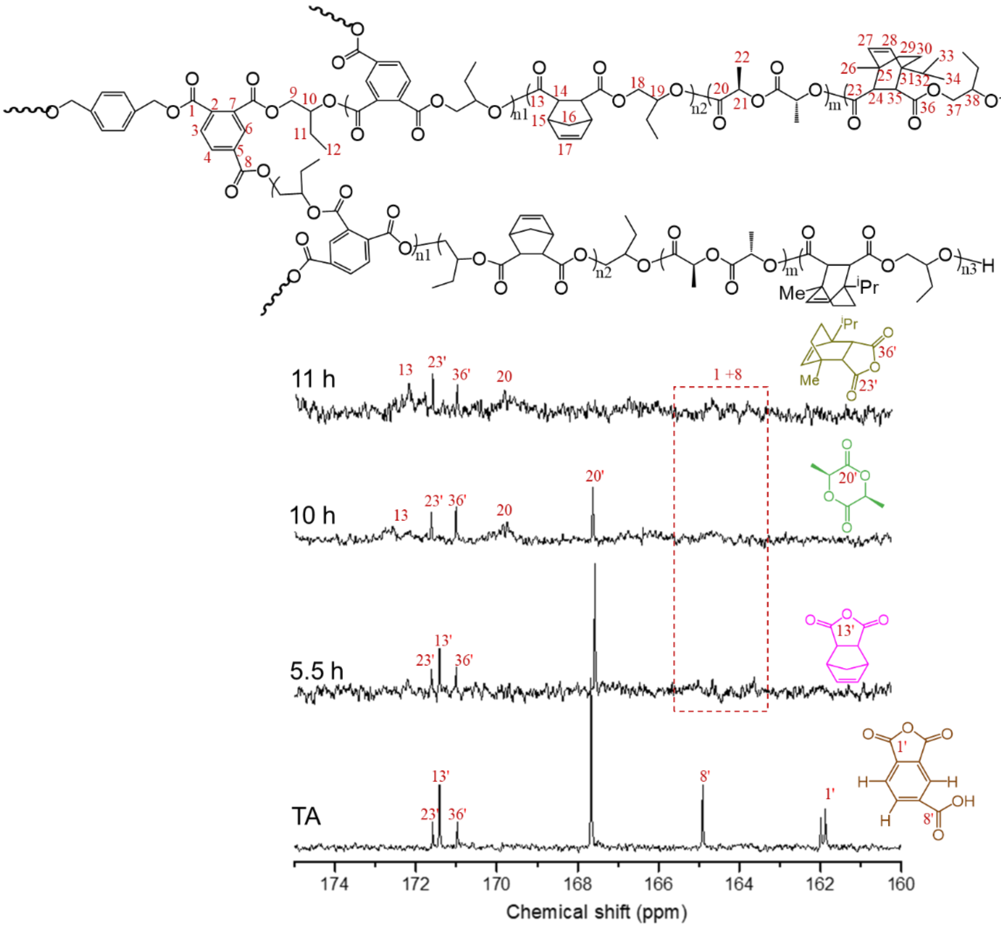

Supplementary Figure 66 | The ^{13}C NMR ($CDCl_3$) spectra of crude aliquots withdrawn from the reaction system for monitoring the conversion of TA, NA, DPMA, L-LA, and the formation of resultant polymers (entry 11 in Table 1).

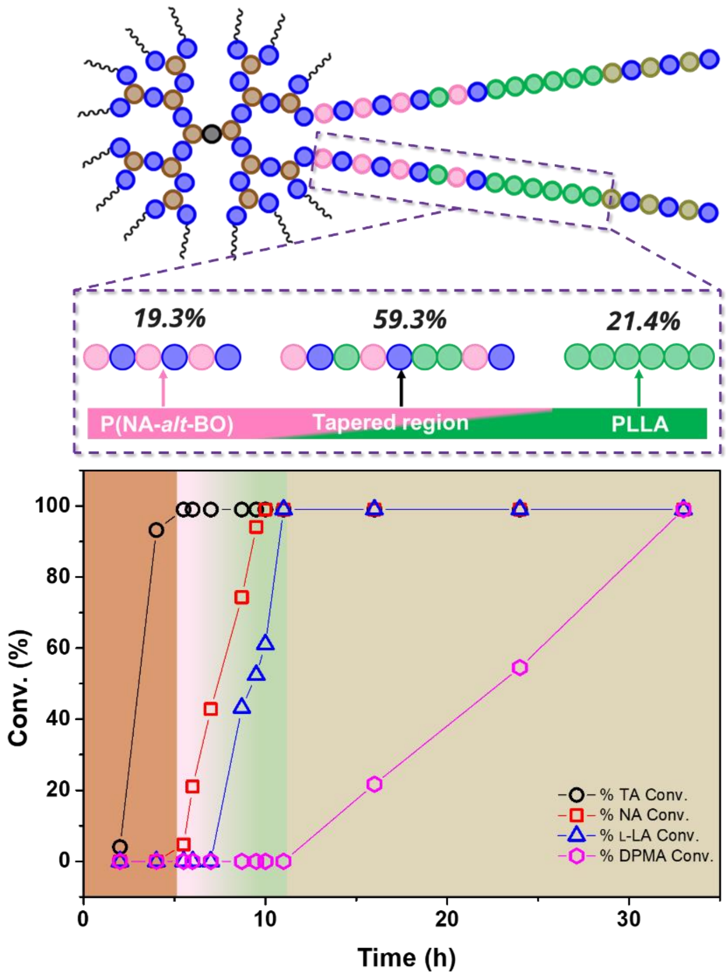

Supplementary Figure 67 | The plots of monomer conversion versus time. The polymerization of TA, NA, DPMA, L-LA with BO was performed at 80 ℃ (entry 11 in Table 1). The tapered region includes 57.2% of NA and 61.1% of L-LA, and the molar mass fraction of P(NA-*b*-BO) segment, tapered region, and PLLA segment are 19.3%, 59.3%, and 21.4%, respectively.

Calculation method for degree of polymerization (DP) of each monomer

DP_{TA} = 12.5 × conv. of TA = 12.5

DP_{NA} = 12.5 × conv. of NA = 12.5

$DP_{L\text{-}LA}$ = 25 × conv. of L-LA = 25

DP_{DPMA} = 6.25 × conv. of DPMA = 3.4

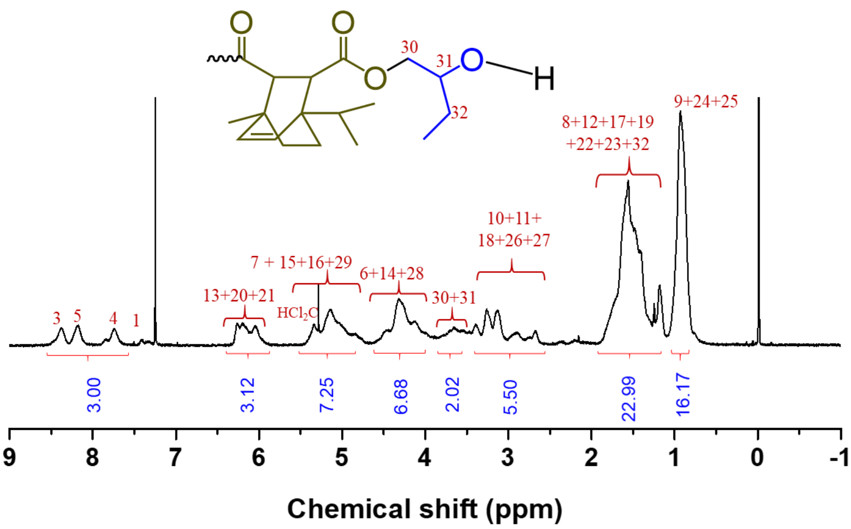

Supplementary Figure 68 | The ^1H NMR ($CDCl_3$) spectrum of the resultant polymer isolated from the mixture by precipitation (entry 11 in Table 1). DB = (D + T)/(D + T + L), DB: degree of branching; D: integral area of the peaks of the branching units (D = proton 7); T: integral area of the end groups (T = proton 31); L: integral area of the linear part (L = 2(proton 3 + proton 4 + proton 5)/3 - proton 7). DB = 0.68 and this value was calculated by the ^1H NMR spectrum.[7]

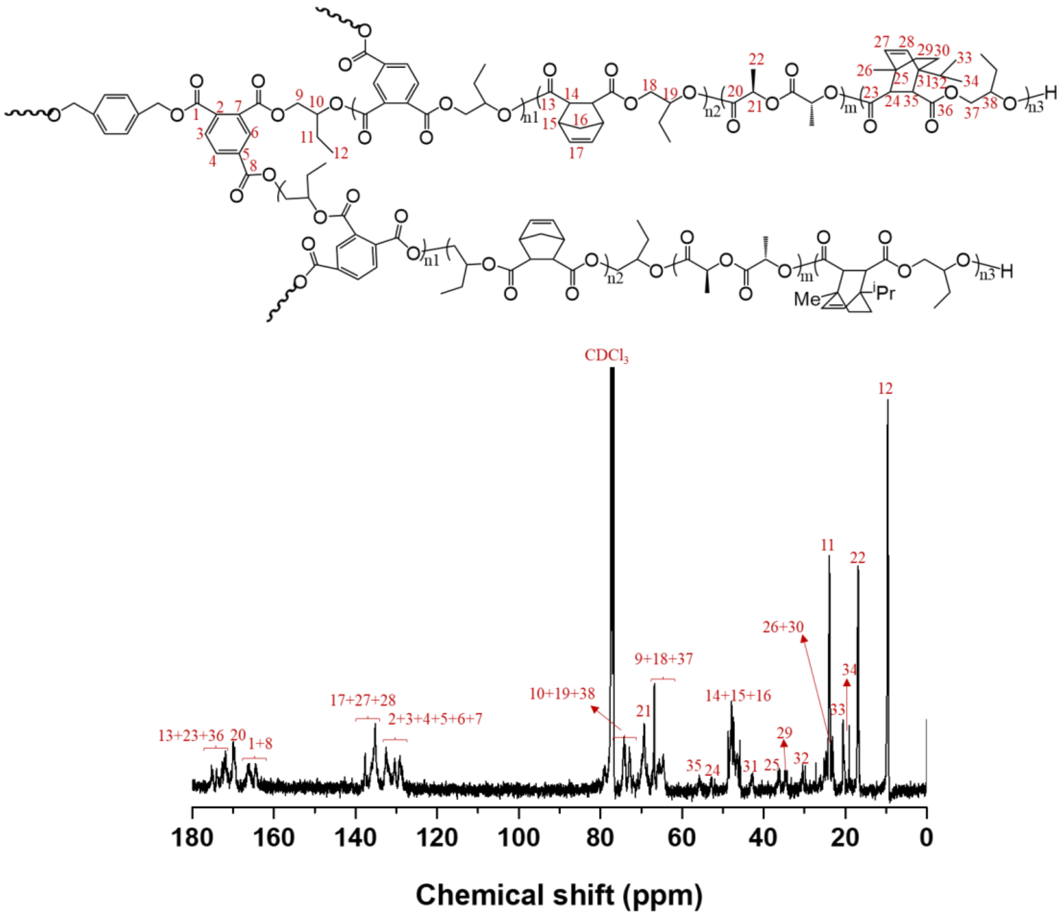

Supplementary Figure 69 | The ^{13}C NMR ($CDCl_3$) spectrum of the resultant polymer isolated from the mixture by precipitation (entry 11 in Table 1)

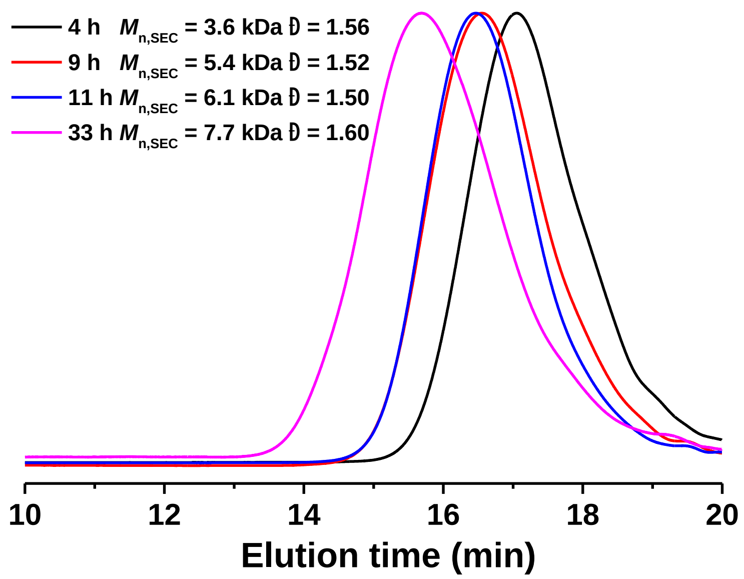

Supplementary Figure 70 | The SEC (THF) trace of the resultant polymers (entry 11 in Table 1).

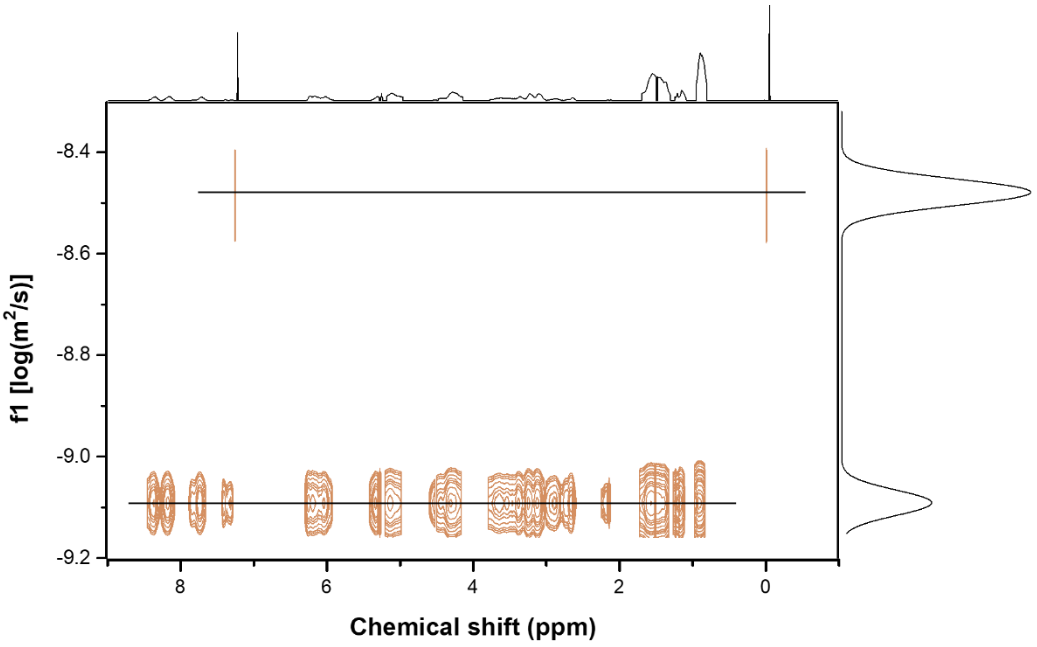

Supplementary Figure 71 | The DOSY NMR ($CDCl_3$) spectrum of the resultant polymer (entry 11 in Table 1).

The polymerization of TA/NA

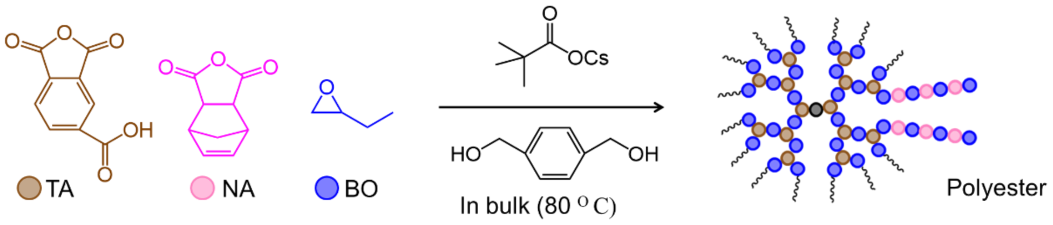

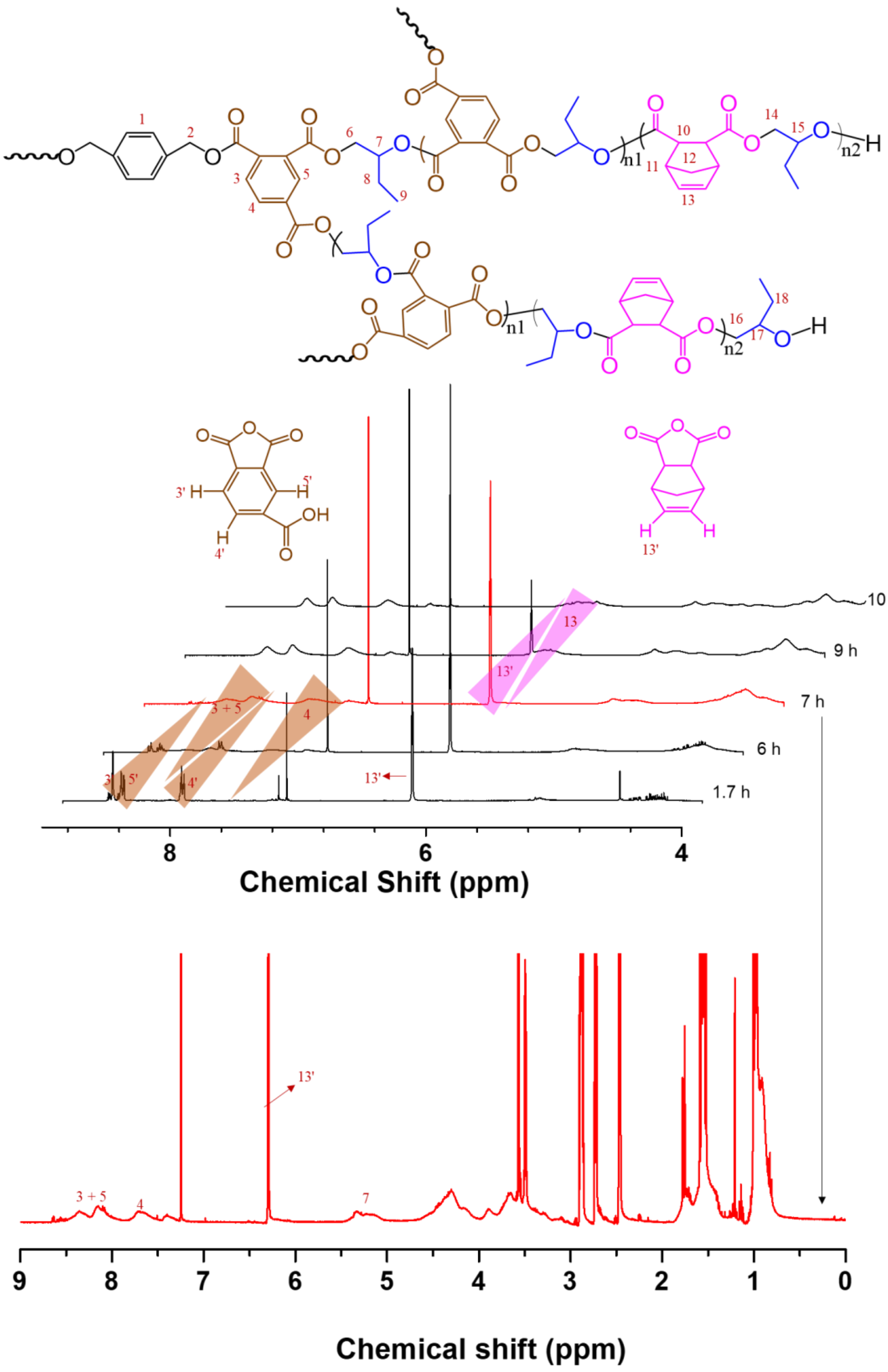

Supplementary Figure 72 | The ^{1}H NMR ($CDCl_3$) spectra of crude aliquots withdrawn from the reaction system for monitoring the conversion of TA, NA, and the formation of resultant polymers (entry 12 in Table 1).

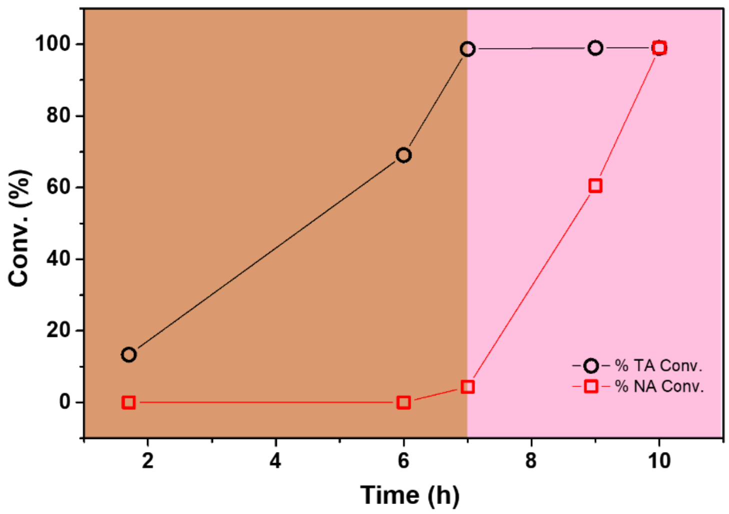

Supplementary Figure 73 | The plots of monomer conversion versus time. The polymerization of TA, NA with BO was performed at 80 ℃ (entry 12 in Table 1).

Calculation method for degree of polymerization (DP) of each monomer

DP_{TA} = 12.5 × conv. of TA = 12.5

DP_{NA} = 12.5 × conv. of NA = 7.6

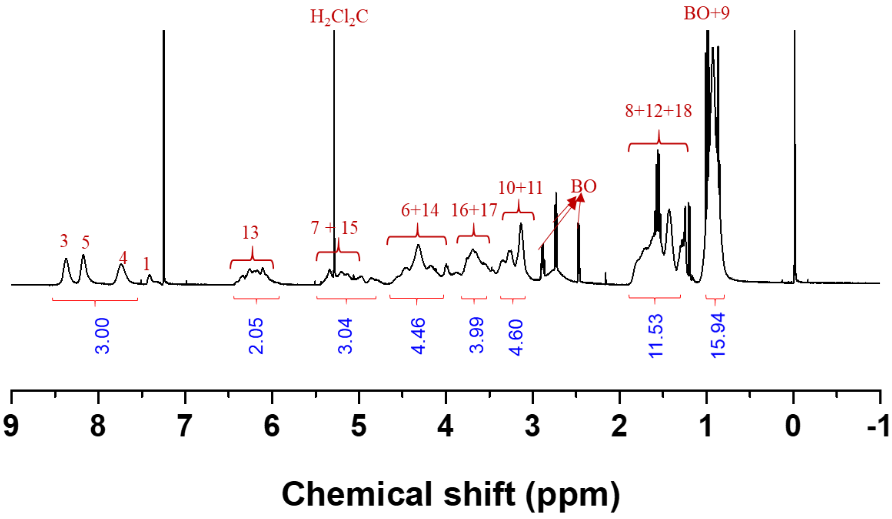

Supplementary Figure 74 | The ^{1}H NMR ($CDCl_3$) spectrum of the resultant polymer isolated from the mixture by precipitation (entry 12 in Table 1). DB = (D + T)/(D + T + L), DB: degree of branching; D: integral area of the peaks of the branching units (D = proton 7); T: integral area of the end groups (T = proton 17); L: integral area of the linear part (L = 2(proton 3 + proton 4 + proton 5)/3 - proton 7). DB = 0.72 and this value was calculated by the ^{1}H NMR spectrum.[7]

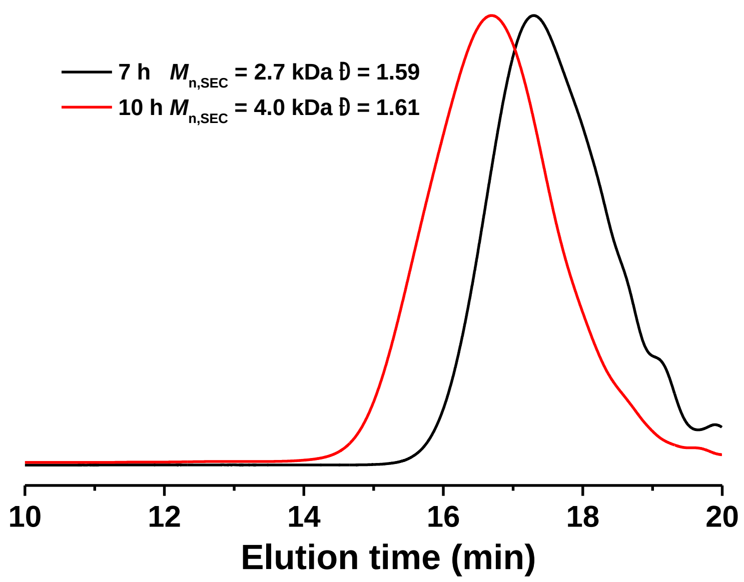

Supplementary Figure 75 | The SEC (THF) trace of the resultant polymers (entry 12 in Table 1).

The polymerization of TA/L-LA/DPMA/BO

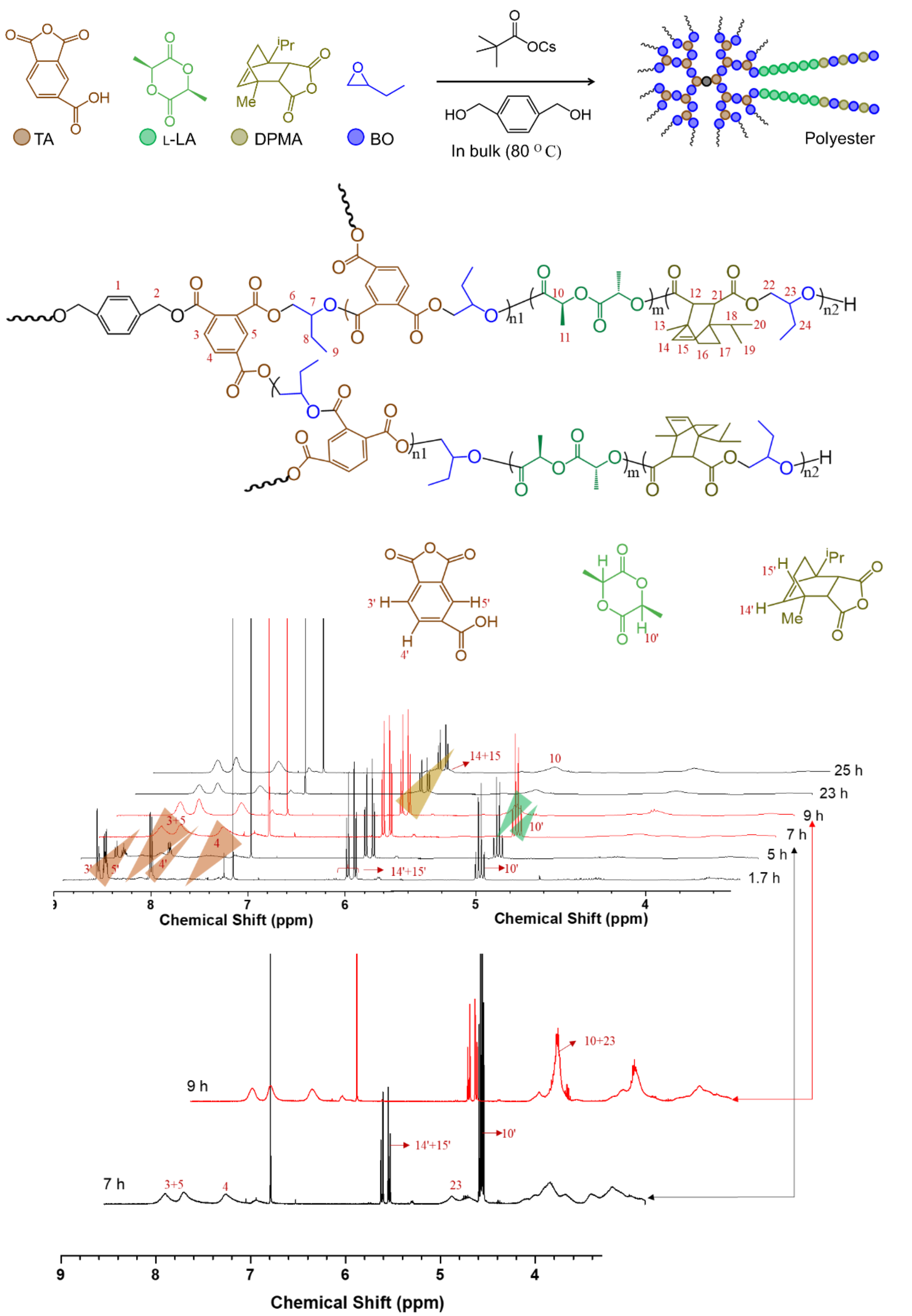

Supplementary Figure 76 | The ^{1}H NMR ($CDCl_3$) spectra of crude aliquots withdrawn from the reaction system for monitoring the conversion of TA, L-LA, DPMA and the formation of resultant polymers (entry 13 in Table 1).

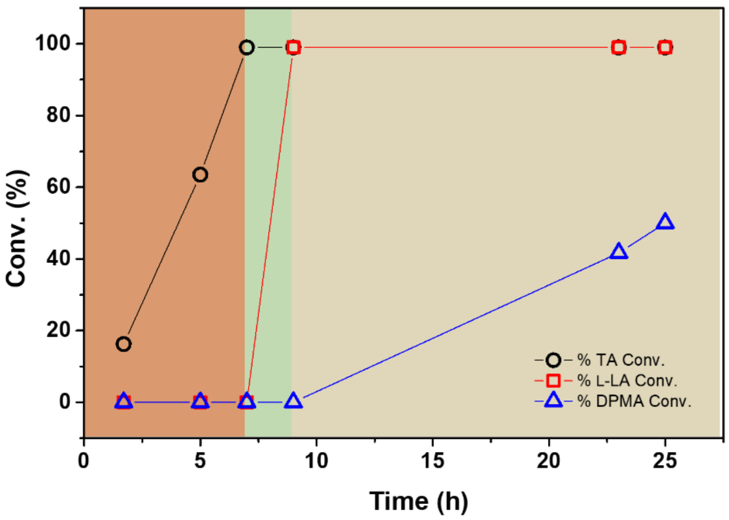

Supplementary Figure 77 | The plots of monomer conversion versus time. The polymerization of TA, DPMA, L-LA with BO was performed at 80 ℃ (entry 13 in Table 1).

Calculation method for degree of polymerization (DP) of each monomer

DP_{TA} = 12.5 × conv. of TA = 12.5

DP_{L-LA} = 25 × conv. of L-LA = 25

DP_{DPMA} = 6.25 × conv. of DPMA = 3

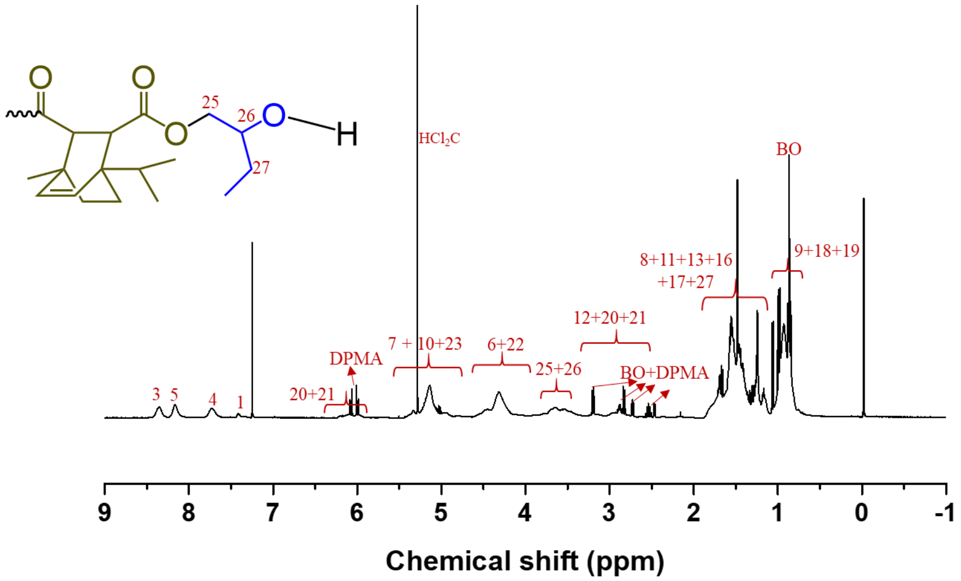

Supplementary Figure 78 | The ^{1}H NMR ($CDCl_3$) spectrum of the resultant polymer isolated from the mixture by precipitation (entry 13 in Table 1). DB = (D + T)/(D + T + L), DB: degree of branching; D: integral area of the peaks of the branching units (D = proton 7); T: integral area of the end groups (T = proton 26); L: integral area of the linear part (L = 2(proton 3 + proton 4 + proton 5)/3 - proton 7). DB = 0.69 and this value was calculated by the ^{1}H NMR spectrum.[7]

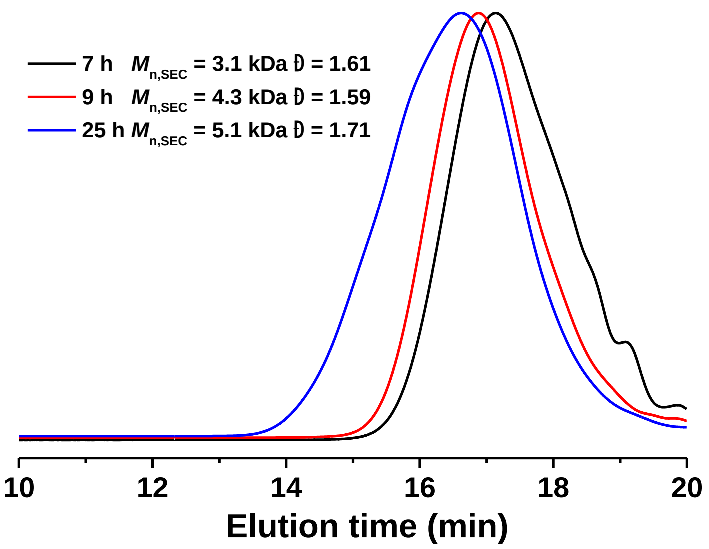

Supplementary Figure 79 | The SEC (THF) trace of the resultant polymers (entry 13 in Table 1).

Supplementary References

1. Beckingham, B. S., Sanoja, G. E., & Lynd, N. A. Simple and accurate determination of reactivity ratios using a nonterminal model of chain copolymerization. Macromolecules, 2015, 48(19), 6922-6930.

2. Chwatko, M., & Lynd, N. A. Statistical copolymerization of epoxides and lactones to high molecular weight. Macromolecules, 2017, 50(7), 2714-2723.

3. Van Zee, N. J.; Coates, G. W. Alternating Copolymerization of Propylene Oxide with Biorenewable Terpene-Based Cyclic Anhydrides: A Sustainable Route to Aliphatic Polyesters with High Glass Transition Temperatures. Angewandte Chemie-International Edition. 2015, 127(9), 2703-2706.

4. Ji, H.-Y., Wang, B., Pan, L., Li, Y.-S. One-step access to sequence-controlled block copolymers by self-switchable organocatalytic multicomponent polymerization. Angewandte Chemie International Edition, 57, 16888–16892 (2018).

5. Xia, X. C., Ryota, S., Takojima, K., Jiang, D. H., Isono, T., Satoh, T., Smart access to sequentially and architecturally controlled block polymers via a simple catalytic polymerization system. ACS Catalysis, 11, 5999-6009 (2021).

6. Sanford, M. J., Pena Carrodeguas, L., Van Zee, N. J., Kleij, A. W., & Coates, G. W. Alternating copolymerization of propylene oxide and cyclohexene oxide with tricyclic anhydrides: access to partially renewable aliphatic polyesters with high glass transition temperatures. Macromolecules, 49(17), 6394-6400 (2016).

7. Fornof, A. R.; Glass, T. E.; Long, T. E. J. M. C.; Physics, Degree of branching of highly branched polyurethanes synthesized via the oligomeric A2 plus B3 methodology. Macromolecular Chemistry and Physics 2006, 207 (14), 1197-1206.
